# Impact of pharmacy-supported interventions on proportion of patients receiving non-indicated acid suppressive therapy upon discharge: A systematic review and meta-analysis

**DOI:** 10.1371/journal.pone.0243134

**Published:** 2020-12-03

**Authors:** Devada Singh-Franco, David R. Mastropietro, Miriam Metzner, Michael D. Dressler, Amneh Fares, Melinda Johnson, Daisy De La Rosa, William R. Wolowich

**Affiliations:** 1 Department of Pharmacy Practice, Nova Southeastern University, College of Pharmacy, Fort Lauderdale, Florida, United States of America; 2 Department of Pharmaceutical Sciences, Nova Southeastern University, Fort Lauderdale, Florida, United States of America; 3 Martin and Gail Press Health Professions Division Library, Nova Southeastern University, Fort Lauderdale, Florida, United States of America; Auburn University, UNITED STATES

## Abstract

**Objective:**

Conduct a systematic review and meta-analysis to estimate the impact of pharmacy-supported interventions on the proportion of patients discharged from the hospital on inappropriate acid suppressive therapy (AST).

**Methods:**

To identify studies, the following databases were systematically searched on October 14^th^, 2018 and repeated on September 12^th^, 2019: Ovid MEDLINE(R) and In-Process & Other Non-Indexed Citations and Daily, Embase.com, CINAHL, Web of Science, Cochrane CENTRAL (EBSCO), and ClinicalTrials.gov. Eligible studies consisted of adults, intervention and historical/usual care groups, description of active pharmacy-supported intervention, and proportion of patients discharged on inappropriate AST. Qualitative assessments and quantitative analyses were performed. Modified funnel plot analysis assessed heterogeneity. Preferred reporting items of systematic reviews and meta-analyses (PRISMA) methodology was used to evaluate studies in this review.

**Results:**

Seventeen publications resulting in 16 studies were included in the review. Using random effects model, meta-analysis showed a significant reduction in the odds of being discharged on inappropriate AST from the hospital in the pharmacist-supported intervention arm versus comparator (Odds Ratio 0.33 [95%CI 0.20 to 0.53]), with significant heterogeneity (*I*^*2*^ = 86%). Eleven studies favored pharmacy-supported interventions, four were inconclusive and one favored usual care. Using modified funnel plot analysis, our final evaluation was distilled to 11 studies and revealed a similar outcome (OR 0.36 [95%CI 0.27 to 0.48]), but with less heterogeneity (*I*^*2*^ = 36%).

**Conclusion:**

This systematic review and meta-analysis showed that pharmacy-supported interventions were associated with a significantly reduced probability of patients discharged on inappropriate AST. However, heterogeneity was high and may affect interpretation of results. Using funnel plot optimization method, three positive and two negative studies were objectively removed from analyses, resulting in a similar effect size, but with less heterogeneity. To improve study quality, future researchers should consider utilizing a pre-post, multi-arm, prospective design with sampling randomization, training of data extractors (preferably two extractors), re-evaluating a small dataset to check for agreement and providing a comprehensive methodology in subsequent publications.

## Introduction

Proton pump inhibitors (PPIs) are prescribed for the management of reflux disease [1, 2], dyspepsia [3], nonvariceal upper gastrointestinal (GI) bleeds (GIB) [4], and prevention against upper GIB in hospitalized patients [5–7]. These agents are also recommended to reduce GI bleed/ulcer risk of antiplatelet therapy and non-steroidal anti-inflammatory drugs (NSAIDs) [8, 9], and to augment eradication of *H*. *pylori* infection [10, 11]. In certain clinical situations, PPIs may be indicated for chronic use: gastroesophageal reflux disease (GERD) not responding to H_2_-receptor antagonists (H_2_RAs), severe erosive esophagitis, Barrett’s esophagus, chronic NSAID use, and a documented history of bleeding gastric ulcer [12, 13].

Patients in the intensive care unit (ICU) setting are at risk for stress-related mucosal damage (SRMD) with subsequent upper GIB [5, 7, 14]. Factors such as splanchnic hypoperfusion or impaired microcirculation due to hypovolemia or shock in critically ill patients can lead to an impairment in the integrity of the GI mucosa, resulting in acid exposure, ulceration and bleeding [5, 7, 14]. Mucosal lesions are typically asymptomatic, numerous, located in the proximal stomach, and unlikely to perforate [5, 14]. However, the mortality rate associated with clinically-important SRMB in ICU patients ventilated for > 48 hours is high (~20–30% increase in relative risk) [15].

Stress ulcer prophylaxis (SUP) guidelines provide criteria for which patients at risk for a GI event would most likely benefit from the addition of either a PPI or H_2_RA [5, 7, 16]. The most recently published guideline on GIB prophylaxis for critically ill patients categorized GIB risks to assist health care providers in determining which patients are SUP candidates [16]. Patients with a GIB risk ≥4%, without other indications for gastric acid suppressive therapy (AST), should receive a PPI or H_2_RA, rather than no prophylaxis. Highest-risk patients (8–10%) are those who are on mechanical ventilation (without enteral nutrition) or have chronic liver disease; high-risk patients (4–8%) have coagulopathy or have ≥ 2 of the following risk factors: mechanical ventilation with enteral nutrition, acute kidney injury, sepsis, or shock [16].

In critically ill patients at moderate (2% to 4%) or low (below 2%) GIB risk, without other indications for AST, no prophylaxis is recommended. Patients with moderate risk factors (2–4%) are those who have only one of the following factors: mechanical ventilation with enteral nutrition, have acute kidney injury, sepsis, or shock. Low-risk patients (1–2%) are those who are critically ill but have no risk factors, have acute hepatic failure, use steroids or are on immunosuppression therapy, use anticoagulants (vitamin K antagonists, direct acting oral anticoagulants, therapeutic doses of unfractionated or low molecular weight heparin, intravenous direct thrombin (II) inhibitors, adenosine diphosphate receptor inhibitor and similar drugs), have cancer, or are male [16].

Other publications [5, 7, 8, 14, 17] have reported additional risk factors where a patient might benefit from SUP and these include those with 1) Glasgow coma score ≤10 or the inability of patient to obey simple commands; 2) thermal injury involving >35% of body surface area; 3) partial hepatectomy, hepatic, or renal transplantation candidates; 4) trauma patients with injury severity score of ≥16; 5) mechanical ventilation >48 hours; 6) history of gastric ulcer or bleeding during the year before admission to the hospital; 7) spinal cord injury; 8) renal failure; 9) presence of ≥ two of the following: sepsis; ICU stay >1 week; occult or overt bleeding for ≥ 6 days; corticosteroid therapy (>250 mg hydrocortisone or equivalent daily) and/or 10) being on oral anticoagulants, antiplatelet therapy and/or NSAIDs. As there are multiple risk factors and the relative importance of each remain unclear [18], institution-specific guidelines will incorporate these risks based on a review of the published literature in combination with the clinical experience of the health care providers in care of the patients within their communities.

In the non-ICU setting, there is no universally-accepted consensus on when a patient should be placed on AST for SUP. Additionally, GIB risk is lower in non-ICU settings compared with ICU setting [7]. The 1999 American Society of Health-System Pharmacists (ASHP) SUP guidelines recommend not initiating SUP in patients with fewer than two risk factors for clinically important bleeding [5, 7]. In patients on indicated AST prior to arrival, consideration is given to either maintain or temporarily discontinue AST; appropriate indications include (but are not limited to) Barrett's esophagus, recent GIB, erosive esophagitis, persistent GERD symptoms, dyspepsia, bariatric surgery, advanced age, and/or on medications associated with GIBs (e.g., aspirin, NSAIDs, dual antiplatelet therapy, anticoagulants, corticosteroids) [3, 8, 9, 14, 17, 19]. In a hospitalized patient not on AST prior to arrival, studies conducted to determine appropriate and inappropriate PPI use in non-critical patients provides criteria on what is an appropriate indication for AST (newly-started AST for acute GIB, for example), and if an indication was not found, then the patient was considered to be on inappropriate AST. Criteria used in non-ICU settings were typically derived from SUP and other guidelines [3, 5, 8, 9, 14, 17, 19, 20].

Regardless of whether SUP was initiated appropriately or inappropriately within the ICU and non-ICU settings or patients were already on AST (either appropriately or inappropriately) prior to admission, many are discharged on AST inappropriately (without an indication) [21–26]. In Ontario, 6.1% of 556,323 patients were discharged on AST without a documented indication. About 15,000 patients continued AST for over one year at a cost of over $10 million Canadian. Of these, only 21% developed an acceptable indication during the year [21]. In Italy, clinical evaluation of 1,081 patients at discharge showed overprescribing in 30% of patients receiving PPIs from seven geriatric acute care medical wards [22]. In the United States (US), a retrospective review of a large medical records and pharmacy prescription database (Blue Cross of Northeastern Pennsylvania) during 2005–2008 showed a significant reduction in the volume of PPI prescriptions after discharge, but a high proportion (78–82%) remained inappropriate, with an estimated cost of US $595,809 [23].

Recently, multiple observational studies show that PPIs are associated with a number of adverse events including nosocomial pneumonia and *C*. *difficile* infection within the hospital setting. With prolonged use, there is increased risk of bone fractures, chronic kidney disease, community-acquired pneumonia, *C*. *difficile* infection, mineral and vitamin deficiencies (vitamin B12, iron, magnesium) [12, 16, 27–30].

Inappropriate prescribing within the hospital setting and a growing list of serious adverse events associated with PPIs have prompted health care providers to develop various strategies to reduce inappropriate use [31]. These include deprescribing [12, 32–34], applying SUP guidelines, checklists and protocols [35–38], completing medication reconciliation activities [39, 40], communicating concerns in-person and/or electronically [41–44], academic detailing [45], and educational campaigns [36, 44, 46, 47].

Clinical pharmacists focus on optimizing medication therapy and use therapeutic knowledge, experience, and judgment to ensure optimal patient outcomes through comprehensive medication management―where each medication is individually assessed with respect to its appropriateness, effectiveness, and safety [48]. Several recent publications demonstrate the benefit of pharmacist-supported interventions in ICU and non-ICU setting settings. Lee et al. found that critical care pharmacist participation in multidisciplinary team care in ICU patients was associated with reduced mortality (Odds Ratio (OR) 0.78 [95% Confidence Interval (CI) 0.73 to 0.83], reduced ICU length of stay (mean difference –1.12 days [95% CI –1.52 to –0.72], preventable adverse events (OR 0.26 [95% CI 0.15 to 0.44] and nonpreventable adverse events (not due to medication errors) (OR 0.47 [95% CI 0.28 to 0.77) [49]. Naseralallah et al. found in a systematic review that pharmacist involvement in education, direct patient care, therapeutic drug monitoring, drug distribution oversight and quality improvement was associated with reduced rates of medication errors in hospitalized pediatric patients [50]. A recent meta-analysis of 32 studies by Rodrigues et al. comparing pharmacy-supported transitions-of-care interventions versus usual care found a significant reduction of 30-day all-cause readmissions (OR 0.68 [95% CI 0.61 to 0.75]) [51]. Common pharmacy-supported interventions included patient counseling, medication reconciliation, and patient-centered follow-up (e.g., telephone call, home and/or clinic visits) with interventions occurring post-discharge (80%) or during discharge (57%) to home [51]. Lastly, a lack of clinical pharmacy services in the ICU setting was associated with significantly greater Medicare charges, medication charges, complications related to disease state, ICU length of stay and mortality [52, 53].

A systematic review of PPI deprescribing to determine the effectiveness of interventions (by various health care providers in inpatient and outpatient settings) to deprescribe inappropriate PPIs in older adults was performed by Wilsdon et al. [54]. Deprescribing was defined in the article as the process of withdrawing an inappropriate medication, supervised by a healthcare professional, with the aim of managing patient medication burden and improving outcomes. Included studies reported impact of the intervention on inappropriate PPI use, and no comparators were required. Qualitative analysis of 21 studies revealed that interventions to deprescribe PPIs were effective in six studies, with the effects inconclusive (n = 11) or negative (n = 4) in the remaining studies [54].

As SUP initiated in the ICU or non-ICU settings are frequently continued inappropriately during transitions-of-care, the objective of our systematic review and meta-analysis was to estimate the impact of pharmacy-supported interventions on the proportion of patients discharged from the hospital on inappropriate AST. To our knowledge, no previous systematic reviews have quantitatively assessed the impact of pharmacist-supported interventions on AST use during hospitalization and at discharge. Differences between our systematic review and meta-analysis and the systematic review by Wilsdon et al. include 1) focusing on hospitalized patients on AST prior to arrival or newly-started on AST; 2) evaluating our outcome of interest at discharge from the ICU (to floor and then to previous living arrangements) and non-ICU settings (from floor to previous living arrangements); 3) providing a detailed analysis of pharmacy-supported interventions; and 4) requiring a comparator group.

## Methods

Preferred reporting items for systematic reviews and meta-analyses (PRISMA) guidelines were followed for reporting results [55] (S1 File) and the review was registered in the PROSPERO international registry of systematic reviews (CRD42018095347).

### Data sources and searches

The following databases were systematically searched to identify all studies which assessed the impact of pharmacist-supported interventions in addressing inappropriate AST at discharge: Ovid MEDLINE(R) and In-Process & Other Non-Indexed Citations and Daily, Embase.com, CINAHL, Web of Science, Cochrane CENTRAL (EBSCO), and ClinicalTrials.gov, from inception to October 14, 2018, with no language restrictions. Unpublished studies available in these databases (including trials and conference abstracts) were also identified and assessed for inclusion. Search strings were developed for each database, which included keywords as well as subject headings and syntax specific to each one. To exclude animals, we used the Human filter for Ovid MEDLINE recommended in the *Cochrane Handbook for Systematic Reviews of Interventions*, then we translated the filter for Embase [56]. All results were exported to EndNote. Duplicates (7,301) were identified and removed using the de-duplication method from Bramer et al. [57] for a total of 13,447 unique citations. An updated literature search using the same terms and parameters was conducted on September 12, 2019 for a total of 975 unique citations. Detailed search strategies can be found in S2 File. Reference lists of published reviews on appropriate AST prescribing and articles included for full-text assessment were searched for additional studies.

### Study selection

Randomized, controlled, observational, and non-randomized pre-post studies with a retrospective or prospective design were assessed. Each study consisted of a majority of patients ≥ 18 years of age, active pharmacy involvement by pharmacist, pharmacy students, and/or technicians (alone or a component of a multidisciplinary program), a historical or usual/standard care group, and discharge data (proportion of patients in each group discharged on inappropriate AST or a means to calculate). Studies must have included a proportion of patients receiving PPIs. Abstracts were included if they met inclusion criteria and had data describing the outcome of interest.

Studies were excluded if the majority of patients were < 18 years of age; if researchers could not determine pharmacy involvement after review of the publication or via review of authors’ titles or email verification by authors; if they were single-group studies where no historical data were provided; or if discharge data were not provided in a format that allowed for calculation of outcome of interest (or authors unable to provide clarification). Finally, potentially relevant foreign language articles were excluded once the authors confirmed there were no English versions available.

De-duplicated studies were divided into three sets and assigned to groups of two researchers each (group 1: DSF and MM; group 2: DSF and AF; group 3: DM and MD). Within each group, researchers independently performed a preliminary selection of articles based on a review of titles and abstracts followed by their full-text assessments, data extraction, and bias analysis. Finally, each group met with DSF and collectively determined inclusion and exclusion of articles, verified extracted data and reviewed bias assessments. Disagreements were resolved by consensus and after consultation with WRW.

### Data extraction and quality assessment

The authors involved in the review process used a standardized Excel documentation form to extract the following data from the studies that matched our criteria as stated above: publication year, study setting (ICU versus non-ICU), study design, observation periods, inclusion/exclusion criteria, definition of AST appropriateness/inappropriateness, pharmacy-supported interventions, sample size, mean age, gender, and proportion of patients discharged on inappropriate AST from the ICU and non-ICU settings. Authors of the primary studies were contacted at this stage if vital data were missing. Studies were evaluated using the Newcastle-Ottawa quality assessment scale [58], using the following scoring methodology: total score ≥ 7 (maximum 9) were classified as good quality studies, total score between 5–6 were classified as satisfactory, and total score less than 5 were rated as poor quality studies [58]. Only studies rated as good or satisfactory were included in the review.

### Data analysis

A meta-analysis was performed to assess the impact of pharmacy-supported interventions on the proportion of patients discharged from an institution on inappropriate AST in the ICU setting and in the non-ICU setting. The outcome was an OR and 95% CI calculated for the pharmacy-supported interventions versus historical/usual care group in each study. Outcome estimates were pooled, and random effects model was employed with assumption that the effect size will vary somewhat between studies due to differences in studied populations.

Heterogeneity was assessed by calculating Z score (Q test) and Chi-square statistic set at P < 0.1 [59]. *I*^*2*^ was calculated to quantify heterogeneity of results of studies and considered low, moderate or high if ≤25%, 26%–74% or ≥75%, respectively [59]. Where applicable and appropriate, possible sources of heterogeneity were evaluated by conducting subgroup analyses. If heterogeneity could not be explained by subgroup analyses, modified funnel plot analysis would be employed to assess heterogeneity and publication bias (further details found in S3 File). Publication bias in the subgroups was assessed via visual inspection for asymmetry of funnel plots as sub-classification of our included studies were not large enough to employ any of the recommended quantitative bias tests [60].

Meta-analyses were performed using Review Manager 5 software (Review Manager (RevMan) (Computer program), Version 5.3. Copenhagen: The Nordic Cochrane Centre, The Cochrane Collaboration, 2012). NCSS was used for the funnel plot methodology [61, 62] (NCSS 2019 Statistical software (2019). NCSS, LLC. Kayesville, Utah, USA, ncss.com/software/ncss).

## Results

### Study inclusion

**Fig 1:**
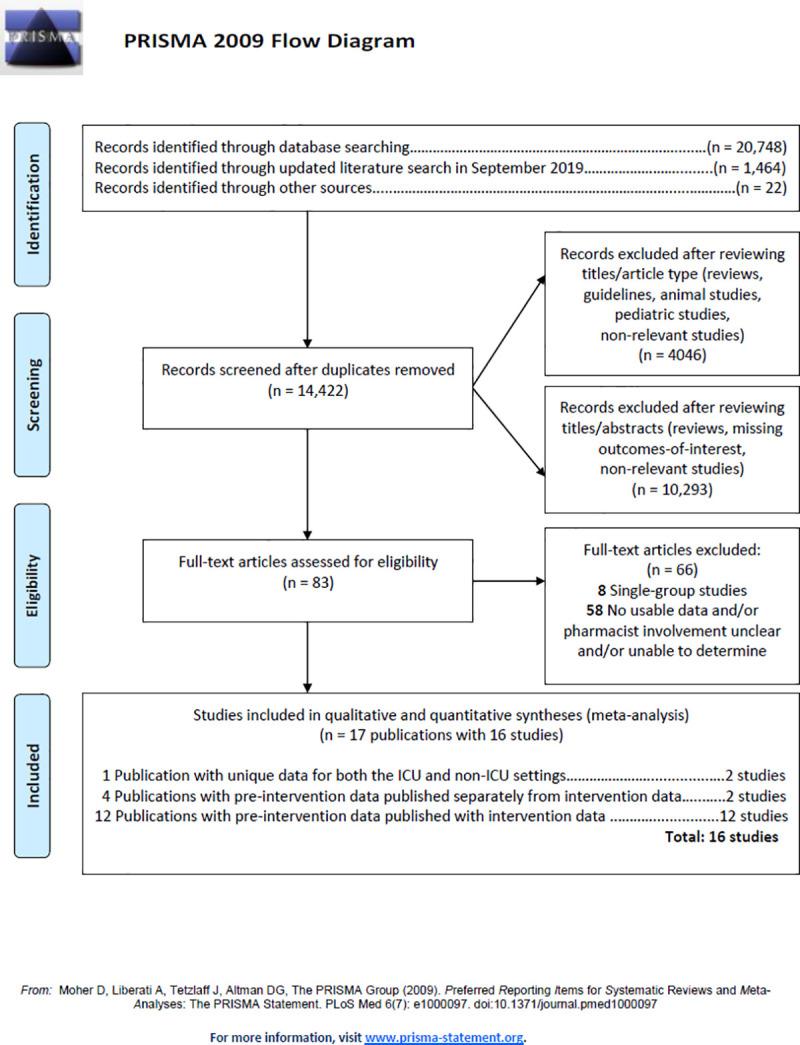
Flowchart of the literature search and study selection.

The PRISMA flowchart of study inclusion is illustrated in Fig 1 [55]. After screening and determination of eligibility of 14,422 citations, 17 articles published between 2007 and 2019 were ultimately included in the meta-analysis [63–79]. These 17 publications resulted in 16 studies since Buckley et al. [65] reported data for both the ICU and non-ICU settings in a single publication and two authors published results of pre- and post-intervention data in separate articles; in the ICU setting: Wohlt et al. (pre-implementation) [75] and Hatch et al. (post-implementation) [68]; in the non-ICU setting: Khudair et al. (pre-implementation) [71] and Khudair et al. (post-implementation) [70]. Two studies were only available in abstract form; in the ICU setting: Martz et al. [72] and in the non-ICU setting: Wu et al. [76]. All studies were conducted in the US except for studies by Khudair et al. (Qatar) [70, 71] and Van der Linden et al. (Belgium) [79]. Nine authors provided some additional data [63–68, 72, 74, 75, 78].

All studies, except for Martz et al. [72], stated that medical records were utilized for data extraction. No study reported if multiple groups were involved with data extraction and assessment and no study reported agreement ratings across data extractors’ judgement for each evaluated medical record. No study reported conducting blinded data collection or assessment.

#### Publications in the ICU setting

Characteristics of the eight retrospective publications (resulting in 7 studies) conducted in the ICU setting are summarized in Table 1 [65, 67, 68, 72–75, 77]. Buckley et al., Hammond et al., Martz et al., and Zeigler et al. considered all adults started on SUP within the ICU setting for study inclusion [65, 67, 72, 77]. Pavlov et al., Tasaka et al., and Wohlt-Hatch et al. considered all admitted patients, regardless of whether they were on AST or not, for study inclusion [68, 73–75].

**Table 1:** Characteristics of included studies conducted in the ICU setting.

Author	Data collection periods	Definition of appropriate and inappropriate AST	Intervention	Outcome(s)
Publication year				
Study design	Sample size (N)			
Inclusion criteria	Mean age			
Exclusion criteria	% Male			
**Buckley, 2015 [65]** Retrospective pre- and post-implementation study Data collected for retrospective review: • Demographics, AST indication, agent utilized, duration of use, doses administered; • Inappropriate agents, doses, and duration of therapy Inclusion Criteria: All adults (≥18 years) who received AST Exclusion Criteria: 1. On AST prior to arrival 2 On AST for GI diseases 3. Received AST in the emergency department, rehabilitation facility, or psychiatric ward Primary Outcome • Compare the mean percentage of patient-days of inappropriate SUP before and after the implementation of the clinical pharmacist-managed program	Pre-implementation: • January 2011 • N = 174 • Age: 58.3 years • Male: 51.7% Intervention implementation: October 2011 Post-implementation: • January 2012 • N = 167 • Age: 55.5 • Male: 65.9%	Major risk factors for stress-related mucosal disease GIBs: 1) mechanical ventilation; 2) coagulopathy (platelet count < 50,000 mm3, international normalized ratio >1.5, or partial thromboplastin time > 2 times control value)); 3) solid organ transplant. SUP was also indicated if ≥ 1 major risk factor plus comorbidities such as sepsis, hypotension, hepatic failure, renal dysfunction, or glucocorticoid therapy Appropriate SUP limited to ICU • Agent used, and risk factors present • No agent used, and no risk factors present Inappropriate SUP in ICU • Agent used, and no risk factors present • No agent used, and risk factors present	1. Pharmacists had prescriptive authority to initiate, modify, or discontinue SUP within the context of the defined institutional protocol using computerized provider order entry a. According to protocol, pharmacists could discontinue AST lacking an appropriate indication or, once major risk factors were resolved in ICU patients b. All AST orders from pharmacists were forwarded to physicians via electronic medical records for electronic signature authorizing orders	Number of patients discharged from the **ICU** on inappropriate AST Pre: 118/174 versus Post: 65/167 Number of patients discharged from the **hospital** on inappropriate AST Pre: 52/174 versus Post: 6/167
**Hammond, 2017 [67]** Retrospective Data collected for retrospective review: • Age, length of stay, SUP indication, AST type (H2B or PPI), proportion inappropriately initiated on AST, continued at ICU transfer, at hospital discharge • Appropriateness was assessed at time of AST initiation and at time of transfer from the ICU Inclusion Criteria: All adults (≥18 years) admitted to medical ICU and had an order for AST Exclusion Criteria: 1. Taking AST prior to admission to the ICU (either from home or from another health care setting) 2. Had a current diagnosis of GIB 3. Had a history of Zöllinger-Ellison syndrome Primary Outcome • Percentage of patients prescribed inappropriate SUP in a medical ICU before and after targeted educational interventions combined with multidisciplinary endorsement and distribution of a SUP pocket card	No intervention: • January to June 2014 • N = 101 • Age: 51.07 years • Male: Not reported Intervention period: • January to June 2015 • N = 118 • Age: 56.24 years • Male: Not reported	Appropriateness was assessed at the time of AST initiation and at the time of transfer from the ICU.Appropriate SUP: Defined in pocket card: mechanical ventilation, coagulopathy or ≥ 2 of the following: History of GI ulcer/bleeding within 1 year; severe burn (>35% of body surface are a); head or spinal cord injury; acute organ dysfunction (including acute kidney injury); liver failure with associated coagulopathy; hypoperfusion; postoperative transplantation; major surgery; multiple trauma; high daily dose of corticosteroids Inappropriate SUP: • On SUP but did not meet criteria on pocket card Patients continuing therapy when transferred to a non-ICU setting and after discharge were considered appropriately prescribed AST if: • Documentation of a new diagnosis requiring treatment such as GERD or PUD • Receiving AST prior to arrival to ICU Need to continue mechanical ventilation at skilled nursing facility • Coagulopathy with specific recommendation by a physician for continued AST	The ICU team made decisions to initiate or discontinue AST. Pharmacists did not have prescriptive authority and AST was not available to order directly from an ICU admission order set. 1. Beginning in January 2015, pharmacists provided medical residents and pulmonary/critical care fellows with an educational intervention lasting 5 minutes on guideline-recommended AST for SUP, supplied a pocket card on SUP initiation and choice of agent that had been developed by a multidisciplinary team, and answered questions related to AST for SUP 2. Pharmacists rounded with medical ICU treatment team during both study periods; however, no formal intervention of this nature was performed during the no-intervention period	Number of patients discharged from the **ICU** on inappropriate AST No intervention: 61/101 versus Intervention: 63/118 Number of patients discharged from the **hospital** on inappropriate AST No intervention: 18/101 versus Intervention: 16/118
**Martz, 2018 Abstract [72]** Retrospective quasi-experimental study with randomized sampling Data collected for retrospective review: • Proportion of patients on SUP at ICU discharge and hospital discharge Inclusion Criteria: All adults (≥18 years) admitted to medical ICU and were initiated on SUP Exclusion Criteria: Not Reported Primary Outcome • Establish a process for pharmacists to discontinue temporary medications in the ICU	Conducted during the same 3-month period in consecutive years Control group • N = 62 • Age: Not reported • Male: Not reported Intervention group • N = 57 • Age: Not reported • Male: Not reported	None provided	Control group: Medication adjustments based on standard practice Intervention group: Medications discontinued based on pharmacist-driven protocol	Number of patients discharged from **ICU** on inappropriate AST Control group: 28/62 versus Intervention group: 14/57 Number of patients discharged from the **hospital** on inappropriate AST (author provided data via email) Control group: 12/62 versus Intervention group: 2/57
**Pavlov, 2014 [73]** Retrospective Data collected for retrospective review: • Demographic (age, gender) and clinical (comorbidities; type of admission; primary diagnosis; stay; days requiring mechanical ventilation; type of respiratory failure if present; administration of AST before admission and during mechanical ventilation; discharge medications, i.e., acid blockers) Inclusion Criteria: 1. All adults (≥18 years) admitted to the medical-surgical ICU 2. Patients could be on AST prior to admission Exclusion Criteria: 1. Patients who died during hospitalization Primary outcome • If pharmacy technician-driven, physician-confirmed medication reconciliation would reduce the frequency of medication error	Pre-implementation: • February to April 2012 • N = 253 (65 patients on AST prior to admission) • Age: 65.5 years • Male: 60% Intervention implementation: After May 2012 Post-implementation: • July to September 2012 • N = 291 (113 patients on AST prior to admission) • Age: 62.4 years • Male: 51%	Pharmacy technicians completed medication reconciliation upon patient admission Appropriate SUP • Not clearly stated Inappropriate SUP • Not clearly stated Appropriate AST • GI diseases diagnosed any time during hospitalization	1. After May 2012, emergency room pharmacy technicians compiled, and recorded medications taken prior to admission, reviewed previous discharge notes and local outpatient pharmacy records. A final list of prior-to-admission medications was entered in an electronic medical record, which was reviewed/approved by the admitting attending physiciana. a. Attending physician also reviewed both pre-admission and inpatient medication lists and reviewed the reconciled final list (created by a discharging nurse) with the patient at discharge	Number of patients discharged from the **hospital** on inappropriate AST Pre-pharmacy technician period: 36/253 versus pharmacy technician period: 20/291
**Tasaka, 2014 [74]** Retrospective Data collected for retrospective review: • Demographic and ICU admission–related data • Additional collected data included date of ICU and hospital discharge, AST ordered on ICU transfer or discharge orders, and indication for AST at each transition of care. Inclusion Criteria: 1. All adults (≥18 years) admitted to medical/surgical ICU 2. Patients could be on AST prior to admission Exclusion Criteria: 1. Had active GIB, peptic ulcer disease, total gastrectomy, solid organ transplant 2. On dual antiplatelet therapy, concurrent antiplatelet and anticoagulant therapy, or nonenteric coated pancrelipase via gastric feeding tube Primary outcome • Incidence of inappropriate use of SUP in the ICU reported in inappropriate days of therapy for 100 patient-days	Pre-intervention: • Two-week period in September 2012 • N = 73 (9 patients on AST prior to admission) • Age: Not reported • Male: Not reported Intervention implementation: December 2012 Post-intervention: • Two-week period in February 2013 • N = 44 (4 patients on AST prior to admission) • Age: Not reported Male: Not reported	Assessments for appropriateness occurred daily Appropriate SUP • Indication for SUP ○ GIB in the past year ○ Mechanical ventilation ○ Coagulopathy ○ Trauma ○ Spinal cord injury ○ Severe traumatic brain injury ○ Extensive thermal injury • Met both of the following or one of the above and one as follows: ○ Severe sepsis ○ High-dose steroids • Another indication for AST (e.g., GERD or Barrett's esophagus) • Not on SUP as did not have an indication Inappropriate SUP • On SUP but not indicated • Not on SUP but indicated Unknown • On AST prior to admission without a documented indication in past medical history	A multidisciplinary team developed a bundled approach to reduce SUP overutilization 1. Institution guideline that defined SUP indications 2. Education and awareness campaign via presentation to medical residents rotating through ICU, posters, newsletter articles, emails and presentation at meetings 3. ICU pharmacists recommend changes to SUP therapy during morning rounds, in person or via text page with documentation in electronic medical record	Number of patients discharged from the **ICU** on inappropriate AST Pre-intervention period: 15/74 versus post-intervention period: 6/50 Number of patients discharged from the **hospital** on inappropriate AST (including 9 and 4 unknowns in pre- and post-intervention groups, respectively Pre-intervention period: 14/73 versus post-intervention period: 4/44
**Wohlt (pre-intervention), 2007 [75]** **Hatch (post-intervention), 2010 [68]** RetrospectiveData collected for retrospective review: • Demographic and ICU-related data including age, sex, length of stay, admission diagnosis, prior AST use, ICU status when SUP was prescribed, and the associated indication, SUP including the drug name, dose, and continuation beyond transfer from the ICU to general care floors, discharge medications Inclusion Criteria: 1. All adults (≥18 years) admitted to medical/surgical ICU 2. Patients could be on AST prior to admission Exclusion Criteria: 1. Had a variceal bleed, GIB, or Zöllinger-Ellison syndrome 2. Death prior to hospital discharge Primary outcome •Proportion of patients discharged from the hospital on inappropriate AST	Pre-intervention period: • July 1, 2005 to September 30, 2005 • N = 394 (101 patients on AST prior to admission) • Age: 54 years • Male: 58% Intervention implementation: October 2006 Post-intervention period: • November 1, 2006 to January 31, 2007 • N = 356 (158 patients on AST prior to admission) • Age: 55 years • Male: 59%	Pharmacists evaluated indications and appropriateness of drug therapy on a daily basis and at transitions-of-care Appropriate SUP defined in institution’s prescribing guidelines • Mechanical ventilation • Coagulopathy • Burns • Organ transplant during current hospitalization • Spinal cord injury • Hepatectomy, failure • Glasgow Coma Score ≤ 10 • Inability to obey simple commands • Multiple trauma • GI ulcers in past year • ≥ 2 of the following ○ Sepsis ○ ICU stay ≥ 1 week ○ Occult bleeding lasting ≥ 6 days ○ High-dose steroids Appropriate AST at discharge • Mechanically ventilated and discharged to long term care facility • Diagnosed with GERD/peptic ulcer disease • Developed a GIB during hospitalization • History of GI ulcer/bleed within past year • Prescriber-indicated reason On AST prior to arrival Inappropriate AST upon discharge • On AST without an indication	1. Email of SUP guidelines to attending physicians and residents 2. Distribution of pocket card with hospital SUP guidelines during rounds and to pharmacists to aid with medication reconciliation at care transfer	Number of patients discharged from the **ICU** on inappropriate AST Pre-intervention period: 189/394 versus post-intervention period: 84/356 Number of patients discharged from the **hospital** on inappropriate AST Pre-intervention period: 96/394 versus post-intervention period: 31/356
**Zeigler, 2008 [77]** Retrospective Data collected for retrospective review: • Demographic and ICU-related data • Medications (SUP, NSAIDs, antiplatelets) Inclusion Criteria: All adults (≥18 years) admitted to surgical/medical ICU and received SUP Exclusion Criteria: 1. On AST prior to arrival 2. Treated for acute GIB 3. Died while in the hospital Primary outcome • Effect of medication reconciliation on incidence of prolonged (or inappropriate) SUP across the continuum of care from ICU to hospital discharge	Pre-intervention: • September 2005 • N = 53 • Age: Not reported • Male: Not reported Intervention implementation: March 2006 Post-implementation: • September 2006 • N = 61 • Age: Not reported • Male: Not reported	Appropriate SUP • Mechanical ventilation • Coagulopathy • ≥ 2 of the following: ○ Severe head trauma ○ Acute spinal cord injury ○ Burns ○ Sepsis ○ Renal insufficiency ○ Hepatic failure ○ Trauma ○ Partial hepatectomy ○ Hepatic/renal transplant ○ GIB within 1 year Inappropriate SUP • SUP continued in the absence of ≥1 major risk factor or 2 minor risk factors as described in institutional SUP guidelines	1. Education of all clinical staff on the medication reconciliation process (not on SUP) a. Medication reconciliation process where pharmacists and nurses obtained medication histories b. Physicians reviewed medication profiles during care transfer/discharge c. Institutional guidelines for SUP were available and in place for both pre- and post-intervention periods	Number of patients discharged from the **ICU** on inappropriate AST Pre-intervention period: 45/53 versus post-intervention period: 48/61 Number of patients discharged from the **hospital** on inappropriate AST Pre-intervention period: 8/53 versus post-intervention period: 15/61

Patients who died during their admissions or were not discharged at the time of data extraction were excluded from all studies. Buckley et al., Hammond et al., Wohlt-Hatch et al., Zeigler et al. and Tasaka et al. excluded patients with GIB [65, 67, 68, 74, 75, 77]; Buckley et al. and Tasaka et al. excluded patients with peptic ulcer disease [65, 74]; Hammond et al. and Wohlt-Hatch et al. excluded patients with Zollinger-Ellison Syndrome [67, 68, 75]; Buckley et al. excluded patients with GERD [65]; Tasaka et al. excluded patients who had a total gastrectomy, solid organ transplant, and were receiving dual antiplatelet therapy, concurrent antiplatelet and anticoagulation therapy, or non-enteric-coated pancrelipase via gastric feeding tube [74]. Pavlov et al. and Martz et al. did not provide any specific exclusion criteria [72, 73].

Sample size calculations were conducted by Buckley et al., Hammond et al. and Zeigler et al., and all studies included the sample sizes required to determine statistical significance of their respective primary outcomes [65, 67, 77]. Primary outcomes for each study are listed on Table 1. The proportion of patients discharged from the hospital on inappropriate AST was the primary outcome for the Wohlt-Hatch et al. and Zeigler et al. studies [68, 75, 77]; this outcome was secondary for the Hammond et al. and Tasaka et al. studies [67, 74]. This outcome was not listed in the Methods section of the studies by Buckley et al., Martz et al., and Pavlov et al., but the data were provided in their Results section or via email communication (Martz et al.) [65, 72, 73].

#### Care during pre-intervention period

In Buckley et al., during the pre-intervention period, pharmacists did not have prescriptive authority [65]. Hammond et al. reported that a clinical pharmacist rounded with the medical ICU treatment team during both no-intervention and intervention study periods, but that no formal pharmacist-led interventions (education and pocket cards) was performed in the no-intervention period [67]. Wohlt-Hatch et al. reported that during the pre-intervention period, attending physicians were encouraged to withhold SUP in all patients except those with head injuries, burns over more than 30% of their body-surface area, those receiving organ transplants, those with an endoscopic or radiographic diagnosis of peptic ulcer or gastritis in the preceding 6 weeks, or patients with upper GIB 3 days to 6 weeks before admission [68, 75]. Martz et al. reported a control group and an intervention group; the control group had medications discontinued through standard practice while in the intervention group, medications were discontinued through a pharmacist-driven protocol [72]. In Pavlov et al., during the pre-intervention period, pharmacy was not involved in medication reconciliation upon admission; only the admitting prescribers completed medication reconciliation upon admission [73]. Zeigler et al. reported that institutional SUP guidelines were available and in place for both the pre- and post-medication reconciliation period [77]. Buckley et al. [65] and Tasaka et al. [74] did not explain if any type of interventions (i.e., standard practice) was implemented during the pre-intervention study periods, however various SUP publications [5, 80–82] cited by the authors of the included studies were published prior to study implementation, therefore, standard practice or usual care may have occurred during pre-implementation periods.

#### Observation periods

Observation periods varied between studies. Tasaka et al. collected two-weeks each of pre and post-intervention data [74]; Buckley et al. and Zeigler et al. collected one month of pre-post data [65, 77]; Wohlt-Hatch et al., Martz et al., and Pavlov et al. collected three months of pre-post data [68, 72, 73, 75] and Hammond et al. [67] collected six months of pre-post data. In studies that provided data [65, 67, 68, 73], patients’ mean age ranged from 51 to 65.5 years, and the percentage of males ranged from 51.7% to 65.9%.

#### Publications in the non-ICU setting

Characteristics of 10 publications (resulting in 9 studies) conducted in the non-ICU settings are summarized in Table 2 [63–66, 69–71, 76, 78, 79]. Seven publications were within an internal medicine/general ward [63–66, 70, 71, 76] and one each within a Geriatric Evaluation and Management unit at the Veterans Affairs Medical Center [69], acute geriatric ward [79], and hematology/oncology inpatient unit [78]. Four studies were retrospective (Agee et al., Belfield et al., Buckley et al., Ziegler et al.) with a pre-post intervention design [63–65, 78]; these studies did not report if patients in the pre-intervention groups received any interventions (i.e., usual care) [63–65, 78]. Hughes et al. was retrospective and pre-post, but the pre-intervention group was a control group; interdisciplinary team rounds occurred once weekly throughout both study arms (control versus intervention), but a clinical pharmacist was present during rounds in the intervention arm [69].

**Table 2:** Characteristics of included studies conducted in the non-ICU setting.

Author Publication year Study design Inclusion criteria Exclusion criteria	Data collection periods Sample size (N) Mean age % Male	Definition of appropriate and inappropriate AST	Intervention	Outcome(s)
**Agee, 2015 [63]** Retrospective Inclusion Criteria: All adults (≥18 years) admitted to medical floor Exclusion Criteria: 1. Currently in ICU 2. Had diagnosis of GERD, *H*. *pylori* infection, peptic ulcer disease, dyspepsia, Zöllinger-Ellison syndrome, or Barrett’s esophagus Primary outcome: • Assess the impact of education for appropriate SUP prescribing by Family Medicine Resident Physicians	Pre-intervention: • September through November 2011• N = 220 (12 patients on AST prior to admission) • Age: not reported • Male: not reportedIntervention implementation:January 2012Post-intervention: • February through April 2012N = 193 (16 patients on AST prior to admission) • Age: Not reported • Male: Not reported Intervention implementation: January 2012 Post-intervention: •February through April 2012 •N = 193 (16 patients on AST prior to admission) •Age: Not reported •Male: Not reported	Appropriate prescribing and discontinuation of AST upon discharge were evaluated utilizing the 1999 ASHP Gastrointestinal Stress Ulcer Prophylaxis guidelines [5] Appropriate AST • Defined as per SUP guidelines and literature review • Mechanical ventilation • Coagulopathy • GIB within past year • Glasgow Coma Score ≤10 • Burns • Partial hepatectomy • Multiple trauma • Transplantation • Spinal cord injury • Hepatic failure • ≥ 2 of the following ○ Sepsis ○ ICU stay > 1 week ○ Occult bleeding lasting ≥ 6 days ○ High-dose steroids Inappropriate AST • Not clearly stated	1. Educational seminar describing appropriate SUP indications (for ICU setting only), associated risks and costs of AST to residents 2. Distribution of handout containing a complete literature review, and pocket card outlining appropriate indications	Number of patients discharged from the hospital on inappropriate AST Pre-intervention period: 36/220 versus post-intervention period: 15/193
**Belfield, 2017 [64]** Retrospective Data collected for retrospective review: • Demographics and medication use • Pharmacy interventions • Hospitalist interventions Inclusion Criteria: All adults (≥18 years) admitted to medical floor Exclusion Criteria: On AST prior to admission Primary outcome • Comparison of the proportion of inpatient days with inappropriate AST	Pre-intervention: • October 2015 • N = 61 • Age: 65.6 years • Male: 60% Intervention: • March 2016 • N = 81 • Age: 68.6 years • Male: 55%	Appropriate AST • New or recent GIB, GERD, peptic ulcer disease, dyspepsia • Dual antiplatelet therapy Inappropriate AST • Not clearly stated	1. Pharmacists reviewed AST orders from daily census list and contacted hospitalists for clarification as necessary 2. Pharmacists encouraged hospitalists to discontinue AST based on institution’s SUP guidelines 3. Weekly education, including a literature review of stress ulceration in non-critically ill patients and appropriate AST indications 4. Biweekly updates on intervention to hospitalists	Number of patients discharged from the hospital on inappropriate AST Pre-intervention period: 13/61 versus post-intervention period: 3/81
**Buckley, 2015 [65]** Retrospective Data collected for retrospective review: • Demographics, AST indication, agent utilized, duration of use, doses administered; • Inappropriate agents, doses, and duration of therapy Inclusion Criteria: All adults (≥18 years) who received either AST on the general ward Exclusion Criteria: 1. On AST prior to arrival 2. On AST for GI diseases 3. Received AST in the emergency department, rehabilitation facility, or psychiatric ward Primary Outcome • Compare the mean percentage of patient-days of inappropriate SUP before and after the implementation of the clinical pharmacist-managed program	Pre-implementation: • January 2011 • N = 589 • Age: 57.2 years • Male: 45.2% Implementation of pharmacist-managed program: October 2011 Post-implementation phase: • January 2012 • N = 204 • Age: 58.4 years • Male: 50%	Inappropriate AST • Administered therapy without an indication	1. The pharmacy program included prescriptive authority for AST under a collaborative practice agreement enabling the pharmacist to discontinue therapy in patients without an indication and not taking as a home medication prior to hospital admission	Number of patients discharged from the hospital on inappropriate AST Pre: 213/589 versus Post: 11/204
**Carey, 2011 [66]** Prospective, observational cohort study Data collected for retrospective review: • Type of AST, diagnoses, length of stay Patients were selected for medication review from team’s daily list of new admissions. Every third patient prescribed at least one dose of an AST was selected for review Inclusion Criteria: All adults (≥18 years) admitted to internal medicine teaching services from home/ICU and received AST Exclusion Criteria: Not reported Primary outcome • Proportion of patients on inappropriate AST	No pharmacy students: • June through September 2008 • N = 192 (110 patients on AST prior to admission) • Age: Not reported • Male: Not reportedPharmacy students: • October 2008 through January 2009 • N = 197 (116 patients on AST prior to admission) • Age: Not reported •Male: Not reported Pharmacy students: • October 2008 through January 2009 • N = 197 (116 patients on AST prior to admission) • Age: Not reported • Male: Not reported	Appropriate AST • Home AST on admission • History of GERD, peptic ulcer disease, erosive esophagitis, gastritis, dyspepsia • Acute or suspected upper GIB, or GIB during previous 3 months • On ≥ 2 medications: NSAIDs, aspirin, clopidogrel, warfarin, heparin, low molecular weight heparin, steroids Inappropriate AST • Above conditions not met	1. Pharmacy students completing advanced pharmacy practice experiences evaluated medication profiles daily for AST and participated in patient rounds 5 days per week a. Students received training on appropriate AST, impact of unnecessary AST, strategies for interventions (ex., questioning AST indication during rounds, presenting patient-specific implications of inappropriate AST) b. Pharmacist preceptors met with students following rounds to review recommendations	Number of patients discharged from the hospital on inappropriate AST No pharmacy students: 18/192 versus Pharmacy students: 12/197
**Hughes, 2011 [69]** Retrospective Data collected for retrospective review: • AST use prior to discharge, diagnoses Inclusion Criteria: All adults (≥18 years) admitted to Geriatric Evaluation and Management unit and had a minimum length of stay of 7 days Exclusion Criteria: Admissions shorter than 7 days Primary outcome • Number of patients receiving AST at discharge without an appropriate indication	Control arm: • July 2006 through April 2007 • N = 117 • Age: Not reported • Male: Not reported Intervention arm: • May 2007 through February 2008 • N = 117 • Age: Not reported • Male: Not reported	Appropriate AST • GERD, peptic ulcer disease, hiatal hernia, esophagitis, erosive esophagitis, gastritis, dyspepsia, Barrett’s esophagus, Zöllinger-Ellison syndrome • GIB Inappropriate AST • Above conditions not met • On SUP	1. Pharmacists were present during weekly interdisciplinary rounds to make recommendations on AST prescribing	Number of patients discharged from the hospital on inappropriate AST No pharmacists: 54/117 versus pharmacists: 28/117
**Khudair (pre-intervention),** **2009 [71]** **Khudair (post-intervention), 2011 [70]** Retrospective followed by prospective evaluation Data collected for retrospective review: • Gender, age, current diagnosis, length of stay, admission through ICU, type of AST used together with route and total daily dose, AST usage before admission, possible risk factors, and indication for use. Inclusion Criteria: All patients (≥ 14 years) admitted to general medicine wards Exclusion Criteria: Not reported Primary outcome • Evaluate the impact of a multi-approach strategy implemented after a baseline audit done in 2007, to improve the prescribing and usage of ASM in medical inpatients	Pre-intervention: • May through June 2007 • N = 190 (48 patients on AST prior to admission) • Age: 51 years • Male: 81% Intervention period: during 2008, but not clearly stated Post-intervention: • May through June 2009 • N = 194 (74 patients on AST prior to admission) • Age: 52.6 years • Male: 66%	Medical records were reviewed and monitored for ASM usage at 3 stages: during hospital stay, upon discharge and at follow-up visit in the outpatient clinic. For purposes of our meta-analysis, AST use at follow-up was not evaluated. Appropriate AST • GERD, peptic ulcer disease, erosive esophagitis, *H*. *pylori* eradication, Zöllinger-Ellison syndrome • Treatment and prophylaxis of NSAID-induced ulcers • SUP • Liver cirrhosis • Organ transplantation • Steroids plus NSAIDs Inappropriate AST • Above conditions not met	1. Report of AST usage patterns to medical and pharmacy staff at institution 2. Development and implementation of evidence-based AST usage guideline 3. Distribution of flyer with algorithm to reassess need for AST at various stages of care 4. Pharmacists rounded with clinical teams 5 days/week	Number of patients discharged from the hospital on inappropriate AST Pre-intervention 70/190 versus post-intervention 26/194
**Van der Linden, 2017 [79]** Prospective, controlled, 11-month study Inclusion Criteria:Adults (≥18 years) admitted to acute geriatric wards Exclusion Criteria: 1. Admitted for end-of-life care 2. Did not take any drugs 3. Not discharged back to their home or nursing home Primary outcome • Evaluate the effect of a pharmacist intervention, consisting of the application of the Rationalization of home medication by an Adjusted STOPP in older Patients (RASP) list and a pharmacist-led medication review on polypharmacy, the quality of prescribing, and clinical outcome in geriatric inpatients	Control arm: • N = 81 • Age: 84.5 years • Male: 44% Intervention arm: • N = 91 • Age: 84.5 years • Male: 52%	Appropriate AST • Not clearly stated Inappropriate AST • Not clearly stated, but, the Rationalization of home medication by an Adjusted STOPP in older Patients (RASP) list was applied ○ Prolonged use of PPIs and H_2_RAs in peptic ulcer disease	1. Pharmacists performed medication reconciliation with a subsequent two-stage medication review a. In the first step, the RASP list was applied b. In the second step, an additional comprehensive medication review by CPs was conducted (eg., to determine prolonged use of PPIs) 2. Pharmacists attended ward rounds 3. Daily recommendations were reported to treating physician 4. RASP list applied to medication list prior to discharge	Number of patients discharged from the hospital on inappropriate AST Control arm: 32/81 versus Intervention arm: 5/91
**Wu, 2015 Abstract [76]** Prospective, quality improvement study in a general medicine ward Inclusion Criteria: Not reported Exclusion Criteria: Not reported Primary outcome • Percentage of patients that remain on a PPI without indication upon discharge	Control ward: Same 4 weeks as in intervention ward • N = 18 • Age: Not reported • Male: Not reported Intervention ward: As of April 14^th^, 2015, the intervention was in place for 4 weeks • N = 21 • Age: Not reported • Male: Not reported	Appropriate AST • Evidence-based indication Inappropriate AST • Not clearly stated	1. Pharmacist prospectively reviewed newly admitted patients during medication reconciliation and provided a recommendation to “deprescribe” PPIs without an evidenced-based indication	Number of patients discharged from the hospital on inappropriate AST Control 10/18 versus Intervention 0/21
**Ziegler, 2019 [78]** Retrospective study in 3 inpatient hematology-oncology units Data collected for retrospective review: • PPI indication (determined by review of clinical documentation), AST prescription prior to admission, symptomatic or endoscopic evidence of GIB, and PPI continuation on discharge. Inclusion Criteria: All adults (≥ 18 years) admitted to one of three units Exclusion Criteria: Not reported Primary outcome • Rate of PPI use measured in days of therapy	Pre-implementation: August 12, 2017 to November 12^th^, 2017 • N = 155 • Age: Not reported • Male: Not reported Intervention implementation: November 13^th^, 2017 to February 13^th^, 2018 • N = 126 • Age: Not reported • Male: Not reported	Appropriate AST • On PPIs for GI symptoms and no improvement on H2B; should be stopped on discharge if no improvement Inappropriate AST • An order for PPI when there was either no documented clinical indication or if the indication was listed as “prophylaxis”	1. Institutional guideline developed via multidisciplinary collaboration a. Pharmacist disseminated electronically to house staff and advanced practice providers b. Posted on institutional electronic repository 2. Pharmacist provided in-person education in the initial month after guideline introduction for non-house staff teams with a 30-minute session focused on reviewing evidence supporting guideline and recommended strategies for AST 3. Pharmacist provided twice-monthly in-person orientations for house staff providers 4. Pharmacist rounded in units and reinforced guideline as needed	Number of patients discharged from the hospital on inappropriate AST Pre-implementation 43/155 versus Intervention 54/126

Four studies employed a prospective or partially prospective design. Khudair et al. retrospectively collected data for the pre-intervention group [71], but data for the post-intervention group were evaluated prospectively [70]. The remaining three studies employed a prospective controlled design. In Carey et al., a fourth-year pharmacy student was a member of teaching teams and attended rounds; the internal medicine teams without pharmacy students served as the control group for this study; of note, staff members at the institution were required to perform medication reconciliation using the electronic medical record of all patients whereas pharmacy students were not required to participate [66]. In Van der Linden et al., geriatricians in the control group were not informed about the study design. If potentially life-threatening drug errors were observed, this was communicated to the treating physician as part of medication reconciliation services provided in both groups [79]. In Wu et al., a ward without the pharmacist served as the control [76].

All studies reviewed the medical records of all patients admitted to their respective non-ICU settings. In Van der Linden et al., patient allocation to the intervention arm versus the control arm was based on consecutive admissions to one control and two intervention wards [79]. Belfield et al. and Buckley et al. considered all patients who were initially started on AST while on the medical floor [64, 65]. The remaining studies considered all admitted patients, regardless of whether they were on AST or not, for study inclusion.

Buckley et al. and Agee et al. excluded patients with GERD and peptic ulcer disease [63, 65]. Agee et al. also excluded patients with dyspepsia, Barrett's esophagus, *H*. *pylori* infection, and Zollinger-Ellison Syndrome [63]. Van der Linden et al. excluded patients who were admitted for end-of-life care, were not discharged back to a nursing home or previous living arrangements, or who did not take any drugs [79]. No other studies provided specific exclusion criteria.

Sample size calculations were conducted by Buckley et al., Carey et al., Hughes et al. and Van der Linden et al. and all included the sample sizes required to determine statistical significance of their primary outcomes [65, 66, 69, 79]. Primary outcomes for each study are listed on Table 2. The proportion of patients discharged from the hospital on inappropriate AST was the primary outcome for two studies: Hughes et al. (examined whether pharmacist’s presence and recommendations during interdisciplinary team rounds influenced the rate of inappropriately prescribed AST at discharge) and Wu et al. (percentage of patients that remain on a PPI without indication upon discharge) [69, 76]. The proportion of patients discharged on inappropriate AST was a secondary outcome in Agee et al., Belfield et al., Carey et al., Khudair et al., Van der Linden et al. and Ziegler et al. [63, 64, 66, 70, 71, 78, 79].

Observation periods varied between studies. In the retrospective studies, Belfield et al. and Buckley et al. collected one-month each of pre and post data [64, 65]; Agee et al. and Ziegler et al. collected three months of pre-post data [63, 78]; Hughes et al. collected 10 months of pre-post data [69]. In the prospective studies, Wu et al. collected one-month of data from the intervention and from the control ward [76]; Khudair et al. collected two months of pre-post data (post-intervention data evaluated prospectively) [70, 71]; Carey et al. collected three months of data during the no-pharmacy-student period versus pharmacy-student period [66] and Van der Linden et al. collected eleven months of data for the control group (RASP list applied retrospectively) versus intervention group (RASP list applied by pharmacist prospectively) [79]. In studies that provided data [64, 65, 70, 71, 79], patients’ mean age ranged from 51 to 84.5 years and percentage of males ranged from 44% to 81%. In Van der Linden et al., the average age of patients was 84.5 years [79].

### AST appropriateness in the ICU setting

In the ICU setting, guidance on whether to add or not add SUP to prevent GIBs are found in several peer-reviewed publications [5–7, 83–86]. Most studies reported using institutional guidelines developed via consensus of several medical and surgical hospital committees and/or published the guideline/algorithm used to guide appropriate prescribing [65, 67, 68, 74, 77]. Martz et al. reported using a pharmacist-driven medication discontinuation protocol [72] and Pavlov et al. did not report use of a specific SUP guideline or protocol [73].

#### Assessment of AST usage prior to arrival

Four publications included patients on AST prior to arrival [68, 73–75]. In Wohlt-Hatch et al., patients on AST prior to arrival were considered on AST appropriately and only inpatient SUP prescribing was assessed for appropriateness [68, 75]. Tasaka et al. assessed and provided the data on AST appropriateness for all included patients [74]. Pavlov et al. explained that patients not on AST prior to admission but were on AST on discharged and those who had received them prior to admission but were discharged without them (for no clear reason) were deemed discordant and their charts were reviewed in detail [73]. The four remaining studies included only those newly initiated on SUP during hospitalization [65, 67, 72, 77].

#### SUP handling during ICU admission

Appropriate SUP was defined as a patient with any of the following: mechanical ventilation (6 studies) [65, 67, 68, 73–75, 77]; coagulopathy (5 studies) [65, 67, 68, 74, 75, 77]; transplant (4 studies) [65, 67, 68, 75, 77]; or ≥ 2 of these risk factors: GIB history or a bleed within the previous year (4 studies) [67, 68, 74, 75, 77]; severe burns (4 studies) [67, 68, 74, 75, 77]; Glasgow Coma Scale ≥ 10 (4 studies) [67, 68, 74, 75, 77]; spinal injury (3 studies) [68, 74, 75, 77]; high daily dose of steroids (3 studies) [67, 68, 74, 75]; liver (hepatic failure or hepatectomy) (3 studies) [67, 68, 75, 77]; multiple trauma (4 studies) [67, 68, 74, 75, 77]; and septic shock/severe sepsis (3 studies) [68, 74, 75, 77].

Additional risk factors warranting SUP by single studies included hypoperfusion [67], acute organ dysfunction (including kidney injury) [67], major surgery [67], ICU stay > 1 week [68, 75], occult bleeding ≥ 6 days [68, 75], and renal insufficiency [77]. Pavlov et al. defined appropriateness as being on AST any time during hospitalization due to peptic ulcer disease or a new GERD diagnosis [73].

Inappropriate SUP was defined as follows: 1) presence of an agent, but patient had no risk factors (4 studies) [65, 68, 74, 75, 77] and/or 2) no agent was used and patient had risk factors (3 studies) [65, 74, 77]. Tasaka et al. [74] categorized patients on AST prior to arrival but without an indication in the past medical history as “unknown”. However, to be conservative, we considered these patients to be on inappropriate AST upon discharge.

#### Timing of interventions

Interventions (in real-time) occurred at various timepoints. In Pavlov et al., comprehensive medication reconciliation by pharmacy technicians occurred upon admission [73]. However, it was the physician who reviewed the final discharge medication list [73]. In Buckley et al., pharmacists had prescriptive authority to make adjustments to medications using a defined institutional protocol [65]. Prescriptive authority was available to pharmacists in the ICU and non-ICU settings [65].

In three studies, pharmacists completed ICU rounding [67, 68, 74, 75]. Martz et al. employed a protocol to discontinue SUP when it was no longer needed, but it is unclear if this took place during rounding or via electronic communication [72]. Additionally, Martz et al. reported that physicians were responsible for discharge medications (email communication) [72]. In Tasaka et al., it was unclear if pharmacy staff were available to make any patient-specific interventions regarding AST during each patient’s discharge [74].

Wohlt-Hatch et al. reported that pharmacists conducted medication reconciliation at all points of care, including evaluation of the discharge medication list [68, 75] whereas Hammond et al. reported that pharmacists did not provide medication reconciliation upon discharge at their institution [67]. Zeigler et al. educated all clinical staff on the medication reconciliation process (which could be completed at all timepoints), however, direct, targeted pharmacy-driven interventions were not reported [77]. Additionally, it was the physicians who reviewed medication profiles at care transfer/discharge [77].

#### Retrospective review of collected data

All studies provided data on the proportion of patients discharged on inappropriate AST from the hospital; therefore, evaluation of the data occurred for this timepoint. Evaluation of data also occurred upon ICU discharge by Buckley et al., Hammond et al., Wohlt-Hatch et al., Martz et al., Tasaka et al., and Zeigler et al. [65, 67, 68, 72, 74, 75, 77].

### AST appropriateness in the non-ICU setting

To guide appropriate AST prescribing in the non-ICU setting, most studies used a combination of published and/or institutional guidelines, FDA-approved indications for agents used for AST and/or used relevant data from literature reviews. Wu et al. did not clearly state in their abstract what specific guideline or protocol was used to determine appropriateness [76].

While SUP use is discouraged in the non-ICU setting, SUP guidelines were used to assess prescribing as some patients may still require prophylaxis [63, 70]. Agee et al. [63] used the 1999 ASHP Gastrointestinal Stress Ulcer Prophylaxis guidelines [5] to determine appropriate prescribing and discontinuation of AST. Since patients with GERD, *H*. *pylori* infection, peptic ulcer disease, dyspepsia, current GIB, Zollinger-Ellison Syndrome, or Barrett’s esophagus were excluded from this non-ICU study [63], it may be that patients assessed for appropriate AST use originated from the ICU (where the 1999 ASHP guidelines are more applicable). If patients no longer met the criteria for SUP, then addressing/discontinuing therapy may have been the purpose for using SUP guidelines.

Belfield et al. [64] used the American College of Cardiology Foundation, the American College of Gastroenterology (ACG), and the American Heart Association Expert Consensus Document on the Concomitant Use of Proton Pump Inhibitors and Thienopyridines and the ACG Guidelines for Prevention of NSAID-Related Ulcer Complications [9] to determine appropriate AST use in patients on antiplatelet and/or NSAID therapies. AST ordered for patients with a new or recent GIB, GERD, peptic ulcer disease, or dyspepsia was considered appropriate [64].

Carey et al. [66] considered AST appropriate for all patients if ≥1 of the following indications could be identified in the patient’s record: history of GERD, acute or suspected upper GIB or bleeding during the previous 3 months, erosive esophagitis or gastritis, dyspepsia, peptic ulcer disease, post-bariatric surgery, or the administration of ≥2 of the following medications: NSAIDs, aspirin, clopidogrel, warfarin, heparin or low molecular weight heparin for therapeutic anticoagulation, or corticosteroids. These criteria for AST appropriateness were also applied to patients transferred into the internal medicine service from the ICU [66].

Hughes et al. [69] used a combination of indications from previous studies [87–90] that examined AST appropriateness. GERD, hiatal hernia, esophagitis, erosive esophagitis, gastritis, dyspepsia, Barrett's esophagus, acid reflux, peptic, gastric or duodenal ulcer, NSAID-induced ulcer, H. *pylori* infection, Zollinger-Ellison Syndrome, or any GIB were considered appropriate use of AST [69].

Khudair et al. [70, 71] reported the following approved/justified indications for AST usage in patients on appropriate AST: NSAID and aspirin ulcer prophylaxis (high risk), dyspepsia, upper GIB, hepatic failure (cirrhotic), GERD, erosive esophagitis, H. *pylori* eradication regimen, renal/hepatic transplant, SUP according to 1999 ASHP criteria [5], prophylaxis of acid aspiration, and gastric/duodenal ulcer.

Van der Linden et al. [79] used their own validated RASP list, an iteration of the Screening Tool of Older Persons’ potentially inappropriate Prescriptions (STOPP) criteria [91, 92]. The list was developed to improve capture rate of potentially inappropriate medications compared with available tools in geriatric inpatients [79]. Appropriate indications for AST included (but might not have been limited to) documented refractory symptomatic GERD, microbleeds (due to angiodysplasias in the GIT), or being on dual antiplatelet therapy (email communication). The list was applied to patient medications upon admission and at discharge for those in the intervention arm (email communication) [79].

Ziegler et al. [78] developed a guideline via multiple disciplinary collaboration to reduce rates of C. *difficile* infection in their hematology-oncology units. This guideline provided a pathway to steer providers into prescribing AST only if clinically indicated.

Inappropriate AST was defined as not meeting conditions (indications) for appropriate AST as described above for Agee et al., Belfield et al., Carey et al., and Khudair et al. [63, 64, 66, 70, 71]. Buckley et al. stated that SUP use in non-ICU patients was considered inappropriate [65]. Hughes et al. identified patients who received SUP as receiving inappropriate therapy as the setting was a Geriatric Evaluation and Management unit for rehabilitation of medically-stable patients [69]. Van der Linden et al. [79] applied the RASP list, which recommends prolonged used of AST should be further investigated [92]. In Ziegler et al., inappropriate AST was an order for a PPI when there was either no documented clinical indication (i.e. no history of GERD or gastritis), or if the indication was listed as "GI prophylaxis" (email communication) [78]. Inappropriate AST was not clearly defined in three studies (Agee et al., Belfield et al. and Wu et al.) [63, 64, 76].

#### AST handling prior to arrival

Six studies included patients on AST prior to arrival and appropriateness was assessed during their stay in the hospital (Agee et al., Hughes et al., Khudair et al., Van der Linden et al., Wu et al., Ziegler et al.) [63, 69–71, 76, 78, 79]. Carey et al. considered AST prior to arrival as appropriate therefore only inpatient prescribing was assessed for appropriateness [66]. Only two studies exclusively included patients who were newly initiated on AST while in the hospital (Buckley et al. and Belfield et al.) [64, 65].

#### Timing of interventions

Targeted pharmacy-driven interventions occurred in real-time during medication reconciliation upon admission in two studies (Wu et al. and Van der Linden et al.) [76, 79]. During hospitalization, Belfield et al. encouraged pharmacists to contact hospitalists to clarify/discontinue AST [64]; in Buckley et al., pharmacists had prescriptive authority [65]; in five studies a pharmacy member (pharmacy students or pharmacists) made recommendations during rounds (Carey et al., Hughes et al., Khudair et al., Van der Linden et al. and Ziegler et al.) [66, 69, 70, 78, 79]. In Van der Linden et al., the RASP list was also applied to patients in the intervention arm at discharge [79]. Agee et al. provided an educational seminar about appropriate SUP indications to medical residents, but it was not reported if pharmacists made any patient-specific recommendations either in-person or electronically [63]. All studies provided data on the proportion of patients discharged on inappropriate AST from the hospital; therefore, evaluation of the data occurred for this timepoint.

### Pharmacy-supported Intervention characteristics

Tables 1 and 2 summarize pharmacy-supported interventions for each study; S4 File further abbreviates interventions for easy comparison. Most studies followed a multi-approach strategy.

#### Guideline development and dissemination

Five studies described active pharmacist participation as part of a multidisciplinary team in guideline development to address appropriate AST prescribing [64, 67, 70, 74, 78]. Belfield et al. reported the formulation of an institutional stress-related mucosal disease prophylaxis guideline [64]. Hammond et al. supplied a pocket card on SUP initiation and choice of agent that had been developed by a multidisciplinary team [67]. Khudair et al. reported development and implementation of an evidence-based AST-usage guideline [70]. Tasaka et al. provided a detailed SUP guideline formulated by the team after extensive review of the pertinent medical literature [74]. Ziegler et al. implemented a guideline for appropriate PPI use in hematology-oncology units [78].

Agee et al. distributed a handout, containing a complete literature review and a laminated pocket card outlining appropriate SUP indications (1999 ASHP guidelines) [63]. Hatch et al. distributed previously-developed guidelines [68]; Zeigler et al. reported that SUP guidelines were already in place at their institution (prior to medication reconciliation implementation) [77]. While guideline dissemination was not clearly stated by Buckley et al., Carey et al., Hughes et al., Pavlov et al., Van der Linden et al., Martz et al. and Wu et al., most reported using previously-published articles describing the appropriate use of AST in combination with consensus through hospital committees, to develop institution-specific guidelines [65, 66, 69, 72, 73, 76, 79].

#### Education and awareness campaigns

Eight studies described pharmacists providing education to health care providers (including pharmacy staff) about appropriate AST prescribing via in-person, electronically and/or as printed materials [63, 64, 66, 67, 74, 77–79]. Agee et al. provided a single educational seminar for Family Medicine residents [63]. Belfield et al. provided weekly education (including a literature review of stress ulceration in non–critically ill patients and the appropriate indications for AST) on the initiative for 2 months [64]. Prior to completing rounding activities, Carey et al. educated pharmacy students on evidence-based AST in internal medicine patients and the impact of unnecessary AST [66]. Hammond et al. provided medical residents and pulmonary/critical care fellows, in their first week in the ICU, with an educational intervention lasting 5 minutes on guideline-recommended AST during the six-month intervention period [67]. Tasaka et al. provided presentations to various clinician groups (surgery teams, medicine residents, anesthesia residents, dieticians, ICU nurses, and pharmacists); training sessions were repeated monthly to improve awareness of appropriate SUP use [74]. In Van der Linden et al., pharmacists were trained on the application of the RASP list, prior to completing medication reconciliation activities [79]. Zeigler et al. provided education to clinical staff, which included physicians (both attending and resident staff), pharmacists, and nurses, about the medication reconciliation process and the role of each healthcare professional; education was done via educational classes, a Web-based training module, presentations at hospital committee meetings, and one-on-one communication (SUP was not included as part of this education) [77]. Ziegler et al. conducted in-person education in the initial month after guideline introduction for the non-house staff teams with a 30-minute session focused on reviewing the evidence supporting their guideline and recommended strategies for AST use; due to frequent change in house staff providers on the inpatient hematology-oncology services, twice monthly in-person orientations were held for the duration of the intervention period [78].

Additional education on appropriate AST/SUP use occurred in the form of hospital newsletters (Tasaka et al.) [74]; development of facilitator guides to use during teaching rounds (Tasaka et al.) [74]; e-mails to clinical staff (2 studies: Tasaka et al. and Khudair et al.) [70, 74]; pocket cards (4 studies: Agee et al., Hammond et al., Hatch et al., Tasaka et al.) [63, 67, 68, 74]; handout/memorandum (3 studies: Agee et al., Khudair et al., Hatch et al.) [63, 68, 70]; and posters/flyers (2 studies: Tasaka et al. and Khudair et al.) [70, 74].

#### Medication reconciliation

According to the Institute for Healthcare Improvement, medication reconciliation is the process of creating the most accurate list possible of all medications a patient is taking (drug name, dosage, frequency, and route) and comparing that list against the physician’s admission, transfer, and/or discharge orders, with the goal of providing correct medications to the patient at all transition points within the hospital [93]. Six studies described pharmacy involvement in completing medication reconciliation activities [68, 73, 76, 77, 79] Hatch et al. reported that pharmacists conducted medication reconciliation at all points of care; additionally, a clinical pharmacist evaluated the discharge medication list and provided medication counseling to each patient prior to their departure, allowing pharmacists the opportunity to identify and discontinue unnecessary medications [68]. Pavlov et al. enlisted emergency room pharmacy technicians to collect medication-related data from various sources to compile thorough medication reconciliation during admission; nurses completed medication reconciliation at discharge [73]. Van der Linden et al. trained clinical pharmacists to perform medication reconciliation by first applying the RASP list followed by an additional comprehensive medication review to determine prolonged PPI use; the RASP list was again applied to discharge medications [79]. Wu et al. reported medication reconciliation of newly admitted patients being completed by a clinical pharmacist with a focus on PPIs; if a PPI was part of the list, but without an evidence-based indication, a recommendation to the medical team was made if patient was agreeable to discontinuation [76]. Zeigler et al. reported that the medication reconciliation process could be completed by either pharmacists or nurses during admission, with a physician reviewing/adjusting medication lists upon admission and at care transfer [77]. While Khudair et al. did not state that medication reconciliation occurred, an assessment of AST appropriateness upon admission and at discharge was completed [70].

#### Recommendations

Ten studies provided recommendations about appropriate AST prescribing to health care providers in-person (e.g., during rounds), electronically, and/or via print [64, 66–70, 74, 76, 78, 79].

Belfield et al. reported that clinical pharmacists reviewed the majority of previously verified AST orders from a daily census list; if an inappropriate order was identified, the pharmacist contacted the hospitalist to clarify the indication and recommend discontinuation of the order [64]. Carey et al. instructed 4^th^ year pharmacy students completing advanced pharmacy practice experiences to participate in patient rounds and address therapeutic issues; all student recommendations to discontinue AST for patients were verified by a pharmacist preceptor who approved the recommendation or advised the student to communicate an alternative recommendation to the physician [66]. Hammond et al. reported that a clinical pharmacist rounded with the medical ICU treatment team daily [67]. Hatch et al. reported that pharmacists were encouraged to approach prescribers to discuss appropriateness of continuing AST and to recommend that they be discontinued if there was not a clear indication [68]. Hughes et al. reported that a clinical pharmacist was present during weekly interdisciplinary rounds to offer recommendations [69]. Khudair et al. reported that clinical pharmacists rounded with the clinical teams on a daily basis (5 days a week) and ensured that the prescribers used AST materials (guidelines, algorithm) for all relevant patients [70]. Tasaka et al. reported that ICU pharmacists completed a daily review of medication administration record and pharmacists directly communicated any SUP issues with the prescribing physician with follow-up within 48 hours [74]. Van der Linden et al. reported that pharmacist-led recommendations were actively reported to the treating physician on a daily basis but that it was left to the discretion of the treating physician as to whether to follow the pharmaceutical recommendations [79]. Wu et al. recommended to the medical team to deprescribe PPI in patients found to be on a PPI without an evidence-based indication (if the patient is agreeable to trial discontinuation) [76]. Lastly, Ziegler et al. reported that a hematology/oncology-trained pharmacist rounded 5 days/week and was responsible for enforcing/re-educating health care providers about AST guidelines [78].

#### Prescribing

In two studies, pharmacists adjusted AST within institutionally-approved protocols. Buckley et al. stated that clinical pharmacists had prescriptive authority to initiate, modify, or discontinue SUP within the context of a defined institutional protocol using computerized prescriber order entry [65]. Pharmacists were allowed to discontinue SUP lacking an appropriate indication or, once major risk factors were resolved in ICU patients. In the non-ICU setting, pharmacists were allowed to discontinue AST in patients without an indication and who were not on AST prior to hospital admission. All AST orders from clinical pharmacists were forwarded to physicians through the electronic medical record requesting their electronic signature authorizing the order [65]. Martz et al. utilized an automatic pharmacist-driven discontinuation protocol to reduce inappropriate SUP during ICU discharge [72].

### Quality assessment

Methodological assessment was performed using Newcastle-Ottawa scale [58] and is reported on Table 3. Most publications were classified as good while four (Carey et al., Wohlt et al., Hatch et al. and Martz et al.) were classified as satisfactory [66, 68, 72, 75].

**Table 3:** Detailed Newcastle-Ottawa Scale [58] of each included cohort study.

	Selection				Comparability		Outcome			Total Quality Score
Study	Representativeness of exposed cohort	Selection of non-exposed cohort	Ascertainment of exposure	Demonstration that outcome of interest was not present at start of study	Adjust for most important risk factors	Adjust for other risk factors	Assessment of outcome	Follow-up length	Loss to follow-up rate	
**ICU setting**										
Buckley [65]	1	1	1	1	0	0	1	1	1	**7**
Hammond [67]	1	1	1	1	0	0	1	1	1	**7**
Martz Abstract [72]	1	1	0	1	0	0	0	1	1	**5**
Pavlov [73]	1	1	1	0	1	1	1	1	1	**8**
Tasaka [74]	1	1	1	1	0	0	1	1	1	**7**
Wohlt 2007 [75]	1	1	1	0	0	0	1	1	1	**6**
Hatch 2010 [68]	1	1	1	0	0	0	1	1	1	**6**
Zeigler [77]	1	1	1	1	0	0	1	1	1	**7**
**Non-ICU setting**										
Agee [63]	1	1	1	1	0	0	1	1	1	**7**
Belfield [64]	1	1	1	1	0	0	1	1	1	**7**
Buckley [65]	1	1	1	1	0	0	1	1	1	**7**
Carey [66]	1	1	1	0	0	0	1	1	1	**6**
Hughes [69]	1	1	1	1	0	0	1	1	1	**7**
Khudair 2011 [70]	1	1	1	1	0	0	1	1	1	**7**
Khudair 2009 [71]	1	1	1	1	0	0	1	1	1	**7**
Van der Linden [79]	1	1	1	1	0	0	1	1	1	**7**
Wu Abstract [76]	1	1	1	1	0	0	1	1	1	**7**
Ziegler [78]	1	1	1	1	0	0	1	1	1	**7**

#### Selection

All studies in the ICU setting used a sample representative of the general population and selected the non-exposed cohort from the same community as the exposed cohort [65, 67, 68, 72–75, 77]. In the non-ICU setting, most studies included patients admitted to the medical floor or general ward and used a sample representative of the general population (6 studies: Agee et al., Belfield et al., Buckley et al., Carey et al., Khudair et al., Wu et al.) [63–66, 70, 71, 76]. However, three studies included varied populations of patients. In Hughes et al., patients were admitted to the Geriatric Evaluation and Management unit (medically-stable patients admitted for rehabilitation for ≥7 days) [69]; Van der Linden et al. admitted very old patients (average age 85 years) to acute geriatric wards [79]; and Ziegler et al. admitted patients to hematology-oncology units [78]. The non-exposed cohort was selected from the same community as the exposed cohort. These varied populations are similarly at increased risk for AST overuse and subsequent exposure to adverse events and costs (as those admitted to the ICU or medical floors) [69, 78, 79].

Seven studies provided patients’ age and/or gender [64, 65, 67, 68, 70, 71, 73, 75, 79] and several described differences between the intervention group versus comparator group. In the ICU setting, Buckley et al. reported a significantly (P<0.05) higher proportion of males in the post-intervention group (51.7% versus 65.9%) and a higher proportion of patients with acute myocardial infarction in the pre-intervention group (9.8% versus 3%) [65]. In Hammond et al., patients were older and more likely to have ≥2 risk factors for SUP indication in the intervention group (51.07 versus 56.24 years and 28% versus 42%, respectively) [67]. In Pavlov et al., more patients in the pre-intervention group were older (65.5 versus 62.4 years), male (60% versus 51%), and had multiple comorbidities (3.5 versus 2.7), respectively [73]. In Hatch et al., a higher proportion of the post-intervention group was admitted to medical ICU (35% versus 50%) while a higher proportion in the pre-intervention group (Wohlt et al.) received a cardiovascular admission diagnosis (26% versus 18%) [68, 75].

In the non-ICU setting, Buckley et al. reported a longer average length of stay (6.8 versus 10.5 days) in the post-intervention group and a higher proportion with acute myocardial infarction in the pre-intervention group (8% versus 1.5%) [65]. In Carey et al., a higher proportion of patients in the pre-intervention group had a neurologic diagnosis (13% versus 5.6%) while more patients in the post-intervention group had an infectious disease diagnosis (15.1% versus 23.9%) [66]. In Khudair et al., a majority of patients in both pre- and post-intervention groups were males (73%) [70, 71]. No differences in patient demographics were reported by Belfield et al. [64] and Van der Linden et al. [79].

All studies, except for Martz et al. [72] documented using secure medical records to ascertain exposure and assessed outcomes using record linkage. While the assumption is that secure medical records were used for data collection, Martz et al. [72] did not clearly report this in their abstract.

The primary outcome of this meta-analysis was to determine the proportion of patients on inappropriate AST at discharge. Patients who were newly-initiated on AST while in the hospital did not have the outcome of interest at the start of the study [64, 65, 67, 72, 77]. However, patients could be on AST (appropriately or inappropriately) prior to arrival to the institution. If all included patients (regardless of whether they were started on AST during hospitalization or arrived already on AST) were assessed for appropriateness, then those studies were given a point for completing the assessment [63, 70, 74, 76, 78, 79]. Studies where AST prior to arrival were considered “appropriate” (without further assessment) [66, 68, 75] or if data on appropriateness of those on AST prior to arrival were not clearly stated [73], received zero points for this variable.

Wohlt-Hatch et al. reported that all patients who had been prescribed AST prior to hospital admission were considered to be discharged appropriately on these medications [68, 75]. Carey et al. did not require pharmacy students to verify the indication for home AST use, therefore patients admitted to the hospital already on inappropriate AST would not have been targeted for a pharmacy student intervention [66]. Pavlov et al. reviewed in detail the paper medical records (physician progress notes, respiratory care data) of discordant cases (patients not treated with AST prior to admission but who were discharged on them and those who had received them prior to admission but were discharged without them (for no clear reason)) [73]. However, it was unclear if patients were on inappropriate AST prior to arrival were assessed for appropriateness during the study.

#### Comparability

Three studies conducted statistics to identify any factors that may be associated with the likelihood of being discharged on inappropriate AST. Pavlov et al. performed unconditional multiple logistic regression analyses to examine predictors of inappropriate discharge on AST and found the significant predictors included being in the pre-implementation group (adjusted OR 2.5), presence of coronary artery disease (adjusted OR 3.4), prolonged hospital stays ≥15 days (adjusted OR 2) and being mechanically ventilated (adjusted OR 1.9) [73].

In Zeigler et al., the use of appropriate versus prolonged SUP across various clinical conditions were evaluated and patients with a head injury were more likely to have SUP continued appropriately from ICU to a non-ICU setting [77]. However, no points were awarded as there was no adjustment for risk factors that might impact inappropriate AST use at hospital discharge. In the non-ICU setting, Carey et al. used linear regression, adjusting for length of stay and clustering within physician, to determine the pharmacy students’ impact on inappropriate AST prescribing [66]. A significant reduction in the number of days patients were on inappropriate AST was found [66], however, adjustment for risk factors was not performed to determine any predictors for being on inappropriate AST upon discharge. Therefore, no points were awarded for comparability.

#### Outcome

For all studies, data were extracted from medical records and medication profiles. Martz et al. [72] received no points for Assessment of Outcome as it was not made clear in the abstract if secure medical records were used for outcome assessment.

Data collection periods ranged from two weeks to eleven months and were as follows: Tasaka et al. (two weeks) [74]; Belfield et al., Buckley et al., Wu et al., and Zeigler et al. (one month) [64, 65, 76, 77]; Khudair et al. (two months) [70, 71]; Agee et al., Martz et al., Pavlov et al., Wohlt-Hatch et al., and Ziegler et al. (three months) [63, 68, 72, 73, 75, 78]; Carey et al. (four months) [66]; Hammond et al. (six months) [67]; Hughes et al. (10 months) [69] and Van der Linden et al. (11 months) [79]. Data collection periods were considered appropriate in all studies and as all studies completed data extraction retrospectively, all records were accounted for.

### Meta-analysis

#### ICU setting

**Fig 2:**
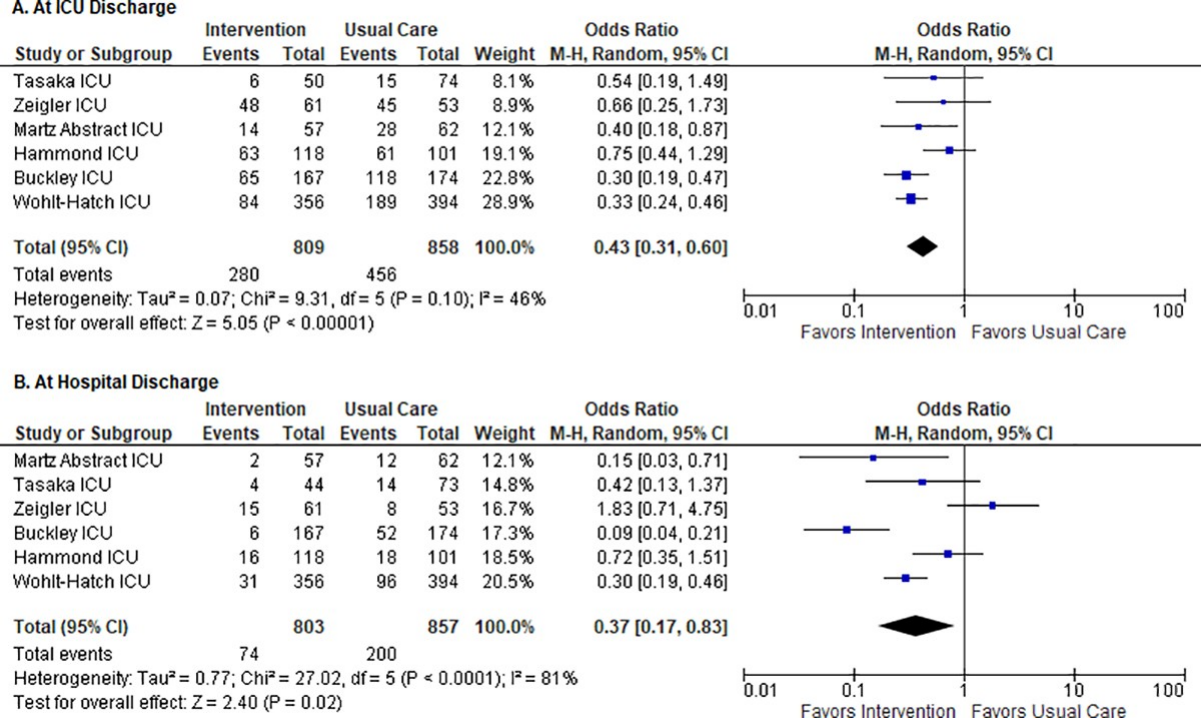
Proportion of patients on inappropriate AST at ICU (A) and at hospital discharge (B).

Most pharmacy-supported interventions occurred within the ICU setting in seven ICU studies (Buckley et al. [65], Hammond et al. [67], Martz et al. [72], Tasaka et al. [74], Zeigler et al. [77] and Hatch et al. [68]); in Pavlov et al., emergency department pharmacy technicians conducted comprehensive medication reconciliation on admission [73]. To assess the effect of these interventions during ICU stay, meta-analysis was conducted for data provided by six studies on the proportion of patients discharged from the ICU on inappropriate SUP therapy (these data not reported by Pavlov et al.). Meta-analysis showed a significant reduction in the odds of being discharged on inappropriate SUP at this timepoint in the pharmacy intervention study arm (OR 0.43 [95% CI 0.31 to 0.60]), with low heterogeneity (*I*^*2*^ *=* 46%) (Fig 2A). A review of the forest plot showed the lowest OR was with Buckley et al. (OR 0.30 [95% CI 0.19 to 0.47]), where pharmacists with prescriptive authority adjusted SUP based on a protocol [65].

At hospital discharge, meta-analysis showed a significant reduction in the odds of being discharged on inappropriate AST in the pharmacist intervention arm (OR 0.37 [95% CI 0.17 to 0.83]), with significant heterogeneity (*I*^*2*^ = 81%) (Fig 2B). Buckley et al. was again associated with a significant reduction in the proportion of patients discharged from the hospital on inappropriate AST (OR 0.09 [95% CI 0.04 to 0.21]) [65].

When reviewing the odds of being discharged from the ICU (OR 0.43) and then at hospital discharge (OR 0.37), it appears as if the interventions made in the ICU had a positive impact throughout the patient’s hospital stay. Of note, in three of the seven studies, pharmacy-supported interventions occurred during the patient’s ICU stay but may have also occurred during their stay in the non-ICU setting and then at discharge. In Buckley et al., pharmacists had prescriptive authority in both the ICU and non-ICU settings [65]; in Wohlt-Hatch et al., pharmacists used a pocket card with SUP indications to aid with medication reconciliation during care transfer [68, 75], and in Zeigler et al., pharmacists were taught the medication reconciliation process, but prescribers reviewed medication profiles during care transfer [77].

#### Studies in the non-ICU setting

**Fig 3:**
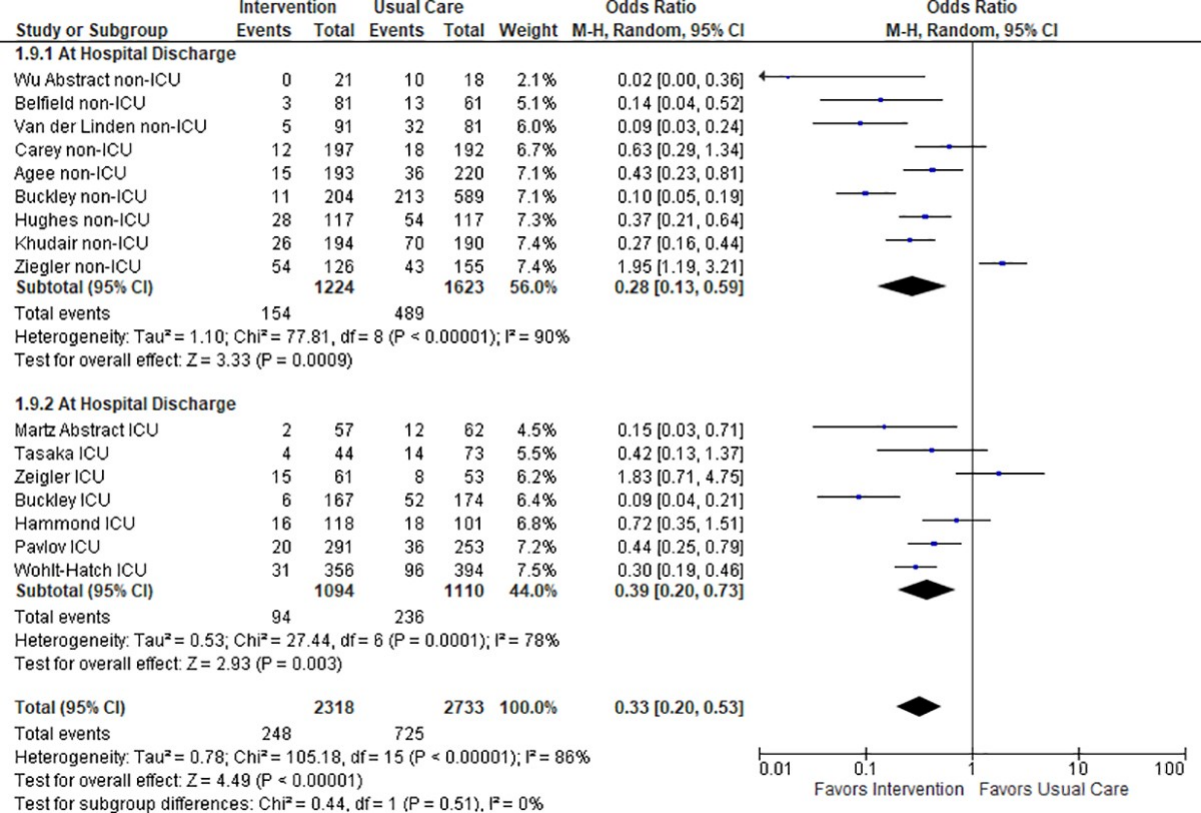
Proportion of patients on inappropriate AST at hospital discharge.

Patients admitted to a non-ICU setting can originate from various settings including the ICU or from previous living arrangements. Meta-analysis showed a significant decrease in the proportion of patients discharged on inappropriate AST from the hospital in the pharmacist intervention arm (OR 0.28 [95% CI 0.13 to 0.59]), with significant heterogeneity (*I*^*2*^ = 90%) (Fig 3). When reviewing the forest plot, all studies, except for Carey et al. and Ziegler et al., showed a significant reduction in ORs. In these two studies, patient-specific interventions were made by pharmacy students and/or pharmacists during rounding [66, 78].

#### Combined effect of all studies

Most patients discharged from the ICU setting were transitioned to the general medical floors and eventually discharged from the hospital [65, 67, 68, 72, 74,75, 77]. Therefore, to determine the proportion of patients discharged from the hospital on inappropriate AST, regardless of admission setting or where the interventions occurred, data from all studies were combined. Meta-analysis showed a significant reduction in the odds of being discharged on inappropriate AST from the hospital in the pharmacist intervention arm versus comparator (OR 0.33 [95% CI 0.20 to 0.53]), with significant heterogeneity (*I*^*2*^ = 86%) (Fig 3).

Additional subgroup analyses were conducted to possibly explain heterogeneity (S5 File). Factors analyzed included study location (USA versus non-USA); study design (retrospective versus prospective); study settings (medical floor/general ward versus other settings (Geriatric Evaluation and Management unit at the Veterans Affairs Medical Center, acute geriatric wards, hematology-oncology units)); studies that included patients on AST prior to arrival versus those which included only patients newly-started on AST; data collection observation periods (≤15 weeks versus >15 weeks); pharmacist intervention (prescribing versus other interventions); and NOS total score (<7 versus ≥7). Most subgroup analyses showed no difference between the subgroups.

Since nine studies used a multi-faceted approach [63, 64, 66–68, 70, 74, 78, 79], it was inappropriate to analyze each intervention component individually to determine how each contributed to our outcome of interest. Therefore, we conducted subgroup analysis of interventions where the pharmacist adjusted AST within institutionally-approved protocols versus all other interventions (S5 File, subgroup 6). When subgroup analysis was conducted based on type of intervention (pharmacist prescribing versus other), the difference between the two groups were significant, in favor of pharmacist prescribing (OR 0.1 [95% CI 0.06 to 0.16], (*I*^*2*^ = 0%).

#### Funnel plot optimization method

**Fig 4:**
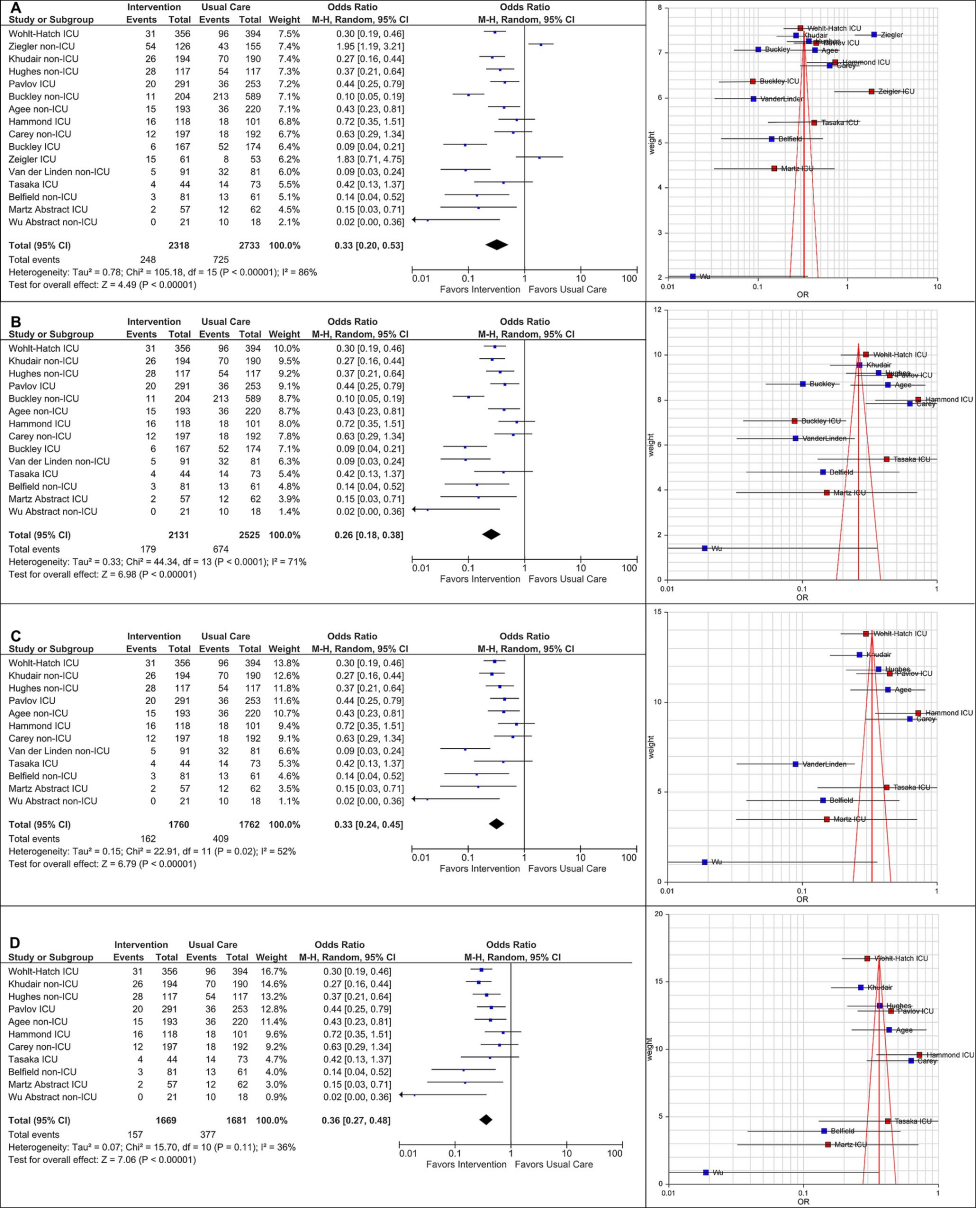
Distillation of studies using funnel plot optimization method.

For our primary outcome, patients in the pharmacist-supported intervention group were less likely to be discharged on inappropriate AST (OR 0.33 [95% CI 0.2 to 0.53], p <0.00001). However, heterogeneity was significant at *I*^*2*^ = 86% (Fig 4A). Using funnel plot methodology, the Zeigler et al. [77] and Ziegler et al. [78] studies were then removed (Fig 4B), resulting in OR 0.26 [95% CI 0.18 to 0.38], p <0.00001, with improved but still significant heterogeneity (*I*^*2*^ = 71%).

These studies were removed as they appear to underestimate the effect size. Results for Ziegler et al. [78] favored usual care (OR 1.95 [95% CI 1.19 to 3.21]) while those of Zeigler et al. [77] showed that pharmacy-supported interventions had no effect on the proportion of patients discharged on inappropriate AST (OR 1.83 [95% CI 0.71 to 4.75]). In Ziegler et al. [78] the hematology/oncology pharmacist was involved in multiple activities (guideline development, dissemination, and rounding). In Zeigler et al. [77] the pharmacy-supported intervention was to educate all health care providers on the medication reconciliation process with the primary physician reviewing medication profiles at care transfer. Both authors noted that additional interventions were needed to address PPI orders with unclear indications or prolonged use and their continuation at discharge [77, 78].

Fig 4C displays the forest plot after removal of ICU and non-ICU data from Buckley et al. [65] as this study appears to overestimate the effect size. The OR was 0.33 [95% CI 0.24 to 0.45], p <0.00001), with lower but still significant heterogeneity (*I*^*2*^ = 52%). Buckley et al. [65] reported that pharmacists had prescriptive authority to adjust AST orders as per institutional guidelines but that the intervention was only evaluated one-month post-implementation.

Fig 4D illustrates removal of the final study (Van der Linden et al. [79]) as this study also overestimated the effect size, resulting in OR 0.36 [95% CI 0.27 to 0.48], p <0.00001), with low heterogeneity (*I*^*2*^ = 36%). Van der Linden et al. [79] reported that pharmacists completed medication reconciliation (with RASP list) upon admission and at discharge, along with a comprehensive review if a potentially inappropriate medication was found, attended rounds, and sent daily recommendations to the treating physicians about medication concerns [79].

#### Publication bias

Publication bias was noted as Fig 4D demonstrated a lack of negative high-powered studies (to our knowledge) to balance the Hatch et al. [68] and Khudair et al. [70] studies. Moderately-powered negative studies to balance the Hammond et al. [67] and Carey et al. [66] studies were also missing.

#### Publication bias by subgroups

Publication bias was noted for the following subgroups: studies conducted in other countries, studies conducted prospectively, studies with a longer observation period, studies where pharmacists were involved with prescribing, and studies where the Newcastle-Ottawa score was < 7. Publications were uniformly biased toward positive studies in these subgroups.

## Discussion

This systematic review and meta-analysis of 16 studies from 17 publications showed that pharmacy-supported interventions in ICU and non-ICU settings were associated with a significantly reduced probability of patients discharged on inappropriate AST (OR 0.33 [95% CI 0.2 to 0.53]), but with significant heterogeneity (*I*^*2*^ = 86%). Eleven studies from 12 publications favored pharmacy-supported interventions [63–65, 68–73, 75, 76, 79], four studies did not show a benefit [66, 67, 74, 77] and one study favored usual care [78]. Several reasons observed by the authors as to why their pharmacy-supported interventions were unsuccessful included 1) possibility of gaps in the educational process (e.g., not all health care providers were in attendance during presentations); 2) absence of pharmacy presence during night and weekend patient rounds and at care transfer; and/or 3) undocumented reasons for continuing AST [66, 67, 74, 77, 78].

As subgroup analyses did not explain heterogeneity, funnel plot optimization method was employed. This method is a quality control measure that allowed us to closely examine each study’s contribution to the overall effect size and provided guidance on which studies (positive [65, 79] and negative [77, 78]) to remove in an objective manner. Sequential removal of Zeigler et al. [77], Ziegler et al. [78], Buckley et al. [65], and Van der Linden et al. [79] allowed for meta-analysis of 11 studies from 13 publications, resulting in a similar OR, but with less heterogeneity (OR 0.36 [95% CI 0.27 to 0.48], *I*^*2*^ = 36%).

The initial two studies removed were by Zeigler et al. [77] and Ziegler et al. [78]. These studies were retrospective and associated with an increased likelihood of being discharged on inappropriate AST from the hospital. Interventions in these studies differed. In Zeigler et al., the medication reconciliation process was taught (by pharmacists) to multiple health care providers (including other pharmacists), but no patient-specific interventions to address inappropriate AST was reported during the ICU stay, non-ICU stay or at discharge [77]. In contrast, pharmacy-supported interventions conducted by Ziegler et al. were multi-faceted and occurred during hospitalization in hematology-oncology patients. The author suggested an opportunity for additional improvement to address the high rates of orders with unclear indications and continuation on discharge [78].

The third publication that was removed was by Buckley et al. [65]. This was a retrospective study where the intervention was prescriptive authority by pharmacists and was associated with a significant reduction in the odds of being discharged on inappropriate AST, regardless of setting (ICU setting: OR 0.09 [95% CI 0.04 to 0.52] and non-ICU setting OR 0.10 [95% CI 0.05 to 0.19]). Although the observation period was only one month, the sample size was moderately large suggesting that their intervention may have resulted in improved time efficiency for both pharmacists and physicians [65]. The final study removed was by Van der Linden et al., which was a prospective, controlled study where pharmacists completed medication reconciliation upon admission and at discharge by applying a list of 76 items to capture potentially-inappropriate medications (i.e., prolonged AST use) in patients admitted to a non-ICU setting (acute geriatric wards); patients in the control group were managed by an experienced geriatrician [79]. While this study employed a nonrandomized design (similar to all other included studies), it was prospective, had a control group and collected data for both groups during the same time period.

Although the two publications showing a positive effect of pharmacist-supported interventions on our outcome of interest were removed, we believe additional studies utilizing these strategies are warranted to verify durability of effect. In both cases, pharmacists were available throughout the patient’s stay (from admission to discharge) to review medication lists for appropriateness, and in Buckley et al., pharmacists had prescriptive authority to modify AST [65, 79].

Possible reasons for significant heterogeneity found in the first meta-analysis of the 16 studies from 17 publications merit further discussion. Both heterogeneity and quality of the studies influence the overall effect of our meta-analysis. In addition to the factors explored in subgroup analyses, other sources of heterogeneity that could affect our outcome of interest may be found in both the diverse inclusion/exclusion criteria and the definition of appropriate and inappropriate AST as outlined in the studies. While recognizing the problem, we believe that these factors are so intrinsically connected to each single institution that their standardization is difficult. Health care providers, including pharmacists, practice within the strengths and limitations unique to each institution and will adapt guidelines to optimally manage the patients in their care.

Most studies used in our meta-analysis were retrospective in nature and therefore prone to different bias. The efficiency and reduced costs that retrospective studies offer is counter-balanced by the inherent possibility of erroneous/incomplete data. While the advantage of using medical records for retrospective data collection include accessing a large amount of clinical information to adequately assess quality improvement in clinical practice [94], limitations include incomplete or missing data within the record, difficulty in interpreting or verifying documented information and variability in the quality of documentation among health care providers [95].

Some of these risks could be alleviated by having pre-post intervention data collected prospectively. While concurrency of control and intervention groups would be ideal, we understand that aspects such as the type of intervention may dictate how control and intervention groups are determined. For example, while giving prescribing authority to a clinical pharmacist who can operate within a specific ward may make it possible to have concurrent control and intervention groups, an intervention based on education, which to be effective needs to be widespread, would probably be best served by a before-after type of study to avoid cross-contamination between study arms.

Furthermore, since randomized clinical trials may not be feasible, a possible alternative could be a pre-post, multi-arm, prospective study design which can include sampling randomization [96]. With this design, data for both groups (pre and post) would be collected prospectively to mitigate misclassification. For example, after sample size calculations and explanation to data extractors on methods to completing the data collection tool, pre-intervention data of patients that meet inclusion criteria would be collected prospectively, followed by implementation of the intervention, and then data collection during intervention or post-intervention period. If the dataset is large, the researcher may perform random sampling of the pre and post group for analysis.

Additional recommendations to increase the consistency of studies describing pharmacist-led interventions include identifying data source(s), creating a data extraction instrument, training data extractors (preferably two extractors), re-evaluating a small dataset to check for agreement, and conducting appropriate statistical analysis, including cost avoidance analysis [94, 97, 98]. The process ultimately utilized should be published (e.g., as a supplement) for other health care providers and for administrators considering evidence-based practices for optimal pharmacy-resource allocation [99]. For example, the methodology should clearly delineate: inclusion and exclusion criteria; outcome of interests; relevant medications patients are receiving prior to arrival and during hospitalization; thorough appropriateness and inappropriateness criteria; when and how these criteria were applied to the patients’ medication lists to determine appropriateness; what tasks pharmacists were already engaged in versus new pharmacy-supported interventions; the timepoint during the patient’s hospital stay that the intervention occurred (upon admission, during stay, and/or upon discharge); detailed description of intervention; the timepoint when assessments of the interventions occurred (in real-time versus retrospectively); when another health care provider was involved with the patients’ medication reviews and at what timepoints; documentation of acceptance/rejection of pharmacist interventions and interventions made by other health care providers; patient demographics (age, gender, race/ethnicity, etc.); factors that may influence the likelihood of the outcome of interest by adjusting for differences in baseline demographics or other clinical factors [54, 100].

Another element that may have affected our outcome of interest is the limitations of the type of pharmacy-supported interventions employed in the included studies. If inappropriate AST was identified, a member of the pharmacy staff (e.g., pharmacists, pharmacy students, pharmacy technicians) communicated concerns with the health care provider during rounds, in-person and/or electronically. Pharmacy staff also completed medication reconciliation that was later reviewed by health care providers [64, 66, 67, 69, 70, 73, 74, 76–79]. It, therefore, remained at the discretion of the health care provider to either ignore or consider pharmacy-proffered recommendations or make adjustments based on their own review of medication reconciliation [100]. However, once a recommendation was submitted, any number of issues could have arisen preventing the health care provider from addressing that recommendation. In a study by Guignard et al. [41], pharmacists identified 161 drug-related problems and provided verbal recommendations during rounds for treatment modification or initiation of monitoring. Of these, 84% were accepted by physicians; however, 31% (42 of 135 accepted verbal suggestions) were ultimately not followed. The authors suggested the possibility that treatment optimization suggestions were not implemented due to a busy workload leading to forgetfulness by the medical resident or lack of validation by their supervisors not present during medical rounds [41].

With prescriptive authority, as described by Buckley et al. [65], the pharmacist made timely adjustments to pharmacotherapy as delineated in a specific institution-approved collaborative practice agreement. Moreover, this clinical pharmacy program resulted in a significant reduction in the rate of inappropriate SUP days in the ICU and non-ICU settings and an estimated annual cost savings of $200,000 USD [65]. To maximize the benefits of pharmacists’ skills in optimizing medications in an efficient and effective manner necessitates pharmacists obtaining the ability to initiate and alter medications as appropriate [101].

Collaborative practice agreements (CPAs) is a method for pharmacist prescriptive authority [101, 102]. CPAs typically provide authority for medication management of acute or chronic diseases in which a diagnosis has been made [101, 102]. In a review of published literature reporting on pharmacist prescribing in the US, professional association material and relevant individual state practice acts, legislation, and regulations, Sachdev et al. reported that currently, pharmacists are legally authorized to participate in pharmacist collaborative drug therapy management (CDTM) in 49 of the 50 states and the District of Columbia (Delaware is the only state without CDTM legislation) [101]. However, CDTM laws and regulations vary widely among the 49 states and the District of Columbia. When developing a CPA, the authors recommend reviewing both state pharmacy and medical laws for: pharmacist qualifications to participate in the agreement; providers allowed to delegate services to a pharmacist; required relationship between the delegating provider, pharmacist, and patient involved; services and activities allowed and not allowed to be delegated; number of pharmacists, providers, and/or patients allowed in an agreement; supervision and authorization requirements; practice site requirements; and specifics such as renewal time for agreements [101].

Three studies reported that pharmacy staff was unable to complete interventions during nights and weekends [66, 67, 70]. The 2018 ASHP national survey of pharmacy practice in hospital settings reported that 60.8% of hospitals routinely had pharmacists assigned to provide drug therapy management services to a majority of patients in the hospital at least 8 hours per day, 5 days per week [103]. Time and staff commitments in contacting health care providers with recommendations and waiting for the recommendation to be acted upon may be lessened if pharmacists had prescriptive authority within institutions; collaborative practice agreements may remove the burden of contacting prescribers especially during nights and weekends.

A systematic review comparing pharmacist prescribing to medical prescribing in the hospital setting demonstrated that pharmacist prescribing is at least as safe as doctor prescribing [104]. Pharmacists were better at adhering to dosing guidelines when prescribing by protocol and made significantly less prescribing errors when charting patients’ usual medications on admission to hospital [104]. Seven studies in our meta-analysis reported pharmacy participation in preparing and/or distributing institution-specific guidelines and algorithms to determine which patients were appropriate candidates for AST [63, 64, 67, 68, 70, 74, 78]. While these resources were available to health care providers electronically and/or as printed pocket cards, they may have been underutilized [105]. With a number of health information technology platforms currently available, access to these medication management policies and guidelines during the medication ordering, verification, and administration screens may be facilitated by hyperlinks added within the electronic medical record [106].

Twelve publications (resulting in ten studies) [63, 66, 68–71, 73–76, 78, 79] included patients who were on AST prior to arrival. Of these, two considered patients to be on AST appropriately [66, 68] and one did not clearly state how prior AST use was addressed [73]. Patients on AST prior to arrival could have been on a PPI with an appropriate indication or as part of self-care [107]. Despite published guidelines and the well-known indications for appropriate PPI use, these agents are overused worldwide in both the hospital and community settings [29, 34, 108–111]. Furthermore, PPIs are also available for over-the-counter (OTC) purchase as part of self-care; however, with recent concerns about impurities of ranitidine and nizatidine (both H_2_RAs), patients may preferentially purchase OTC PPIs rather than H_2_RAs [112, 113]. Therefore, a difficult and frequently-encountered clinical situation is not being able to determine why a patient is taking AST as the patient may not know or remember the indication, and/or the indication was never documented [38]. Prescribers encountering these patients in various settings may fear negative consequences or may not have adequate time and/or receive proper reimbursement to address AST appropriateness [32]. Therefore, prescribers may avoid deprescribing AST even though the patient may be receiving a potentially inappropriate medication [114, 115].

Availability of pharmacists to conduct medication reconciliation is viewed as an enabler to addressing potentially inappropriate chronic medications [114]. Although time-consuming, obtaining a thorough and accurate medication list is important in preventing drug-related problems and other pharmacy staff can assist with medication reconciliation activities. Pavlov et al. [73] enlisted pharmacy technicians in the emergency room to compile a thorough list, from multiple sources, during admission. Although the outcome of this study favored intervention, Pavlov et al. [73] did not clearly describe the appropriateness of patients’ AST use prior to arrival.

While this meta-analysis focused on pharmacy-supported interventions to reduce inappropriate AST use at discharge, patient collaboration in the deprescribing process is important as symptom control is highly-valued [116]. Wu et al. [76] was the only study to report communicating with the patient first and then discussing recommendations with the health care team. It is difficult to know what steps to take if both the patient and the health care provider do not know why the patient is on a PPI. Pasina et al. found that 48% of patients in the community were treated with a PPI for an unlicensed indication [111]. Furthermore, while patients may be in favor of discontinuation, they are hesitant to discuss deprescribing with their prescribers [111]. Therefore, to facilitate appropriate PPI use, health care providers should initiate a conversation about deprescribing with the patient [12].

Another strategy to reduce overuse is to document directly onto the prescription the indication and duration of use for the prescribed PPI. An analysis of 135,340 outpatient prescriptions for ulcer medications showed only 2.67% had an indication [117]. This information may then be obtained if hospital pharmacy staff, such as a pharmacy technician, contacts patients’ pharmacies during medication reconciliation activities. To further improve medication reconciliations, pharmacists who recommend PPIs in the outpatient setting must be able to identify patients who would benefit from PPI use, explain duration of therapy and the need for primary prescriber visit [118]. To maintain a thorough medication list and avoid future drug-related problems (e.g., therapeutic duplication, drug-drug interactions), the pharmacist should also document this information in the patient’s outpatient/retail pharmacy electronic medical record [119].

This meta-analysis has several strengths and limitations. Our search strategy was comprehensive, included abstracts and grey literature and therefore, sufficiently sensitive for the identification of studies meeting our inclusion criteria. Second, all articles were screened and extracted independently by at least two reviewers. Third, we included a comparator group to assess usual care to determine if pharmacy-supported interventions are necessary. Fourth, while the unique culture and systems in place at each institution along with multi-faceted interventions presented a challenge, they also provided support for generalization of the impact of pharmacist-supported interventions to positively affect the proportion of patients discharged on inappropriate AST. Lastly, based on a thorough review of the included publications, we were able to offer several recommendations to possibly limit bias and improve the design of future studies.

An important limitation was the significant heterogeneity found in the initial meta-analysis of all included studies. However, funnel plot optimization method was utilized to objectively remove studies that over-estimated and under-estimated the effect size, resulting in less heterogeneity. Removal of the outliers still showed that an assessment of the medications, with subsequent interventions to address inappropriateness, appears to result in less patients being discharged on inappropriate AST. While the overall quality of the included studies was satisfactory (Newcastle-Ottawa Scale), a second limitation was the nonrandomized and retrospective study design. Only two studies were conducted prospectively [66, 92] while one study had a retro-prospective design [70]. All remaining studies were retrospectively conducted. Consequently, there may have been other reasons for results that were unable to be observed or controlled for in the included studies. A third limitation was the study setting. While most non-ICU settings were described as the medical floor or general ward, three non-ICU settings provided specialized care (geriatric evaluation and management unit at a Veteran’s Affairs medical center [69], an acute geriatric ward [79] and three hematology/oncology units [78]). Additionally, an understanding and acceptance of the pharmacists’ expanded role beyond dispensing at a specific study setting is subject to the overall culture of the institution [120, 121]. Lastly, not all studies provided demographic data of the included patients (e.g., age, gender) thereby limiting further comparisons.

## Conclusion

To our knowledge, this is the first comprehensive systematic review and meta-analysis to show that pharmacy-supported interventions in ICU and non-ICU settings were associated with a significantly reduced probability of patients discharged on inappropriate AST. However, heterogeneity was significant and not explained by subgroup analyses. Funnel plot optimization method allowed for the objective removal of both positive and negative studies, maintaining a similar reduction in the probability of patients discharged on inappropriate AST, but with less heterogeneity.

To improve study quality, future researchers should include utilizing a pre-post, multi-arm, prospective study design with sampling randomization, identifying data sources, creating a data extraction instrument, training data extractors (preferably two extractors), re-evaluating a small dataset to check for agreement, conducting appropriate statistical analysis and providing a comprehensive methodology in subsequent publications. Lastly, studies are needed to determine if more tailored interventions such as pharmacists with prescriptive authority to adjust pharmacotherapy in real time, in combination with auxiliary staff to assist with medication reconciliations and use of advanced clinical decision support throughout the patient’s hospital stay (including at transitions-of-care), would persistently result in a reduced proportion of patients discharged on inappropriate AST.

## Supporting information

S1 File PRISMA 2009 checklist. (PDF)

S2 File Detailed search strategies. (PDF)

S3 File Modified funnel plot analysis. (PDF)

S4 File Abbreviated description of pharmacist-supported interventions. (PDF)

S5 File Proportion of patients discharged on inappropriate AST from the hospital: Subgroup analyses. (PDF)
