# Supplementary material for: Impact of pharmacy-supported interventions on proportion of patients receiving non-indicated acid suppressive therapy upon discharge: A systematic review and meta-analysis
Source: PLoS One. 2020 Dec 3;15(12):e0243134. doi: 10.1371/journal.pone.0243134 (PMC7714117; doi:10.1371/journal.pone.0243134)
Supplement: S2 File — (PDF) [file pone.0243134.s002.pdf]

Supplement 2. Detailed search strategies

	Ovid MEDLINE® and In-Process & Other Non-Indexed Citations and Daily
1	(inappropriate* OR nonappropriate* OR non appropriate* OR nonindicat* OR non indicat* OR polypharm* OR poly pharm* OR polymedicat* OR poly medicat* OR overprescri* OR over prescri* OR overmedicat* OR over medicat* OR overus* OR mistreat* OR overtreat* OR overutili* OR over utili* OR improper* OR incorrect* OR unwarranted OR unjustified OR erroneous* OR misus* OR un necessar* OR unnecessar* OR repeat* prescri* OR repeat* medicine* OR repeat* medication* OR irrational* OR expensive OR expense* OR expenditure* OR drug related problem* OR medication related problem* OR indiscriminate* OR discrepanc* OR nonguideline* OR non guideline* OR questionable OR omission* OR unlicense* OR unapprov* OR un approv* OR dispens* error* OR wrong* OR near miss OR utilization* pattern* OR utilisation* pattern* OR practice pattern* OR multiple medication* OR non beneficial OR nonbeneficial OR “sub optimal” OR unsafe* OR “over use” OR (with out adj4 indication*) OR (without adj4 indication*)).tw,kf.
2	((improve* OR improving OR optimize* OR optimizing OR optimise* OR optimising) adj3 (safety OR usage OR “use” OR prescri*)).tw,kf.
3	((cost OR costs OR costly OR costing) adj3 (high* OR increase* OR grow* OR financial OR economic OR drug OR drugs OR prescri* OR medicine* OR medication* OR dose* OR dosing OR dosage* OR therap* OR treat* OR “use” OR usage)).tw,kf.
4	(prescri* adj3 (pattern* OR habit* OR practice* OR regimen* OR trend* OR behavior* OR behaviour* OR cascade* OR record* OR indication*)).tw,kf.
5	(reconcil* adj3 error*).tw,kf.
6	(safe* adj3 prescri*).tw,kf.
7	((usage OR “use” OR dosage* OR dose* OR dosing OR medication* OR medicine* OR prescri* OR drug OR drugs OR therap* OR treat* OR indication* OR cost OR costs OR costly OR costing OR dispens*) adj3 (appropriate* OR utilization* OR utilisation* OR justif* OR error* OR

	inadequate* OR excess* OR suboptimal* OR inadvertent* OR rational* OR harmful OR long term OR longterm OR optimal OR reasonable OR reasonably)).tw,kf.
8	(risk* adj3 (usage OR "use" OR medication* OR medicine* OR prescri*)).tw,kf.
9	((nonadhere* OR non adhere* OR noncompliance OR noncompliant OR non compliance OR non compliant) adj3 (guideline* OR indication* OR prescri* OR protocol* OR policy OR policies OR regulation*)).tw,kf.
10	((valid* OR accura* OR approve*) adj3 (prescri* OR indication*)).tw,kf.
11	Inappropriate Prescribing/ OR Polypharmacy/ OR Drug Misuse/ OR Prescription Drug Misuse/ OR Prescription Drug Overuse/ OR Practice Patterns, Nurses'/ OR Practice Patterns, Physicians'/ OR Medication Errors/ OR Medical Errors/ OR Long-Term Care/ OR Drug Utilization/
12	OR/1-11
13	(proton pump inhibitor* OR abeprazan OR benatoprazole OR tenatoprazole OR TU 199 OR CAS 113712-98-4 OR STU-Na OR esomeprazole OR esotrex OR alenia OR escz OR esofag OR nexiam OR ilaprazole OR lansoprazole OR lanzoprazole OR agopton OR bamalite OR inhibitol OR levant OR lupizole OR lanzor OR monolitum OR ogast OR ogastro OR opiren OR prevacid OR prezal OR pro ulco OR promeco OR takepron OR ulpax OR zoton OR dexlansoprazole OR kapidex OR dexilant OR levolansoprazole OR leminoprazole OR linaprazan OR nepaprazole OR omeprazole OR losec OR nexium OR prilosec OR rapinex OR zegerid OR ocid OR lomac OR omepral OR omez OR pantoprazole OR protium OR protonix OR pantotab OR pantopan OR pantozol OR pantor OR pantoloc OR astropan OR controloc OR pantecta OR inipomp OR somac OR pantodac OR zurcal OR zentro OR picoprazole OR potassium competitive acid blocker OR pumaprazole OR rabeprazole OR aciphex OR dexrabeprazole OR pariet OR zechin OR rabecid OR nzole-d OR rabeloc OR revaprazan OR saviprazole OR soraprazan OR tegoprazan OR timoprazole OR vonoprazan).mp.

14	(proton adj2 pump adj2 inhibitor*).tw,kf.
15	((antiulcer OR gastrointestinal OR gastro intestinal OR antireflux OR gastroesophageal OR gastro esophageal OR gastroprotect* OR gastro protect*) adj2 (agent* OR drug OR drugs OR medication* OR medicine*)).tw,kf.
16	(anti adj2 (ulcer OR reflux) adj2 (agent* OR drug OR drugs OR medication* OR medicine*)).tw,kf.
17	((peptic OR stress) adj2 ulcer adj2 (prophylaxis OR prophylactic)).tw,kf.
18	((gastrointestinal OR gastro intestinal) adj2 (prophylaxis OR prophylactic)).tw,kf.
19	((gastric OR acid) adj2 (suppressive OR suppression OR suppressant* OR suppressing OR suppressor*)).tw,kf.
20	antacid*.tw,kf.
21	exp Proton Pump Inhibitors/ OR exp lansoprazole/ or dexlansoprazole/ OR exp omeprazole/ OR esomeprazole/ OR rabeprazole/ OR Anti-Ulcer Agents/ OR Gastrointestinal Agents/ OR Antacids/
22	OR/13-21
23	12 AND 22
24	((proton pump inhibitor* OR ppi OR ppis OR ppi s OR abeprazan OR benatoprazole OR tenatoprazole OR tu 199 OR cas 113712-98-4 OR stu-na OR esomeprazole OR esotrex OR alenia OR escz OR esofag OR nexiam OR ilaprazole OR lansoprazole OR lanzoprazole OR agopton OR bamalite OR inhibitol OR levant OR lupizole OR lanzor OR monolitum OR ogast OR ogastro OR opiren OR prevacid OR prezal OR pro ulco OR promeco OR takepron OR ulpax OR zoton OR dexlansoprazole OR kapidex OR dexilant

	OR levolansoprazole OR leminoprazole OR linaprazan OR nepaprazole OR omeprazole OR losec OR nexium OR prilosec OR rapinex OR zegerid OR ocid OR lomac OR omepral OR omez OR pantoprazole OR protium OR protonix OR pantotab OR pantopan OR pantozol OR pantor OR pantoloc OR astropan OR controloc OR pantecta OR inipomp OR somac OR pantodac OR zurcal OR zentro OR picoprazole OR potassium competitive acid blocker OR pumaprazole OR rabeprazole OR aciphex OR dexrabeprazole OR pariet OR zechin OR rabecid OR nzole-d OR rabeloc OR revaprazan OR saviprazole OR soraprazan OR tegoprazan OR timoprazole OR vonoprazan OR antiulcer agent* OR antiulcer drug* OR antiulcer medication* OR antiulcer* medicine* OR antiulcer treatment* OR antiulcer* therap* OR anti ulcer* agent* OR anti ulcer* drug* OR anti ulcer* medication* OR anti ulcer* medicine* OR anti ulcer* treatment* OR anti ulcer* therap* OR gastrointestinal agent* OR gastrointestinal drug* OR gastrointestinal medication* OR gastrointestinal medicine* OR gastro intestinal agent* OR gastro intestinal drug* OR gastro intestinal medication* OR gastro intestinal medicine* OR antireflux agent* OR antireflux drug* OR antireflux medication* OR antireflux medicine* OR antireflux treatment* OR antireflux therap* OR anti reflux agent* OR anti reflux drug* OR anti reflux medication* OR anti reflux medicine* OR anti reflux treatment* OR anti reflux therap* OR gastroesophageal agent* OR gastroesophageal drug* OR gastroesophageal medication* OR gastroesophageal medicine* OR gastroesophageal treatment* OR gastroesophageal therap* OR gastro esophageal agent* OR gastro esophageal drug* OR gastro esophageal medication* OR gastro esophageal medicine* OR gastro esophageal treatment* OR gastro esophageal therap* OR gastroprotect* agent* OR gastroprotect* drug* OR gastroprotect* medication* OR gastroprotect* medicine* OR gastroprotect* treatment* OR gastroprotect* therap* OR gastro protect* agent* OR gastro protect* drug* OR gastro protect* medication* OR gastro protect* medicine* OR gastro protect* treatment* OR gastro protect* therap* OR gastrointestinal prophylaxis OR gastro intestinal prophylaxis OR peptic ulcer prophylaxis OR stress ulcer prophylaxis OR stress ulcer prophylactic OR acid suppressive OR acid suppression OR acid suppressant* OR acid suppressing OR acid suppressor* OR antacid*) adj3 (appropriate* OR no indication* OR utilization* OR utilisation* OR justif* OR error* OR inadequate* OR excess* OR suboptimal* OR inadvertent*

	OR rational* OR harmful OR long term OR longterm OR risk* OR adverse OR optimal OR reasonable OR reasonably OR cost OR costs OR costly OR costing)).tw,kf.
25	23 OR 24
26	(deprescri* OR de prescri* OR unprescri* OR un prescri* OR discontinu* OR reduction* OR step* down OR stepwise OR step wise OR step off OR compliance OR adherence OR audit OR audits OR taper*OR benchmark* OR bench mark* OR TQM OR academic detailing).tw,kf.
27	(Quality adj1 (control OR improvement* OR assurance OR assess* OR review* OR prescri* OR guideline* OR protocol* OR standard* OR policy OR policies OR regulation* OR management OR intervention OR strategy)).tw,kf.
28	Total quality management/ OR quality control/ OR quality improvement/ OR quality assurance, health care/
29	Benchmarking/ OR management audit/ OR Medical audit/ OR Clinical audit/ OR deprescriptions/ OR patient compliance/ OR medication adherence/ OR guideline adherence/ OR practice guideline/ OR withholding treatment/ OR “treatment adherence and compliance”/
30	((Protocol* OR Guideline* OR policy OR policies OR regulation*) adj3 (compliance OR adhere* OR adhering OR implement* OR evaluat* OR comply OR complying OR compliant OR concord* OR distribut* OR disseminat* OR best practice* OR effective* OR evidence base* OR EBM OR introduce* OR issu* OR impact* OR assess* OR review*)).tw,kf.
31	Pharmacists/ OR Pharmacy Technicians/ OR Students, Pharmacy/ OR Education, Pharmacy, Continuing/ OR Pharmacy Service, Hospital/ OR Community Pharmacy Services/ OR Pharmacy/ OR “Pharmacy and Therapeutics Committee”/ OR Pharmaceutical Services/
32	(Pharmacist* OR pharmd OR Pharmacy OR pharmacies OR pharmacotherapeutic*).tw,kf.
33	(pharmaceutical adj2 (care OR service* OR consult* OR advice OR advise* OR advising OR intervention)).tw,kf.

34	(Screening test* OR screening tool* OR systematic tool* OR Stopp OR start criteria OR beers OR beer s OR Pim Check OR priscus OR medication appropriateness index OR FORTA OR mcleod* OR zhan OR ACOVE OR pim list* OR pim criteria OR mai).tw,kf.
35	Potentially inappropriate medication list/
36	((record* OR medicine* OR chart* OR drug OR drugs OR dose* OR dosage* OR utilisation OR utilization OR usage OR medication* OR discharge OR prescri* OR appropriateness) adj3 (monitor* OR management OR reconcil* OR review* OR evaluat* OR strategies OR strategy OR evidence base* OR EBM OR assess*)).tw,kf.
37	Medication reconciliation/ OR medication therapy management/ OR Drug utilization review/ OR Drug monitoring/
38	cost control/ OR cost savings/ OR cost benefit analysis/
39	((cost OR costs) adj2 (control OR savings OR analysis OR effective*)).tw,kf.
40	((reduce* OR reducing OR decrease* OR decreasing OR modif* OR alter* OR adapt* OR change* OR changing OR curtail* OR revise* OR revising OR revision OR adjust* OR deescalate* OR de escalate* OR lower* OR fewer OR minimize* OR minimise* OR minimizing OR minimising OR eliminating OR eliminate* OR elimination OR cessation OR cease* OR ceasing OR withheld OR withhold* OR withdraw* OR withdrew OR stop* OR review* OR curb* OR evaluat* OR assess* OR monitor*) adj3 (intermittent OR on demand OR usage OR dosage* OR dose* OR dosing OR medication* OR medicine* OR prescri* OR drug OR drugs OR regimen* OR cost OR costs OR error* OR rate* OR polypharm* OR poly pharm* OR polymedicat* OR poly medicat* OR long term OR longterm OR duration OR PIM OR PIMs OR pim s OR PPI OR PPIS OR ppi s OR sup OR ast OR asm OR asms OR asm s OR inappropriate* OR nonappropriate* OR non appropriate* OR overprescri* OR over prescri* OR overmedicat* OR over medicat* OR overus* OR mistreat* OR overtreat* OR overutili* OR over utili* OR improper* OR incorrect* OR unwarranted OR unjustified OR erroneous* OR misus* OR un necessar* OR unnecessar* OR utilization OR utilisation OR practice pattern* OR “over use” OR “use”)).tw,kf.

41	((proton pump inhibitor* OR ppi OR ppis OR ppi s OR abeprazan OR benatoprazole OR tenatoprazole OR tu 199 OR cas 113712-98-4 OR stu-na OR esomeprazole OR esotrex OR alenia OR escz OR esofag OR nexiam OR ilaprazole OR lansoprazole OR lanzoprazole OR agopton OR bamalite OR inhibitol OR levant OR lupizole OR lanzor OR monolitum OR ogast OR ogastro OR opiren OR prevacid OR prezal OR pro ulco OR promeco OR takepron OR ulpax OR zoton OR dexlansoprazole OR kapidex OR dexilant OR levolansoprazole OR leminoprazole OR linaprazan OR nepaprazole OR omeprazole OR losec OR nexium OR prilosec OR rapinex OR zegerid OR ocid OR lomac OR omepral OR omez OR pantoprazole OR protium OR protonix OR pantotab OR pantopan OR pantozol OR pantor OR pantoloc OR astropan OR controloc OR pantecta OR inipomp OR somac OR pantodac OR zurcal OR zentro OR picoprazole OR potassium competitive acid blocker OR pumaprazole OR rabeprazole OR aciphex OR dexrabeprazole OR pariet OR zechin OR rabecid OR nzole-d OR rabeloc OR revaprazan OR saviprazole OR soraprazan OR tegoprazan OR timoprazole OR vonoprazan OR antiulcer agent* OR antiulcer drug* OR antiulcer medication* OR antiulcer* medicine* OR antiulcer treatment* OR antiulcer* therap* OR anti ulcer* agent* OR anti ulcer* drug* OR anti ulcer* medication* OR anti ulcer* medicine* OR anti ulcer* treatment* OR anti ulcer* therap* OR gastrointestinal agent* OR gastrointestinal drug* OR gastrointestinal medication* OR gastrointestinal medicine* OR gastro intestinal agent* OR gastro intestinal drug* OR gastro intestinal medication* OR gastro intestinal medicine* OR antireflux agent* OR antireflux drug* OR antireflux medication* OR antireflux medicine* OR antireflux treatment* OR antireflux therap* OR anti reflux agent* OR anti reflux drug* OR anti reflux medication* OR anti reflux medicine* OR anti reflux treatment* OR anti reflux therap* OR gastroesophageal agent* OR gastroesophageal drug* OR gastroesophageal medication* OR gastroesophageal medicine* OR gastroesophageal treatment* OR gastroesophageal therap* OR gastro esophageal agent* OR gastro esophageal drug* OR gastro esophageal medication* OR gastro esophageal medicine* OR gastro esophageal treatment* OR gastro esophageal therap* OR gastroprotect* agent* OR gastroprotect* drug* OR gastroprotect* medication* OR gastroprotect* medicine* OR gastroprotect* treatment* OR gastroprotect* therap* OR gastro protect* agent* OR gastro protect* drug* OR gastro protect* medication* OR

	gastro protect* medicine* OR gastro protect* treatment* OR gastro protect* therap* OR gastrointestinal prophylaxis OR gastro intestinal prophylaxis OR peptic ulcer prophylaxis OR stress ulcer prophylaxis OR stress ulcer prophylactic OR acid suppressive OR acid suppression OR acid suppressant* OR acid suppressing OR acid suppressor* OR antacid*) adj3 (reduce* OR reducing OR decrease* OR decreasing OR modif* OR alter* OR change* OR changing OR curtail* OR revise* OR revising OR revision OR adjust* OR deescalate* OR de escalate* OR lower* OR fewer OR minimize* OR minimise* OR minimizing OR minimising OR eliminate* OR elimination OR cessation OR cease* OR ceasing OR withheld OR withhold* OR withdraw* OR withdrew OR stop* OR monitor* OR review* OR curb* OR evaluat* OR assess* OR manage*)).tw,kf.
42	OR/26-41
43	25 AND 42
44	exp animals/ NOT human/
45	43 NOT 44

	Cochrane CENTRAL (EBSCO)
1	**TI** (inappropriate* OR nonappropriate* OR “non appropriate*” OR nonindicat* OR “non indicat*” OR (without N4 indication*) OR polypharm* OR “poly pharm*” OR polymedicat* OR “poly medicat*” OR overprescri* OR “over prescri*” OR overmedicat* OR “over medicat*” OR overus* OR “over use” OR mistreat* OR overtreat* OR overutili* OR “over utili*” OR improper* OR incorrect* OR unwarranted OR unjustified OR erroneous* OR misus* OR “un necessar*” OR unnecessar* OR “sub optimal” OR “repeat* prescri*” OR “repeat* medicine*” OR “repeat* medication*” OR irrational* OR expensive OR expense* OR expenditure* OR “drug related problem*” OR “medication related problem*” OR indiscriminate* OR discrepanc* OR nonguideline* OR “non guideline*” OR questionable OR omission* OR unlicense* OR unapprov* OR “un approv*” OR “dispens* error*” OR wrong* OR “near miss” OR “utilization* pattern*” OR “utilisation* pattern*” OR “practice pattern*” OR “multiple medication*” OR “non beneficial” OR nonbeneficial OR unsafe*) OR **AB** (inappropriate* OR nonappropriate* OR “non appropriate*” OR nonindicat* OR “non indicat*” OR (without N4 indication*) OR polypharm* OR “poly pharm*” OR polymedicat* OR “poly medicat*” OR overprescri* OR “over prescri*” OR overmedicat* OR “over medicat*” OR overus* OR “over use” OR mistreat* OR overtreat* OR overutili* OR “over utili*” OR improper* OR incorrect* OR unwarranted OR unjustified OR erroneous* OR misus* OR “un necessar*” OR unnecessar* OR “sub optimal” OR “repeat* prescri*” OR “repeat* medicine*” OR “repeat* medication*” OR irrational* OR expensive OR expense* OR expenditure* OR “drug related problem*” OR “medication related problem*” OR indiscriminate* OR discrepanc* OR nonguideline* OR “non guideline*” OR questionable OR omission* OR unlicense* OR unapprov* OR “un approv*” OR “dispens* error*” OR wrong* OR “near miss” OR “utilization* pattern*” OR “utilisation* pattern*” OR “practice pattern*” OR “multiple medication*” OR “non beneficial” OR nonbeneficial OR unsafe*)
2	**TI** ((improve* OR improving OR optimize* OR optimizing OR optimise* OR optimising) N3 (safety OR usage OR use OR prescri*)) OR **AB** ((improve* OR improving OR optimize* OR optimizing OR optimise* OR optimising) N3 (safety OR usage OR use OR prescri*))

3	**TI** ((cost OR costs OR costly OR costing) N3 (high* OR increase* OR grow* OR financial OR economic OR drug OR drugs OR prescri* OR medicine* OR medication* OR dose* OR dosing OR dosage* OR therap* OR treat* OR use OR usage)) OR **AB** ((cost OR costs OR costly OR costing) N3 (high* OR increase* OR grow* OR financial OR economic OR drug OR drugs OR prescri* OR medicine* OR medication* OR dose* OR dosing OR dosage* OR therap* OR treat* OR use OR usage))
4	**TI** (prescri* N3 (pattern* OR habit* OR practice* OR regimen* OR trend* OR behavior* OR behaviour* OR cascade* OR record* OR indication*)) OR **AB** (prescri* N3 (pattern* OR habit* OR practice* OR regimen* OR trend* OR behavior* OR behaviour* OR cascade* OR record* OR indication*))
5	**TI** (reconcil* N3 error*) OR **AB** (reconcil* N3 error*)
6	**TI** (safe* N3 prescri*) OR **AB** (safe* N3 prescri*)
7	**TI** ((usage OR use OR dosage* OR dose* OR dosing OR medication* OR medicine* OR prescri* OR drug OR drugs OR therap* OR treat* OR indication* OR cost OR costs OR costly OR costing OR dispens*) N3 (appropriate* OR utilization* OR utilisation* OR justif* OR error* OR inadequate* OR excess* OR suboptimal* OR inadvertent* OR rational* OR harmful OR “long term” OR longterm OR optimal OR reasonable OR reasonably)) OR **AB** ((usage OR use OR dosage* OR dose* OR dosing OR medication* OR medicine* OR prescri* OR drug OR drugs OR therap* OR treat* OR indication* OR cost OR costs OR costly OR costing OR dispens*) N3 (appropriate* OR utilization* OR utilisation* OR justif* OR error* OR inadequate* OR excess* OR suboptimal* OR inadvertent* OR rational* OR harmful OR “long term” OR longterm OR optimal OR reasonable OR reasonably))
8	**TI** (risk* N3 (usage OR use OR medication* OR medicine* OR prescri*)) OR **AB** (risk* N3 (usage OR use OR medication* OR medicine* OR prescri*))
9	**TI** ((nonadhere* OR “non adhere*” OR noncompliance OR noncompliant OR “non compliance” OR “non compliant”) N3 (guideline* OR indication*

	OR prescri* OR protocol* OR policy OR policies OR regulation*)) OR **AB** ((nonadhere* OR “non adhere*” OR noncompliance OR noncompliant OR “non compliance” OR “non compliant”) N3 (guideline* OR indication* OR prescri* OR protocol* OR policy OR policies OR regulation*))
10	**TI** ((valid* OR accura* OR approve*) N3 (prescri* OR indication*)) OR **AB** ((valid* OR accura* OR approve*) N3 (prescri* OR indication*))
11	MH Inappropriate Prescribing OR MH Polypharmacy OR MH Drug Misuse OR MH Prescription Drug Misuse OR MH Prescription Drug Overuse OR MH Practice Patterns, Nurses' OR MH Practice Patterns, Physicians' OR MH Medication Errors OR MH Medical Errors OR MH Long-Term Care OR MH Drug Utilization
12	OR/1-11
13	**TI** (“proton pump inhibitor*” OR abeprazan OR benatoprazole OR tenatoprazole OR “STU-Na” OR esomeprazole OR esotrex OR alenia OR escz OR esofag OR nexiam OR ilaprazole OR lansoprazole OR lanzoprazole OR agopton OR bamalite OR inhibitol OR levant OR lupizole OR lanzor OR monolitum OR ogast OR ogastro OR opiren OR prevacid OR prezal OR “pro ulco” OR promeco OR takepron OR ulpax OR zoton OR dexlansoprazole OR kapidex OR dexilant OR levolansoprazole OR leminoprazole OR linaprazan OR nepaprazole OR omeprazole OR losec OR nexium OR prilosec OR rapinex OR zegerid OR ocid OR lomac OR omepral OR omez OR pantoprazole OR protium OR protonix OR pantotab OR pantopan OR pantozol OR pantor OR pantoloc OR astropan OR controloc OR pantecta OR inipomp OR somac OR pantodac OR zurcal OR zentro OR picoprazole OR “potassium competitive acid blocker” OR pumaprazole OR rabeprazole OR aciphex OR dexrabeprazole OR pariet OR zechin OR rabecid OR “nzole-d” OR rabeloc OR revaprazan OR saviprazole OR soraprazan OR tegoprazan OR timoprazole OR vonoprazan) OR **AB** (“proton pump inhibitor*” OR abeprazan OR benatoprazole OR tenatoprazole OR “STU-Na” OR esomeprazole OR esotrex OR alenia OR escz OR esofag OR nexiam OR ilaprazole OR lansoprazole OR lanzoprazole OR agopton OR bamalite OR inhibitol OR levant OR lupizole OR lanzor OR monolitum OR ogast OR ogastro OR opiren OR prevacid OR prezal OR “pro ulco” OR promeco OR takepron OR ulpax OR zoton OR dexlansoprazole OR kapidex OR dexilant

	OR levolansoprazole OR leminoprazole OR linaprazan OR nepaprazole OR omeprazole OR losec OR nexium OR prilosec OR rapinex OR zegerid OR ocid OR lomac OR omepral OR omez OR pantoprazole OR protium OR protonix OR pantotab OR pantopan OR pantozol OR pantor OR pantoloc OR astropan OR controloc OR pantecta OR inipomp OR somac OR pantodac OR zurcal OR zentro OR picoprazole OR "potassium competitive acid blocker" OR pumaprazole OR rabeprazole OR aciphex OR dexrabeprazole OR pariet OR zechin OR rabecid OR "nzole-d" OR rabeloc OR revaprazan OR saviprazole OR soraprazan OR tegoprazan OR timoprazole OR vonoprazan)
14	**TI** (proton N2 pump N2 inhibitor*) OR **AB** (proton N2 pump N2 inhibitor*)
15	**TI** ((antiulcer OR gastrointestinal OR "gastro intestinal" OR antireflux OR gastroesophageal OR "gastro esophageal" OR gastroprotect* OR "gastro protect*") N2 (agent* OR drug OR drugs OR medication* OR medicine*)) OR **AB** ((antiulcer OR gastrointestinal OR "gastro intestinal" OR antireflux OR gastroesophageal OR "gastro esophageal" OR gastroprotect* OR "gastro protect*") N2 (agent* OR drug OR drugs OR medication* OR medicine*))
16	**TI** (anti N2 (ulcer OR reflux) N2 (agent* OR drug OR drugs OR medication* OR medicine*)) OR **AB** (anti N2 (ulcer OR reflux) N2 (agent* OR drug OR drugs OR medication* OR medicine*))
17	**TI** ((peptic OR stress) N2 ulcer N2 (prophylaxis OR prophylactic)) OR **AB** ((peptic OR stress) N2 ulcer N2 (prophylaxis OR prophylactic))
18	**TI** ((gastrointestinal OR "gastro intestinal") N2 (prophylaxis OR prophylactic)) OR **AB** ((gastrointestinal OR "gastro intestinal") N2 (prophylaxis OR prophylactic))
19	**TI** ((gastric OR acid) N2 (suppressive OR suppression OR suppressant* OR suppressing OR suppressor*)) OR **AB** ((gastric OR acid*) N2 (suppressive OR suppression OR suppressant* OR suppressing OR suppressor*))
20	**TI** antacid* OR **AB** antacid*

21	MH Proton Pump Inhibitors OR MH Dexlansoprazole OR MH Esomeprazole OR MH Lansoprazole OR MH Omeprazole OR MH Rabeprazole OR MH Anti-Ulcer Agents OR MH Gastrointestinal Agents OR MH Antacids
22	OR/13-21
23	12 AND 22
24	**TI** ((“proton pump inhibitor*” OR ppi OR ppis OR “ppi’s” OR abeprazan OR benatoprazole OR tenatoprazole OR “stu-na” OR esomeprazole OR esotrex OR alenia OR escz OR esofag OR nexiam OR ilaprazole OR lansoprazole OR lanzoprazole OR agopton OR bamalite OR inhibitol OR levant OR lupizole OR lanzor OR monolitum OR ogast OR ogastro OR opiren OR prevacid OR prezal OR “pro ulco” OR promeco OR takepron OR ulpax OR zoton OR dexlansoprazole OR kapidex OR dexilant OR levolansoprazole OR leminoprazole OR linaprazan OR nepaprazole OR omeprazole OR losec OR nexium OR prilosec OR rapinex OR zegerid OR ocid OR lomac OR omepral OR omez OR pantoprazole OR protium OR protonix OR pantotab OR pantopan OR pantozol OR pantor OR pantoloc OR astropan OR controloc OR pantecta OR inipomp OR somac OR pantodac OR zurcal OR zentro OR picoprazole OR “potassium competitive acid blocker” OR pumaprazole OR rabeprazole OR aciphex OR dexrabeprazole OR pariet OR zechin OR rabecid OR “nzole-d” OR rabeloc OR revaprazan OR saviprazole OR soraprazan OR tegoprazan OR timoprazole OR vonoprazan OR “antiulcer agent*” OR “antiulcer drug*” OR “antiulcer medication*” OR “antiulcer* medicine*” OR “antiulcer treatment*” OR “antiulcer* therap*” OR “anti ulcer* agent*” OR “anti ulcer* drug*” OR “anti ulcer* medication*” OR “anti ulcer* medicine*” OR “anti ulcer* treatment*” OR “anti ulcer* therap*” OR “gastrointestinal agent*” OR “gastrointestinal drug*” OR “gastrointestinal medication*” OR “gastrointestinal medicine*” OR “gastro intestinal agent*” OR “gastro intestinal drug*” OR “gastro intestinal medication*” OR “gastro intestinal medicine*” OR “antireflux agent*” OR “antireflux drug*” OR “antireflux medication*” OR “antireflux medicine*” OR “antireflux treatment*” OR “antireflux therap*” OR “anti reflux agent*” OR “anti reflux drug*” OR “anti reflux medication*” OR “anti reflux medicine*” OR “anti reflux treatment*” OR “anti reflux therap*” OR “gastroesophageal agent*” OR “gastroesophageal drug*” OR “gastroesophageal medication*” OR “gastroesophageal medicine*” OR

	"gastroesophageal treatment*" OR "gastroesophageal therap*" OR "gastro esophageal agent*" OR "gastro esophageal drug*" OR "gastro esophageal medication*" OR "gastro esophageal medicine*" OR "gastro esophageal treatment*" OR "gastro esophageal therap*" OR "gastroprotect* agent*" OR "gastroprotect* drug*" OR "gastroprotect* medication*" OR "gastroprotect* medicine*" OR "gastroprotect* treatment*" OR "gastroprotect* therap*" OR "gastro protect* agent*" OR "gastro protect* drug*" OR "gastro protect* medication*" OR "gastro protect* medicine*" OR "gastro protect* treatment*" OR "gastro protect* therap*" OR "gastrointestinal prophylaxis" OR "gastro intestinal prophylaxis" OR "peptic ulcer prophylaxis" OR "stress ulcer prophylaxis" OR "stress ulcer prophylactic" OR "acid suppressive" OR "acid suppression" OR "acid suppressant*" OR "acid suppressing" OR "acid suppressor*" OR antacid*) N3 (appropriate* OR "no indication*" OR utilization* OR utilisation* OR justif* OR error* OR inadequate* OR excess* OR suboptimal* OR inadvertent* OR rational* OR harmful OR "long term" OR longterm OR risk* OR adverse OR optimal OR reasonable OR reasonably OR cost OR costs OR costly OR costing)) OR **AB** (("proton pump inhibitor*" OR ppi OR ppis OR "ppi s" OR abeprazan OR benatoprazole OR tenatoprazole OR "stu-na" OR esomeprazole OR esotrex OR alenia OR escz OR esofag OR nexiam OR ilaprazole OR lansoprazole OR lanzoprazole OR agopton OR bamalite OR inhibitol OR levant OR lupizole OR lanzor OR monolitum OR ogast OR ogastro OR opiren OR prevacid OR prezal OR "pro ulco" OR promeco OR takepron OR ulpax OR zoton OR dexlansoprazole OR kapidex OR dexilant OR levolansoprazole OR leminoprazole OR linaprazan OR nepaprazole OR omeprazole OR losec OR nexium OR prilosec OR rapinex OR zegerid OR ocid OR lomac OR omepral OR omez OR pantoprazole OR protium OR protonix OR pantotab OR pantopan OR pantozol OR pantor OR pantoloc OR astropan OR controloc OR pantecta OR inipomp OR somac OR pantodac OR zurcal OR zentro OR picoprazole OR "potassium competitive acid blocker" OR pumaprazole OR rabeprazole OR aciphex OR dexrabeprazole OR pariet OR zechin OR rabecid OR "nzole-d" OR rabeloc OR revaprazan OR saviprazole OR soraprazan OR tegoprazan OR timoprazole OR vonoprazan OR "antiulcer agent*" OR "antiulcer drug*" OR "antiulcer medication*" OR "antiulcer* medicine*" OR "antiulcer treatment*" OR "antiulcer* therap*" OR "anti ulcer* agent*" OR "anti ulcer* drug*" OR "anti ulcer* medication*" OR

	“anti ulcer* medicine*” OR “anti ulcer* treatment*” OR “anti ulcer* therap*” OR “gastrointestinal agent*” OR “gastrointestinal drug*” OR “gastrointestinal medication*” OR “gastrointestinal medicine*” OR “gastro intestinal agent*” OR “gastro intestinal drug*” OR “gastro intestinal medication*” OR “gastro intestinal medicine*” OR “antireflux agent*” OR “antireflux drug*” OR “antireflux medication*” OR “antireflux medicine*” OR “antireflux treatment*” OR “antireflux therap*” OR “anti reflux agent*” OR “anti reflux drug*” OR “anti reflux medication*” OR “anti reflux medicine*” OR “anti reflux treatment*” OR “anti reflux therap*” OR “gastroesophageal agent*” OR “gastroesophageal drug*” OR “gastroesophageal medication*” OR “gastroesophageal medicine*” OR “gastroesophageal treatment*” OR “gastroesophageal therap*” OR “gastro esophageal agent*” OR “gastro esophageal drug*” OR “gastro esophageal medication*” OR “gastro esophageal medicine*” OR “gastro esophageal treatment*” OR “gastro esophageal therap*” OR “gastroprotect* agent*” OR “gastroprotect* drug*” OR “gastroprotect* medication*” OR “gastroprotect* medicine*” OR “gastroprotect* treatment*” OR “gastroprotect* therap*” OR “gastro protect* agent*” OR “gastro protect* drug*” OR “gastro protect* medication*” OR “gastro protect* medicine*” OR “gastro protect* treatment*” OR “gastro protect* therap*” OR “gastrointestinal prophylaxis” OR “gastro intestinal prophylaxis” OR “peptic ulcer prophylaxis” OR “stress ulcer prophylaxis” OR “stress ulcer prophylactic” OR “acid suppressive” OR “acid suppression” OR “acid suppressant*” OR “acid suppressing” OR “acid suppressor*” OR antacid*) N3 (appropriate* OR “no indication*” OR utilization* OR utilisation* OR justif* OR error* OR inadequate* OR excess* OR suboptimal* OR inadvertent* OR rational* OR harmful OR “long term” OR longterm OR risk* OR adverse OR optimal OR reasonable OR reasonably OR cost OR costs OR costly OR costing))
25	23 OR 24
26	**TI** (deprescri* OR “de prescri*” OR unprescri* OR “un prescri*” OR discontinu* OR reduction* OR “step* down” OR stepwise OR “step wise” OR “step off” OR compliance OR adherence OR audit OR audits OR taper* OR benchmark* OR “bench mark*” OR TQM OR “academic detailing”) OR **AB** (deprescri* OR “de prescri*” OR unprescri* OR “un prescri*” OR discontinu* OR reduction* OR “step* down” OR stepwise OR “step wise”

	OR “step off” OR compliance OR adherence OR audit OR audits OR taper* OR benchmark* OR “bench mark*” OR TQM OR “academic detailing”)
27	MH Total Quality Management OR MH Quality Control OR MH Quality Improvement OR MH Quality Assurance, Health Care
28	**TI** (Quality n1 (control OR improvement* OR assurance OR assess* OR review* OR prescri* OR guideline* OR protocol* OR standard* OR policy OR policies OR regulation* OR management OR intervention OR strategy)) OR **AB** (Quality n1 (control OR improvement* OR assurance OR assess* OR review* OR prescri* OR guideline* OR protocol* OR standard* OR policy OR policies OR regulation* OR management OR intervention OR strategy))
29	MH Benchmarking OR MH management audit OR MH Medical audit OR MH Clinical audit OR MH deprescriptions OR MH patient compliance OR MH medication adherence OR MH guideline adherence OR MH practice guideline OR MH withholding treatment OR MH “treatment adherence and compliance”
30	**TI** ((Protocol* OR Guideline* OR policy OR policies OR regulation*) n3 (compliance OR adhere* OR implement* OR evaluat* OR comply OR compliant OR concord* OR distribut* OR disseminat* OR “best practice*” OR effective* OR “evidence base*” OR EBM OR introduce* OR issu* OR impact* OR adhering OR complying OR assess* OR review*)) OR **AB** ((Protocol* OR Guideline* OR policy OR policies OR regulation*) n3 (compliance OR adhere* OR implement* OR evaluat* OR comply OR compliant OR concord* OR distribut* OR disseminat* OR “best practice*” OR effective* OR “evidence base*” OR EBM OR introduce* OR issu* OR impact* OR adhering OR complying OR assess* OR review*))
31	MH Pharmacists OR MH Pharmacy Technicians
32	**TI** (Pharmacy OR Pharmacies OR pharmacotherapeutic* OR pharmd OR pharmacist*) OR **AB** (pharmacy OR pharmacies OR pharmacotherapeutic* OR pharmd OR pharmacist*)

33	**TI** (pharmaceutical n2 (care OR service* OR consult* OR advice OR advise* OR advising OR intervention)) OR **AB** (pharmaceutical n2 (care OR service* OR consult* OR advice OR advise* OR advising OR intervention))
34	MH Students, Pharmacy OR MH Education, Pharmacy, Continuing OR MH Pharmacy Service, Hospital OR MH Community Pharmacy Services OR MH Pharmacy OR MH "Pharmacy and Therapeutics Committee" OR MH Pharmaceutical Services
35	**TI** ("Screening test*" OR "screening tool*" OR "systematic tool*" OR Stopp OR "start criteria" OR "beers" OR "beer's" OR "Pim Check" OR priscus OR "medication appropriateness index" OR FORTA OR mcleod* OR zhan OR ACOVE OR "pim list*" OR "pim criteria"OR mai) OR **AB** ("Screening test*" OR "screening tool*" OR "systematic tool*" OR Stopp OR "start criteria" OR "beers" OR "beer's" OR "Pim Check" OR priscus OR "medication appropriateness index" OR FORTA OR mcleod* OR zhan OR ACOVE OR "pim list*" OR "pim criteria"OR mai)
36	MH Potentially inappropriate medication list
37	**TI** ((record* OR medicine* OR chart* OR drug* OR dose* OR dosage* OR utilisation OR utilization OR usage OR medication* OR discharge OR prescri* OR appropriateness) N3 (monitor* OR management OR reconcil* OR review* OR evaluat* OR strategies OR strategy OR "evidence base*" OR EBM)) OR **AB** ((record* OR medicine* OR chart* OR drug* OR dose* OR dosage* OR utilisation OR utilization OR usage OR medication* OR discharge OR prescri* OR appropriateness) N3 (monitor* OR management OR reconcile* OR review* OR evaluat* OR strategies OR strategy OR "evidence base*" OR EBM))
38	MH Medication reconciliation OR MH medication therapy management OR MH Drug utilization review
39	MH cost control OR MH cost savings OR MH cost benefit analysis
41	**TI** ((cost OR costs) n2 (control OR savings OR analysis OR effective*)) OR **AB** ((cost OR costs) n2 (control OR savings OR analysis OR effective*))

42	**TI** ((reduce* OR reducing OR decrease* OR decreasing OR modif* OR alter* OR adapt* OR change* OR changing OR curtail* OR revise* OR revising OR revision OR adjust* OR deescalate* OR “de escalate*” OR lower* OR fewer OR minimize* OR minimise* OR minimizing OR minimising OR eliminating OR eliminate* OR elimination OR cessation OR cease* OR ceasing OR withheld OR withhold* OR withdraw* OR withdrew OR stop* OR review* OR curb* OR evaluat* OR assess* OR monitor*) n3 (intermittent OR usage OR use OR dosage* OR dose* OR dosing OR medication* OR medicine* OR prescri* OR drug OR drugs OR “regimen* OR cost OR costs OR error* OR rate* OR polypharm* OR “poly pharm*” OR polymedicat* OR “poly medicat*” OR “long term” OR longterm OR duration OR PIM OR PIMs OR “pim’s” OR PPI OR PPIS OR “ppi’s” OR sup OR ast OR asm OR asms OR “asm’s” OR inappropriate* OR nonappropriat* OR “non appropriat*” OR overprescri* OR “over prescri*” OR overmedicat* OR “over medicat*” OR overus* OR “over use” OR mistreat* OR overtreat* OR overutili* OR “over utili*” OR improper* OR incorrect* OR unwarranted OR unjustified OR erroneous* OR misus* OR “un necessar*” OR unnecessar* OR utilization OR utilisation OR “practice pattern*”)) OR **AB** ((reduce* OR reducing OR decrease* OR decreasing OR modif* OR alter* OR adapt* OR change* OR changing OR curtail* OR revise* OR revising OR revision OR adjust* OR deescalate* OR “de escalate*” OR lower* OR fewer OR minimize* OR minimise* OR minimizing OR minimising OR eliminating OR eliminate* OR elimination OR cessation OR cease* OR ceasing OR withheld OR withhold* OR withdraw* OR withdrew OR stop* OR review* OR curb* OR evaluat* OR assess* OR monitor*) n3 (intermittent OR usage OR use OR dosage* OR dose* OR dosing OR medication* OR medicine* OR prescri* OR drug OR drugs OR regimen* OR cost OR costs OR error* OR rate* OR polypharm* OR “poly pharm*” OR polymedicat* OR “poly medicat*” OR “long term” OR longterm OR duration OR PIM OR PIMs OR “pim’s” OR PPI OR PPIS OR “ppi’s” OR sup OR ast OR asm OR asms OR “asm’s” OR inappropriate* OR nonappropriat* OR “non appropriat*” OR overprescri* OR “over prescri*” OR overmedicat* OR “over medicat*” OR overus* OR “over use” OR mistreat* OR overtreat* OR overutili* OR “over utili*” OR improper* OR incorrect* OR unwarranted OR unjustified OR erroneous* OR misus* OR “un necessar*” OR unnecessar* OR utilization OR utilisation OR “practice pattern*”))

43	MH Drug monitoring
44	**TI** ((“proton pump inhibitor*” OR ppi OR ppis OR “ppi’s” OR abeprazan OR benatoprazole OR tenatoprazole OR “stu-na” OR esomeprazole OR esotrex OR alenia OR escz OR esofag OR nexiam OR ilaprazole OR lansoprazole OR lanzoprazole OR agopton OR bamalite OR inhibitol OR levant OR lupizole OR lanzor OR monolitum OR ogast OR ogastro OR opiren OR prevacid OR prezal OR “pro ulco” OR promeco OR takepron OR ulpax OR zoton OR dexlansoprazole OR kapidex OR dexilant OR levolansoprazole OR leminoprazole OR linaprazan OR nepaprazole OR omeprazole OR losec OR nexium OR prilosec OR rapinex OR zegerid OR ocid OR lomac OR omepral OR omez OR pantoprazole OR protium OR protonix OR pantotab OR pantopan OR pantozol OR pantor OR pantoloc OR astropan OR controloc OR pantecta OR inipomp OR somac OR pantodac OR zurcal OR zentro OR picoprazole OR “potassium competitive acid blocker” OR pumaprazole OR rabeprazole OR aciphex OR dexrabeprazole OR pariet OR zechin OR rabecid OR “nzole-d” OR rabeloc OR revaprazan OR saviprazole OR soraprazan OR tegoprazan OR timoprazole OR vonoprazan OR “antiulcer agent*” OR “antiulcer drug*” OR “antiulcer medication*” OR “antiulcer* medicine*” OR “antiulcer treatment*” OR “antiulcer* therap*” OR “anti ulcer* agent*” OR “anti ulcer* drug*” OR “anti ulcer* medication*” OR “anti ulcer* medicine*” OR “anti ulcer* treatment*” OR “anti ulcer* therap*” OR “gastrointestinal agent*” OR “gastrointestinal drug*” OR “gastrointestinal medication*” OR “gastrointestinal medicine*” OR “gastro intestinal agent*” OR “gastro intestinal drug*” OR “gastro intestinal medication*” OR “gastro intestinal medicine*” OR “antireflux agent*” OR “antireflux drug*” OR “antireflux medication*” OR “antireflux medicine*” OR “antireflux treatment*” OR “antireflux therap*” OR “anti reflux agent*” OR “anti reflux drug*” OR “anti reflux medication*” OR “anti reflux medicine*” OR “anti reflux treatment*” OR “anti reflux therap*” OR “gastroesophageal agent*” OR “gastroesophageal drug*” OR “gastroesophageal medication*” OR “gastroesophageal medicine*” OR “gastroesophageal treatment*” OR “gastroesophageal therap*” OR “gastro esophageal agent*” OR “gastro esophageal drug*” OR “gastro esophageal medication*” OR “gastro esophageal medicine*” OR “gastro esophageal treatment*” OR “gastro esophageal therap*” OR “gastroprotect* agent*” OR “gastroprotect* drug*” OR “gastroprotect*

	medication*" OR "gastroprotect* medicine*" OR "gastroprotect* treatment*" OR "gastroprotect* therap*" OR "gastro protect* agent*" OR "gastro protect* drug*" OR "gastro protect* medication*" OR "gastro protect* medicine*" OR "gastro protect* treatment*" OR "gastro protect* therap*" OR "gastrointestinal prophylaxis" OR "gastro intestinal prophylaxis" OR "peptic ulcer prophylaxis" OR "stress ulcer prophylaxis" OR "stress ulcer prophylactic" OR "acid suppressive" OR "acid suppression" OR "acid suppressant*" OR "acid suppressing" OR "acid suppressor*" OR antacid*) N3 (reduce* OR reducing OR decrease* OR decreasing OR modif* OR alter* OR change* OR changing OR curtail* OR revise* OR revising OR revision OR adjust* OR deescalate* OR "de escalate*" OR lower* OR fewer OR minimize* OR minimise* OR minimizing OR minimising OR eliminate* OR elimination OR cessation OR cease* OR ceasing OR withheld OR withhold* OR withdraw* OR withdrew OR stop* OR monitor* OR review* OR curb* OR evaluat* OR assess*)) OR **AB** (("proton pump inhibitor*" OR ppi OR ppis OR "ppi"s" OR abeprazan OR benatoprazole OR tenatoprazole OR "stu-na" OR esomeprazole OR esotrex OR alenia OR escz OR esofag OR nexiam OR ilaprazole OR lansoprazole OR lanzoprazole OR agopton OR bamalite OR inhibitol OR levant OR lupizole OR lanzor OR monolitum OR ogast OR ogastro OR opiren OR prevacid OR prezal OR "pro ulco" OR promeco OR takepron OR ulpax OR zoton OR dexlansoprazole OR kapidex OR dexilant OR levolansoprazole OR leminoprazole OR linaprazan OR nepaprazole OR omeprazole OR losec OR nexium OR prilosec OR rapinex OR zegerid OR ocid OR lomac OR omepral OR omez OR pantoprazole OR protium OR protonix OR pantotab OR pantopan OR pantozol OR pantor OR pantoloc OR astropan OR controloc OR pantecta OR inipomp OR somac OR pantodac OR zurcal OR zentro OR picoprazole OR "potassium competitive acid blocker" OR pumaprazole OR rabeprazole OR aciphex OR dexrabeprazole OR pariet OR zechin OR rabecid OR "nzole-d" OR rabeloc OR revaprazan OR saviprazole OR soraprazan OR tegoprazan OR timoprazole OR vonoprazan OR "antiulcer agent*" OR "antiulcer drug*" OR "antiulcer medication*" OR "antiulcer* medicine*" OR "antiulcer treatment*" OR "antiulcer* therap*" OR "anti ulcer* agent*" OR "anti ulcer* drug*" OR "anti ulcer* medication*" OR "anti ulcer* medicine*" OR "anti ulcer* treatment*" OR "anti ulcer* therap*" OR "gastrointestinal agent*" OR "gastrointestinal drug*" OR "gastrointestinal medication*" OR

	“gastrointestinal medicine*” OR “gastro intestinal agent*” OR “gastro intestinal drug*” OR “gastro intestinal medication*” OR “gastro intestinal medicine*” OR “antireflux agent*” OR “antireflux drug*” OR “antireflux medication*” OR “antireflux medicine*” OR “antireflux treatment*” OR “antireflux therap*” OR “anti reflux agent*” OR “anti reflux drug*” OR “anti reflux medication*” OR “anti reflux medicine*” OR “anti reflux treatment*” OR “anti reflux therap*” OR “gastroesophageal agent*” OR “gastroesophageal drug*” OR “gastroesophageal medication*” OR “gastroesophageal medicine*” OR “gastroesophageal treatment*” OR “gastroesophageal therap*” OR “gastro esophageal agent*” OR “gastro esophageal drug*” OR “gastro esophageal medication*” OR “gastro esophageal medicine*” OR “gastro esophageal treatment*” OR “gastro esophageal therap*” OR “gastroprotect* agent*” OR “gastroprotect* drug*” OR “gastroprotect* medication*” OR “gastroprotect* medicine*” OR “gastroprotect* treatment*” OR “gastroprotect* therap*” OR “gastro protect* agent*” OR “gastro protect* drug*” OR “gastro protect* medication*” OR “gastro protect* medicine*” OR “gastro protect* treatment*” OR “gastro protect* therap*” OR “gastrointestinal prophylaxis” OR “gastro intestinal prophylaxis” OR “peptic ulcer prophylaxis” OR “stress ulcer prophylaxis” OR “stress ulcer prophylactic” OR “acid suppressive” OR “acid suppression” OR “acid suppressant*” OR “acid suppressing” OR “acid suppressor*” OR antacid*) N3 (reduce* OR reducing OR decrease* OR decreasing OR modif* OR alter* OR change* OR changing OR curtail* OR revise* OR revising OR revision OR adjust* OR deescalate* OR “de escalate*” OR lower* OR fewer OR minimize* OR minimise* OR minimizing OR minimising OR eliminate* OR elimination OR cessation OR cease* OR ceasing OR withheld OR withhold* OR withdraw* OR withdrew OR stop* OR monitor* OR review* OR curb* OR evaluat* OR assess*))
45	OR/26-44
46	25 AND 45

	CINAHL
1	**TI** (inappropriate* OR nonappropriate* OR “non appropriate*” OR nonindicat* OR “non indicat*” OR (without N4 indication*) OR polypharm* OR “poly pharm*” OR polymedicat* OR “poly medicat*” OR overprescri* OR “over prescri*” OR overmedicat* OR “over medicat*” OR overus* OR “over use” OR mistreat* OR overtreat* OR overutili* OR “over utili*” OR improper* OR incorrect* OR unwarranted OR unjustified OR erroneous* OR misus* OR “un necessar*” OR unnecessar* OR “sub optimal” OR “repeat* prescri*” OR “repeat* medicine*” OR “repeat* medication*” OR irrational* OR expensive OR expense* OR expenditure* OR “drug related problem*” OR “medication related problem*” OR indiscriminate* OR discrepanc* OR nonguideline* OR “non guideline*” OR questionable OR omission* OR unlicense* OR unapprov* OR “un approv*” OR “dispens* error*” OR wrong* OR “near miss” OR “utilization* pattern*” OR “utilisation* pattern*” OR “practice pattern*” OR “multiple medication*” OR “non beneficial” OR nonbeneficial OR unsafe*) OR **AB** (inappropriate* OR nonappropriate* OR “non appropriate*” OR nonindicat* OR “non indicat*” OR (without N4 indication*) OR polypharm* OR “poly pharm*” OR polymedicat* OR “poly medicat*” OR overprescri* OR “over prescri*” OR overmedicat* OR “over medicat*” OR overus* OR “over use” OR mistreat* OR overtreat* OR overutili* OR “over utili*” OR improper* OR incorrect* OR unwarranted OR unjustified OR erroneous* OR misus* OR “un necessar*” OR unnecessar* OR “sub optimal” OR “repeat* prescri*” OR “repeat* medicine*” OR “repeat* medication*” OR irrational* OR expensive OR expense* OR expenditure* OR “drug related problem*” OR “medication related problem*” OR indiscriminate* OR discrepanc* OR nonguideline* OR “non guideline*” OR questionable OR omission* OR unlicense* OR unapprov* OR “un approv*” OR “dispens* error*” OR wrong* OR “near miss” OR “utilization* pattern*” OR “utilisation* pattern*” OR “practice pattern*” OR “multiple medication*” OR “non beneficial” OR nonbeneficial OR unsafe*)
2	**TI** ((improve* OR improving OR optimize* OR optimizing OR optimise* OR optimising) N3 (safety OR usage OR use OR prescri*)) OR **AB** ((improve* OR improving OR optimize* OR optimizing OR optimise* OR optimising) N3 (safety OR usage OR use OR prescri*))

3	**TI** ((cost OR costs OR costly OR costing) N3 (high* OR increase* OR grow* OR financial OR economic OR drug OR drugs OR prescri* OR medicine* OR medication* OR dose* OR dosing OR dosage* OR therap* OR treat* OR use OR usage)) OR **AB** ((cost OR costs OR costly OR costing) N3 (high* OR increase* OR grow* OR financial OR economic OR drug OR drugs OR prescri* OR medicine* OR medication* OR dose* OR dosing OR dosage* OR therap* OR treat* OR use OR usage))
4	**TI** (prescri* N3 (pattern* OR habit* OR practice* OR regimen* OR trend* OR behavior* OR behaviour* OR cascade* OR record* OR indication*)) OR **AB** (prescri* N3 (pattern* OR habit* OR practice* OR regimen* OR trend* OR behavior* OR behaviour* OR cascade* OR record* OR indication*))
5	**TI** (reconcil* N3 error*) OR **AB** (reconcil* N3 error*)
6	**TI** (safe* N3 prescri*) OR **AB** (safe* N3 prescri*)
7	**TI** ((usage OR use OR dosage* OR dose* OR dosing OR medication* OR medicine* OR prescri* OR drug OR drugs OR therap* OR treat* OR indication* OR cost OR costs OR costly OR costing OR dispens*) N3 (appropriate* OR utilization* OR utilisation* OR justif* OR error* OR inadequate* OR excess* OR suboptimal* OR inadvertent* OR rational* OR harmful OR “long term” OR longterm OR optimal OR reasonable OR reasonably)) OR **AB** ((usage OR use OR dosage* OR dose* OR dosing OR medication* OR medicine* OR prescri* OR drug OR drugs OR therap* OR treat* OR indication* OR cost OR costs OR costly OR costing OR dispens*) N3 (appropriate* OR utilization* OR utilisation* OR justif* OR error* OR inadequate* OR excess* OR suboptimal* OR inadvertent* OR rational* OR harmful OR “long term” OR longterm OR optimal OR reasonable OR reasonably))
8	**TI** (risk* N3 (usage OR use OR medication* OR medicine* OR prescri*)) OR **AB** (risk* N3 (usage OR use OR medication* OR medicine* OR prescri*))
9	**TI** ((nonadhere* OR “non adhere*” OR noncompliance OR noncompliant OR “non compliance” OR “non compliant”) N3 (guideline* OR indication* OR prescri* OR protocol* OR policy OR policies OR regulation*)) OR **AB**

	((nonadhere* OR “non adhere*” OR noncompliance OR noncompliant OR “non compliance” OR “non compliant”) N3 (guideline* OR indication* OR prescri* OR protocol* OR policy OR policies OR regulation*))
10	**TI** ((valid* OR accura* OR approve*) N3 (prescri* OR indication*)) OR **AB** ((valid* OR accura* OR approve*) N3 (prescri* OR indication*))
11	MH Prescribing Patterns OR MH Inappropriate Prescribing OR MH Drug Utilization OR MH Practice Patterns OR MH Polypharmacy OR MH Medication Errors+ OR MH Inappropriate Prescribing OR MH Long Term Care
12	OR/1-11
13	**TI** (“proton pump inhibitor*” OR abeprazan OR benatoprazole OR tenatoprazole OR “STU-Na” OR esomeprazole OR esotrex OR alenia OR escz OR esofag OR nexiam OR ilaprazole OR lansoprazole OR lanzoprazole OR agopton OR bamalite OR inhibitol OR levant OR lupizole OR lanzor OR monolitum OR ogast OR ogastro OR opiren OR prevacid OR prezal OR “pro ulco” OR promeco OR takepron OR ulpax OR zoton OR dexlansoprazole OR kapidex OR dexilant OR levolansoprazole OR leminoprazole OR linaprazan OR nepaprazole OR omeprazole OR losec OR nexium OR prilosec OR rapinex OR zegerid OR ocid OR lomac OR omepral OR omez OR pantoprazole OR protium OR protonix OR pantotab OR pantopan OR pantozol OR pantor OR pantoloc OR astropan OR controloc OR pantecta OR inipomp OR somac OR pantodac OR zurcal OR zentro OR picoprazole OR “potassium competitive acid blocker” OR pumaprazole OR rabeprazole OR aciphex OR dexrabeprazole OR pariet OR zechin OR rabecid OR “nzole-d” OR rabeloc OR revaprazan OR saviprazole OR soraprazan OR tegoprazan OR timoprazole OR vonoprazan) OR **AB** (“proton pump inhibitor*” OR abeprazan OR benatoprazole OR tenatoprazole OR “STU-Na” OR esomeprazole OR esotrex OR alenia OR escz OR esofag OR nexiam OR ilaprazole OR lansoprazole OR lanzoprazole OR agopton OR bamalite OR inhibitol OR levant OR lupizole OR lanzor OR monolitum OR ogast OR ogastro OR opiren OR prevacid OR prezal OR “pro ulco” OR promeco OR takepron OR ulpax OR zoton OR dexlansoprazole OR kapidex OR dexilant OR levolansoprazole OR leminoprazole OR linaprazan OR nepaprazole OR omeprazole OR losec OR nexium OR prilosec OR rapinex OR zegerid OR

	ocid OR lomac OR omepral OR omez OR pantoprazole OR protium OR protonix OR pantotab OR pantopan OR pantozol OR pantor OR pantoloc OR astropan OR controloc OR pantecta OR inipomp OR somac OR pantodac OR zurcal OR zentro OR picoprazole OR “potassium competitive acid blocker” OR pumaprazole OR rabeprazole OR aciphex OR dexrabeprazole OR pariet OR zechin OR rabecid OR “nzole-d” OR rabeloc OR revaprazan OR saviprazole OR soraprazan OR tegoprazan OR timoprazole OR vonoprazan)
14	**TI** (proton N2 pump N2 inhibitor*) OR **AB** (proton N2 pump N2 inhibitor*)
15	**TI** ((antiulcer OR gastrointestinal OR “gastro intestinal” OR antireflux OR gastroesophageal OR “gastro esophageal” OR gastroprotect* OR “gastro protect*”) N2 (agent* OR drug OR drugs OR medication* OR medicine*)) OR **AB** ((antiulcer OR gastrointestinal OR “gastro intestinal” OR antireflux OR gastroesophageal OR “gastro esophageal” OR gastroprotect* OR “gastro protect*”) N2 (agent* OR drug OR drugs OR medication* OR medicine*))
16	**TI** (anti N2 (ulcer OR reflux) N2 (agent* OR drug OR drugs OR medication* OR medicine*)) OR **AB** (anti N2 (ulcer OR reflux) N2 (agent* OR drug OR drugs OR medication* OR medicine*))
17	**TI** ((peptic OR stress) N2 ulcer N2 (prophylaxis OR prophylactic)) OR **AB** ((peptic OR stress) N2 ulcer N2 (prophylaxis OR prophylactic))
18	**TI** ((gastrointestinal OR “gastro intestinal”) N2 (prophylaxis OR prophylactic)) OR **AB** ((gastrointestinal OR “gastro intestinal”) N2 (prophylaxis OR prophylactic))
19	**TI** ((gastric OR acid) N2 (suppressive OR suppression OR suppressant* OR suppressing OR suppressor*)) OR **AB** ((gastric OR acid) N2 (suppressive OR suppression OR suppressant* OR suppressing OR suppressor*))
20	**TI** antacid* OR **AB** antacid*
21	MH Proton Pump Inhibitors+ OR MH Esomeprazole OR MH Lansoprazole+ OR MH Dexlansoprazole OR MH Omeprazole+ OR MH Pantoprazole Sodium

	OR MH Rabeprazole Sodium OR MH Antiulcer Agents OR MH Antacids OR MH Gastrointestinal Agents
22	OR/13-21
23	12 AND 22
24	**TI** ((“proton pump inhibitor*” OR ppi OR ppis OR “ppi s” OR abeprazan OR benatoprazole OR tenatoprazole OR “stu-na” OR esomeprazole OR esotrex OR alenia OR escz OR esofag OR nexiam OR ilaprazole OR lansoprazole OR lanzoprazole OR agopton OR bamalite OR inhibitol OR levant OR lupizole OR lanzor OR monolitum OR ogast OR ogastro OR opiren OR prevacid OR prezal OR “pro ulco” OR promeco OR takepron OR ulpax OR zoton OR dexlansoprazole OR kapidex OR dexilant OR levolansoprazole OR leminoprazole OR linaprazan OR nepaprazole OR omeprazole OR losec OR nexium OR prilosec OR rapinex OR zegerid OR ocid OR lomac OR omepral OR omez OR pantoprazole OR protium OR protonix OR pantotab OR pantopan OR pantozol OR pantor OR pantoloc OR astropan OR controloc OR pantecta OR inipomp OR somac OR pantodac OR zurcal OR zentro OR picoprazole OR “potassium competitive acid blocker” OR pumaprazole OR rabeprazole OR aciphex OR dexrabeprazole OR pariet OR zechin OR rabecid OR “nzole-d” OR rabeloc OR revaprazan OR saviprazole OR soraprazan OR tegoprazan OR timoprazole OR vonoprazan OR “antiulcer agent*” OR “antiulcer drug*” OR “antiulcer medication*” OR “antiulcer* medicine*” OR “antiulcer treatment*” OR “antiulcer* therap*” OR “anti ulcer* agent*” OR “anti ulcer* drug*” OR “anti ulcer* medication*” OR “anti ulcer* medicine*” OR “anti ulcer* treatment*” OR “anti ulcer* therap*” OR “gastrointestinal agent*” OR “gastrointestinal drug*” OR “gastrointestinal medication*” OR “gastrointestinal medicine*” OR “gastro intestinal agent*” OR “gastro intestinal drug*” OR “gastro intestinal medication*” OR “gastro intestinal medicine*” OR “antireflux agent*” OR “antireflux drug*” OR “antireflux medication*” OR “antireflux medicine*” OR “antireflux treatment*” OR “antireflux therap*” OR “anti reflux agent*” OR “anti reflux drug*” OR “anti reflux medication*” OR “anti reflux medicine*” OR “anti reflux treatment*” OR “anti reflux therap*” OR “gastroesophageal agent*” OR “gastroesophageal drug*” OR “gastroesophageal medication*” OR “gastroesophageal medicine*” OR “gastroesophageal treatment*” OR “gastroesophageal therap*” OR “gastro esophageal agent*” OR “gastro esophageal drug*” OR “gastro esophageal medication*” OR “gastro

	esophageal medicine*” OR “gastro esophageal treatment*” OR “gastro esophageal therap*” OR “gastroprotect* agent*” OR “gastroprotect* drug*” OR “gastroprotect* medication*” OR “gastroprotect* medicine*” OR “gastroprotect* treatment*” OR “gastroprotect* therap*” OR “gastro protect* agent*” OR “gastro protect* drug*” OR “gastro protect* medication*” OR “gastro protect* medicine*” OR “gastro protect* treatment*” OR “gastro protect* therap*” OR “gastrointestinal prophylaxis” OR “gastro intestinal prophylaxis” OR “peptic ulcer prophylaxis” OR “stress ulcer prophylaxis” OR “stress ulcer prophylactic” OR “acid suppressive” OR “acid suppression” OR “acid suppressant*” OR “acid suppressing” OR “acid suppressor*” OR antacid*) N3 (appropriate* OR “no indication*” OR utilization* OR utilisation* OR justif* OR error* OR inadequate* OR excess* OR suboptimal* OR inadvertent* OR rational* OR harmful OR “long term” OR longterm OR risk* OR adverse OR optimal OR reasonable OR reasonably OR cost OR costs OR costly OR costing)) OR **AB** ((“proton pump inhibitor*” OR ppi OR ppis OR “ppi s” OR abeprazan OR benatoprazole OR tenatoprazole OR “stu-na” OR esomeprazole OR esotrex OR alenia OR escz OR esofag OR nexiam OR ilaprazole OR lansoprazole OR lanzoprazole OR agopton OR bamalite OR inhibitol OR levant OR lupizole OR lanzor OR monolitum OR ogast OR ogastro OR opiren OR prevacid OR prezal OR “pro ulco” OR promeco OR takepron OR ulpax OR zoton OR dexlansoprazole OR kapidex OR dexilant OR levolansoprazole OR leminoprazole OR linaprazan OR nepaprazole OR omeprazole OR losec OR nexium OR prilosec OR rapinex OR zegerid OR ocid OR lomac OR omepral OR omez OR pantoprazole OR protium OR protonix OR pantotab OR pantopan OR pantozol OR pantor OR pantoloc OR astropan OR controloc OR pantecta OR inipomp OR somac OR pantodac OR zurcal OR zentro OR picoprazole OR “potassium competitive acid blocker” OR pumaprazole OR rabeprazole OR aciphex OR dexrabeprazole OR pariet OR zechin OR rabecid OR “nzole-d” OR rabeloc OR revaprazan OR saviprazole OR soraprazan OR tegoprazan OR timoprazole OR vonoprazan OR “antiulcer agent*” OR “antiulcer drug*” OR “antiulcer medication*” OR “antiulcer* medicine*” OR “antiulcer treatment*” OR “antiulcer* therap*” OR “anti ulcer* agent*” OR “anti ulcer* drug*” OR “anti ulcer* medication*” OR “anti ulcer* medicine*” OR “anti ulcer* treatment*” OR “anti ulcer* therap*” OR “gastrointestinal agent*” OR “gastrointestinal drug*” OR “gastrointestinal medication*” OR “gastrointestinal medicine*” OR “gastro intestinal

	agent*" OR "gastro intestinal drug*" OR "gastro intestinal medication*" OR "gastro intestinal medicine*" OR "antireflux agent*" OR "antireflux drug*" OR "antireflux medication*" OR "antireflux medicine*" OR "antireflux treatment*" OR "antireflux therap*" OR "anti reflux agent*" OR "anti reflux drug*" OR "anti reflux medication*" OR "anti reflux medicine*" OR "anti reflux treatment*" OR "anti reflux therap*" OR "gastroesophageal agent*" OR "gastroesophageal drug*" OR "gastroesophageal medication*" OR "gastroesophageal medicine*" OR "gastroesophageal treatment*" OR "gastroesophageal therap*" OR "gastro esophageal agent*" OR "gastro esophageal drug*" OR "gastro esophageal medication*" OR "gastro esophageal medicine*" OR "gastro esophageal treatment*" OR "gastro esophageal therap*" OR "gastroprotect* agent*" OR "gastroprotect* drug*" OR "gastroprotect* medication*" OR "gastroprotect* medicine*" OR "gastroprotect* treatment*" OR "gastroprotect* therap*" OR "gastro protect* agent*" OR "gastro protect* drug*" OR "gastro protect* medication*" OR "gastro protect* medicine*" OR "gastro protect* treatment*" OR "gastro protect* therap*" OR "gastrointestinal prophylaxis" OR "gastro intestinal prophylaxis" OR "peptic ulcer prophylaxis" OR "stress ulcer prophylaxis" OR "stress ulcer prophylactic" OR "acid suppressive" OR "acid suppression" OR "acid suppressant*" OR "acid suppressing" OR "acid suppressor*" OR antacid*) N3 (appropriate* OR "no indication*" OR utilization* OR utilisation* OR justif* OR error* OR inadequate* OR excess* OR suboptimal* OR inadvertent* OR rational* OR harmful OR "long term" OR longterm OR risk* OR adverse OR optimal OR reasonable OR reasonably OR cost OR costs OR costly OR costing))
25	23 OR 24
26	**TI** (deprescri* OR "de prescri*" OR unprescri* OR "un prescri*" OR discontinu* OR reduction* OR "step* down" OR stepwise OR "step wise" OR "step off" OR compliance OR adherence OR audit OR audits OR taper* OR benchmark* OR "bench mark" OR TQM OR "academic detailing") OR **AB** (deprescri* OR "de prescri*" OR unprescri* OR "un prescri*" OR discontinu* OR reduction* OR "step* down" OR stepwise OR "step wise" OR "step off" OR compliance OR adherence OR audit OR audits OR taper* OR benchmark* OR "bench mark" OR TQM OR "academic detailing")
27	MH quality improvement OR MH quality assurance

28	**TI** (Quality n1 (control OR improvement* OR assurance OR assess* OR review* OR prescri* OR guideline* OR protocol* OR standard* OR policy OR policies OR regulation* OR management OR intervention OR strategy)) OR **AB** (Quality n1 (control OR improvement* OR assurance OR assess* OR review* OR prescri* OR guideline* OR protocol* OR standard* OR policy OR policies OR regulation* OR management OR intervention OR strategy))
29	MH benchmarking OR MH audit OR MH patient compliance OR MH medication compliance OR MH guideline adherence OR MH Practice guidelines OR MH substance withdrawal, controlled
30	**TI** ((Protocol* OR Guideline* OR policy OR policies OR regulation*) n3 (compliance OR adhere* OR implement* OR evaluat* OR comply OR compliant OR concord* OR distribut* OR disseminat* OR “best practice*” OR effective* OR “evidence base*” OR EBM OR introduce* OR issu* OR impact* OR adhering OR complying OR assess* OR review*)) OR **AB** ((Protocol* OR Guideline* OR policy OR policies OR regulation*) n3 (compliance OR adhere* OR implement* OR evaluat* OR comply OR compliant OR concord* OR distribut* OR disseminat* OR “best practice*” OR effective* OR “evidence base*” OR EBM OR introduce* OR issu* OR impact* OR adhering OR complying OR assess* OR review*))
31	**TI** (pharmacist* OR pharmd OR pharmacy OR pharmacies OR pharmacotherapeutic*) OR **AB** (pharmacist* OR pharmd OR pharmacy OR pharmacies OR pharmacotherapeutic*)
32	**TI** (pharmaceutical n2 (care OR service* OR consult* OR advice OR advise* OR advising OR intervention)) OR **AB** (pharmaceutical n2 (care OR service* OR consult* OR advice OR advise* OR advising OR intervention))
33	MH pharmacists OR MH pharmacy technicians OR MH pharmacist attitudes OR MH pharmacy service OR MH “pharmacy and pharmacology” OR MH Pharmacy, Retail
34	**TI** (“Screening test*” OR “screening tool*” OR “systematic tool*” OR Stopp OR “start criteria” OR “beers” OR “beer’s” OR “Pim Check” OR priscus OR “medication appropriateness index” OR FORTA OR mcleod* OR zhan OR

	ACOVE OR “pim list*” OR “pim criteria”OR mai) OR **AB** (“Screening test*” OR “screening tool*” OR “systematic tool*” OR Stopp OR “start criteria” OR “beers” OR “beer’s” OR “Pim Check” OR priscus OR “medication appropriateness index” OR FORTA OR mcleod* OR zhan OR ACOVE OR “pim list*” OR “pim criteria”OR mai)
35	**TI** ((record* OR medicine* OR chart* OR drug OR drugs OR dose* OR dosage* OR utilisation OR utilization OR usage OR medication* OR discharge OR prescri* OR appropriateness) N3 (monitor* OR management OR reconcil* OR review* OR evaluat* OR strategies OR strategy OR “evidence base*” OR EBM OR assess*)) OR **AB** ((record* OR medicine* OR chart* OR drug OR drugs OR dose* OR dosage* OR utilisation OR utilization OR usage OR medication* OR discharge OR prescri* OR appropriateness) N3 (monitor* OR management OR reconcil* OR review* OR evaluat* OR strategies OR strategy OR “evidence base*” OR EBM OR assess*))
36	MH record review OR MH medication reconciliation
37	MH cost control OR MH cost savings OR MH cost benefit analysis
38	**TI** ((cost OR costs) n2 (control OR savings OR analysis OR effective*)) OR **AB** ((cost OR costs) n2 (control OR savings OR analysis OR effective*))
39	**TI** ((reduce* OR reducing OR decrease* OR decreasing OR modif* OR alter* OR adapt* OR change* OR changing OR curtail* OR revise* OR revising OR revision OR adjust* OR deescalate* OR “de escalate*” OR lower* OR fewer OR minimize* OR minimise* OR minimizing OR minimising OR eliminating OR eliminate* OR elimination OR cessation OR cease* OR ceasing OR withheld OR withhold* OR withdraw* OR withdrew OR stop* OR review* OR curb* OR evaluat* OR assess* OR monitor*) n3 (intermittent OR usage OR use OR dosage* OR dose* OR dosing OR medication* OR medicine* OR prescri* OR drug OR drugs OR regimen* OR cost OR costs OR error* OR rate* OR polypharm* OR “poly pharm*” OR polymedicat* OR “poly medicat*” OR “long term” OR longterm OR duration OR PIM OR PIMs OR “pim’s” OR PPI OR PPIS OR “ppi’s” OR sup OR ast OR asm OR asms OR “asm’s” OR inappropriate* OR nonappropriat* OR “non appropriat*” OR overprescri* OR “over prescri*” OR overmedicat* OR “over medicat*” OR

	overus* OR “over use” OR mistreat* OR overtreat* OR overutili* OR “over utili*” OR improper* OR incorrect* OR unwarranted OR unjustified OR erroneous* OR misus* OR “un necessar*” OR unnecessar* OR utilization OR utilisation OR “practice pattern*”)) OR **AB** ((reduce* OR reducing OR decrease* OR decreasing OR modif* OR alter* OR adapt* OR change* OR changing OR curtail* OR revise* OR revising OR revision OR adjust* OR deescalate* OR “de escalate*” OR lower* OR fewer OR minimize* OR minimise* OR minimizing OR minimising OR eliminating OR eliminate* OR elimination OR cessation OR cease* OR ceasing OR withheld OR withhold* OR withdraw* OR withdrew OR stop* OR review* OR curb* OR evaluat* OR assess* OR monitor*) n3 (intermittent OR usage OR use OR dosage* OR dose* OR dosing OR medication* OR medicine* OR prescri* OR drug OR drugs OR regimen* OR cost OR costs OR error* OR rate* OR polypharm* OR “poly pharm*” OR polymedicat* OR “poly medicat*” OR “long term” OR longterm OR duration OR PIM OR PIMs OR “pim’s” OR PPI OR PPIS OR “ppi’s” OR sup OR ast OR asm OR asms OR “asm’s” OR inappropriate* OR nonappropriat* OR “non appropriat*” OR overprescri* OR “over prescri*” OR overmedicat* OR “over medicat*” OR overus* OR “over use” OR mistreat* OR overtreat* OR overutili* OR “over utili*” OR improper* OR incorrect* OR unwarranted OR unjustified OR erroneous* OR misus* OR “un necessar*” OR unnecessar* OR utilization OR utilisation OR “practice pattern*”))
40	MH drug monitoring
41	**TI** ((“proton pump inhibitor*” OR ppi OR ppis OR “ppi’s” OR abeprazan OR benatoprazole OR tenatoprazole OR “stu-na” OR esomeprazole OR esotrex OR alenia OR escz OR esofag OR nexiam OR ilaprazole OR lansoprazole OR lanzoprazole OR agopton OR bamalite OR inhibitol OR levant OR lupizole OR lanzor OR monolitum OR ogast OR ogastro OR opiren OR prevacid OR prezal OR “pro ulco” OR promeco OR takepron OR ulpax OR zoton OR dexlansoprazole OR kapidex OR dexilant OR levolansoprazole OR leminoprazole OR linaprazan OR nepaprazole OR omeprazole OR losec OR nexium OR prilosec OR rapinex OR zegerid OR ocid OR lomac OR omepral OR omez OR pantoprazole OR protium OR protonix OR pantotab OR pantopan OR pantozol OR pantor OR pantoloc OR astropan OR controloc OR pantecta OR inipomp OR somac OR pantodac OR zurcal OR zentro OR

	picoprazole OR “potassium competitive acid blocker” OR pumaprazole OR rabeprazole OR aciphex OR dexrabeprazole OR pariet OR zechin OR rabecid OR “nzole-d” OR rabeloc OR revaprazan OR saviprazole OR soraprazan OR tegoprazan OR timoprazole OR vonoprazan OR “antiulcer agent*” OR “antiulcer drug*” OR “antiulcer medication*” OR “antiulcer* medicine*” OR “antiulcer treatment*” OR “antiulcer* therap*” OR “anti ulcer* agent*” OR “anti ulcer* drug*” OR “anti ulcer* medication*” OR “anti ulcer* medicine*” OR “anti ulcer* treatment*” OR “anti ulcer* therap*” OR “gastrointestinal agent*” OR “gastrointestinal drug*” OR “gastrointestinal medication*” OR “gastrointestinal medicine*” OR “gastro intestinal agent*” OR “gastro intestinal drug*” OR “gastro intestinal medication*” OR “gastro intestinal medicine*” OR “antireflux agent*” OR “antireflux drug*” OR “antireflux medication*” OR “antireflux medicine*” OR “antireflux treatment*” OR “antireflux therap*” OR “anti reflux agent*” OR “anti reflux drug*” OR “anti reflux medication*” OR “anti reflux medicine*” OR “anti reflux treatment*” OR “anti reflux therap*” OR “gastroesophageal agent*” OR “gastroesophageal drug*” OR “gastroesophageal medication*” OR “gastroesophageal medicine*” OR “gastroesophageal treatment*” OR “gastroesophageal therap*” OR “gastro esophageal agent*” OR “gastro esophageal drug*” OR “gastro esophageal medication*” OR “gastro esophageal medicine*” OR “gastro esophageal treatment*” OR “gastro esophageal therap*” OR “gastroprotect* agent*” OR “gastroprotect* drug*” OR “gastroprotect* medication*” OR “gastroprotect* medicine*” OR “gastroprotect* treatment*” OR “gastroprotect* therap*” OR “gastro protect* agent*” OR “gastro protect* drug*” OR “gastro protect* medication*” OR “gastro protect* medicine*” OR “gastro protect* treatment*” OR “gastro protect* therap*” OR “gastrointestinal prophylaxis” OR “gastro intestinal prophylaxis” OR “peptic ulcer prophylaxis” OR “stress ulcer prophylaxis” OR “stress ulcer prophylactic” OR “acid suppressive” OR “acid suppression” OR “acid suppressant*” OR “acid suppressing” OR “acid suppressor*” OR antacid*) N3 (reduce* OR reducing OR decrease* OR decreasing OR modif* OR alter* OR change* OR changing OR curtail* OR revise* OR revising OR revision OR adjust* OR deescalate* OR “de escalate*” OR lower* OR fewer OR minimize* OR minimise* OR minimizing OR minimising OR eliminate* OR elimination OR cessation OR cease* OR ceasing OR withheld OR withhold* OR withdraw* OR withdrew OR stop* OR monitor* OR review* OR curb* OR evaluat* OR

	assess*)) OR **AB** ((“proton pump inhibitor*” OR ppi OR ppis OR “ppi’s” OR abeprazan OR benatoprazole OR tenatoprazole OR “stu-na” OR esomeprazole OR esotrex OR alenia OR escz OR esofag OR nexiam OR ilaprazole OR lansoprazole OR lanzoprazole OR agopton OR bamalite OR inhibitol OR levant OR lupizole OR lanzor OR monolitum OR ogast OR ogastro OR opiren OR prevacid OR prezal OR “pro ulco” OR promeco OR takepron OR ulpax OR zoton OR dexlansoprazole OR kapidex OR dexilant OR levolansoprazole OR leminoprazole OR linaprazan OR nepaprazole OR omeprazole OR losec OR nexium OR prilosec OR rapinex OR zegerid OR ocid OR lomac OR omepral OR omez OR pantoprazole OR protium OR protonix OR pantotab OR pantopan OR pantozol OR pantor OR pantoloc OR astropan OR controloc OR pantecta OR inipomp OR somac OR pantodac OR zurcal OR zentro OR picoprazole OR “potassium competitive acid blocker” OR pumaprazole OR rabeprazole OR aciphex OR dexrabeprazole OR pariet OR zechin OR rabecid OR “nzole-d” OR rabeloc OR revaprazan OR saviprazole OR soraprazan OR tegoprazan OR timoprazole OR vonoprazan OR “antiulcer agent*” OR “antiulcer drug*” OR “antiulcer medication*” OR “antiulcer* medicine*” OR “antiulcer treatment*” OR “antiulcer* therap*” OR “anti ulcer* agent*” OR “anti ulcer* drug*” OR “anti ulcer* medication*” OR “anti ulcer* medicine*” OR “anti ulcer* treatment*” OR “anti ulcer* therap*” OR “gastrointestinal agent*” OR “gastrointestinal drug*” OR “gastrointestinal medication*” OR “gastrointestinal medicine*” OR “gastro intestinal agent*” OR “gastro intestinal drug*” OR “gastro intestinal medication*” OR “gastro intestinal medicine*” OR “antireflux agent*” OR “antireflux drug*” OR “antireflux medication*” OR “antireflux medicine*” OR “antireflux treatment*” OR “antireflux therap*” OR “anti reflux agent*” OR “anti reflux drug*” OR “anti reflux medication*” OR “anti reflux medicine*” OR “anti reflux treatment*” OR “anti reflux therap*” OR “gastroesophageal agent*” OR “gastroesophageal drug*” OR “gastroesophageal medication*” OR “gastroesophageal medicine*” OR “gastroesophageal treatment*” OR “gastroesophageal therap*” OR “gastro esophageal agent*” OR “gastro esophageal drug*” OR “gastro esophageal medication*” OR “gastro esophageal medicine*” OR “gastro esophageal treatment*” OR “gastro esophageal therap*” OR “gastroprotect* agent*” OR “gastroprotect* drug*” OR “gastroprotect* medication*” OR “gastroprotect* medicine*” OR “gastroprotect* treatment*” OR “gastroprotect* therap*” OR “gastro protect* agent*” OR “gastro protect*

	drug*" OR "gastro protect* medication*" OR "gastro protect* medicine*" OR "gastro protect* treatment*" OR "gastro protect* therap*" OR "gastrointestinal prophylaxis" OR "gastro intestinal prophylaxis" OR "peptic ulcer prophylaxis" OR "stress ulcer prophylaxis" OR "stress ulcer prophylactic" OR "acid suppressive" OR "acid suppression" OR "acid suppressant*" OR "acid suppressing" OR "acid suppressor*" OR antacid*) N3 (reduce* OR reducing OR decrease* OR decreasing OR modif* OR alter* OR change* OR changing OR curtail* OR revise* OR revising OR revision OR adjust* OR deescalate* OR "de escalate*" OR lower* OR fewer OR minimize* OR minimise* OR minimizing OR minimising OR eliminate* OR elimination OR cessation OR cease* OR ceasing OR withheld OR withhold* OR withdraw* OR withdrew OR stop* OR monitor* OR review* OR curb* OR evaluat* OR assess*))
42	OR/26-41
43	25 AND 42

	EMBASE
1	(inappropriate* OR nonappropriate* OR “non appropriate*” OR nonindicat* OR “non indicat*” OR (“with out” NEAR/4 indication*) OR (without NEAR/4 indication*) OR polypharm* OR “poly pharm*” OR polymedicat* OR “poly medicat*” OR overprescri* OR “over prescri*” OR overmedicat* OR “over medicat*” OR overus* OR “over use” OR mistreat* OR overtreat* OR overutili* OR “over utili*” OR improper* OR incorrect* OR unwarranted OR unjustified OR erroneous* OR misus* OR “un necessar*” OR unnecessar* OR “sub optimal” OR “repeat* prescri*” OR “repeat* medicine*” OR “repeat* medication*” OR irrational* OR expensive OR expense* OR expenditure* OR “drug related problem*” OR “medication related problem*” OR indiscriminate* OR discrepanc* OR nonguideline* OR “non guideline*” OR questionable OR omission* OR unlicense* OR unapprov* OR “un approv*” OR “dispens* error*” OR wrong* OR “near miss” OR “utilization* pattern*” OR “utilisation* pattern*” OR “practice pattern*” OR “multiple medication*” OR “non beneficial” OR nonbeneficial OR unsafe*):ti,ab,kw
2	((improve* OR improving OR optimize* OR optimizing OR optimise* OR optimising) NEAR/3 (safety OR usage OR use OR prescri*)):ti,ab,kw
3	((cost OR costs OR costly OR costing) NEAR/3 (high* OR increase* OR grow* OR financial OR economic OR drug OR drugs OR prescri* OR medicine* OR medication* OR dose* OR dosing OR dosage* OR therap* OR treat* OR use OR usage)):ti,ab,kw
4	(prescri* NEAR/3 (pattern* OR habit* OR practice* OR regimen* OR trend* OR behavior* OR behaviour* OR cascade* OR record* OR indication*)):ti,ab,kw
5	(reconcil* NEAR/3 error*):ti,ab,kw
6	(safe* NEAR/3 prescri*):ti,ab,kw
7	((usage OR use OR dosage* OR dose* OR dosing OR medication* OR medicine* OR prescri* OR drug OR drugs OR therap* OR treat* OR indication* OR cost OR costs OR costly OR costing OR dispens*) NEAR/3

	(appropriate* OR utilization* OR utilisation* OR justif* OR error* OR inadequate* OR excess* OR suboptimal* OR inadvertent* OR rational* OR harmful OR “long term” OR longterm OR optimal OR reasonable OR reasonably)):ti,ab,kw
8	(risk* NEAR/3 (usage OR use OR medication* OR medicine* OR prescri*)):ti,ab,kw
9	((nonadhere* OR “non adhere*” OR noncompliance OR noncompliant OR “non compliance” OR “non compliant”) NEAR/3 (guideline* OR indication* OR prescri* OR protocol* OR policy OR policies OR regulation*)):ti,ab,kw
10	((valid* OR accura* OR approve*) NEAR/3 (prescri* OR indication*)):ti,ab,kw
11	'inappropriate prescribing'/exp OR 'potentially inappropriate medication'/de OR 'polypharmacy'/exp OR 'drug misuse'/exp OR 'prescription drug misuse'/exp OR 'medication overuse'/de OR 'medication error'/exp OR 'medical error'/de OR 'long term care'/de OR 'drug utilization'/exp OR 'prevention'/de
12	OR/1-11
13	(“proton pump inhibitor*” OR abeprazan OR benatoprazole OR tenatoprazole OR “TU 199” OR “CAS 113712-98-4” OR “STU-Na” OR esomeprazole OR esotrex OR alenia OR escz OR esofag OR nexiam OR ilaprazole OR lansoprazole OR lanzoprazole OR agopton OR bamalite OR inhibitol OR levant OR lupizole OR lanzor OR monolitum OR ogast OR ogastro OR opiren OR prevacid OR prezal OR “pro ulco” OR promeco OR takepron OR ulpax OR zoton OR dexlansoprazole OR kapidex OR dexilant OR levolansoprazole OR leminoprazole OR linaprazan OR nepaprazole OR omeprazole OR losec OR nexium OR prilosec OR rapinex OR zegerid OR ocid OR lomac OR omepral OR omez OR pantoprazole OR protium OR protonix OR pantotab OR pantopan OR pantozol OR pantor OR pantoloc OR astropan OR controloc OR pantecta OR inipomp OR somac OR pantodac OR zurcal OR zentro OR picoprazole OR “potassium competitive acid blocker” OR pumaprazole OR rabeprazole OR aciphex OR dexrabeprazole OR pariet OR zechin OR rabecid OR “nzole-d” OR rabeloc

	OR revaprazan OR saviprazole OR soraprazan OR tegoprazan OR timoprazole OR vonoprazan):ti,ab,kw
14	(proton NEAR/2 pump NEAR/2 inhibitor*):ti,ab,kw
15	((antiulcer OR gastrointestinal OR “gastro intestinal” OR antireflux OR gastroesophageal OR “gastro esophageal” OR gastroprotect* OR “gastro protect*”) NEAR/2 (agent* OR drug OR drugs OR medication* OR medicine*)):ti,ab,kw
16	(anti NEAR/2 (ulcer OR reflux) NEAR/2 (agent* OR drug OR drugs OR medication* OR medicine*)):ti,ab,kw
17	((peptic OR stress) NEAR/2 ulcer NEAR/2 (prophylaxis OR prophylactic)):ti,ab,kw
18	((gastrointestinal OR “gastro intestinal”) NEAR/2 (prophylaxis OR prophylactic)):ti,ab,kw
19	((gastric OR acid) NEAR/2 (suppressive OR suppression OR suppressant* OR suppressing OR suppressor*)):ti,ab,kw
20	antacid*:ti,ab,kw
21	'proton pump inhibitor'/exp OR 'benatoprazole'/de OR 'esomeprazole'/de OR 'lansoprazole'/exp OR 'dexlansoprazole'/de OR 'omeprazole'/de OR 'pantoprazole'/de OR 'rabeprazole'/de OR 'antiulcer agent'/de OR 'gastrointestinal agent'/de OR 'antacid agent'/de OR “stress ulcer prophylaxis”/de OR “acid suppression therapy”/de
22	OR/13-21
23	12 AND 22
24	((“proton pump inhibitor*” OR ppi OR ppis OR “ppi s” OR abeprazan OR benatoprazole OR tenatoprazole OR “tu 199” OR “cas 113712-98-4” OR “stu-na” OR esomeprazole OR esotrex OR alenia OR escz OR esofag OR nexiam OR ilaprazole OR lansoprazole OR lanzoprazole OR agopton OR bamalite OR inhibitol OR levant OR lupizole OR lanzor OR monolitum OR ogast OR ogastro OR opiren OR prevacid OR prezal OR “pro ulco” OR promeco OR takepron OR ulpax OR zoton OR dexlansoprazole OR kapidex

	OR dexilant OR levolansoprazole OR leminoprazole OR linaprazan OR nepaprazole OR omeprazole OR losec OR nexium OR prilosec OR rapinex OR zegerid OR ocid OR lomac OR omepral OR omez OR pantoprazole OR protium OR protonix OR pantotab OR pantopan OR pantozol OR pantor OR pantoloc OR astropan OR controloc OR pantecta OR inipomp OR somac OR pantodac OR zurcal OR zentro OR picoprazole OR “potassium competitive acid blocker” OR pumaprazole OR rabeprazole OR aciphex OR dexrabeprazole OR pariet OR zechin OR rabecid OR “nzole-d” OR rabeloc OR revaprazan OR saviprazole OR soraprazan OR tegoprazan OR timoprazole OR vonoprazan OR “antiulcer agent*” OR “antiulcer drug*” OR “antiulcer medication*” OR “antiulcer* medicine*” OR “antiulcer treatment*” OR “antiulcer* therap*” OR “anti ulcer* agent*” OR “anti ulcer* drug*” OR “anti ulcer* medication*” OR “anti ulcer* medicine*” OR “anti ulcer* treatment*” OR “anti ulcer* therap*” OR “gastrointestinal agent*” OR “gastrointestinal drug*” OR “gastrointestinal medication*” OR “gastrointestinal medicine*” OR “gastro intestinal agent*” OR “gastro intestinal drug*” OR “gastro intestinal medication*” OR “gastro intestinal medicine*” OR “antireflux agent*” OR “antireflux drug*” OR “antireflux medication*” OR “antireflux medicine*” OR “antireflux treatment*” OR “antireflux therap*” OR “anti reflux agent*” OR “anti reflux drug*” OR “anti reflux medication*” OR “anti reflux medicine*” OR “anti reflux treatment*” OR “anti reflux therap*” OR “gastroesophageal agent*” OR “gastroesophageal drug*” OR “gastroesophageal medication*” OR “gastroesophageal medicine*” OR “gastroesophageal treatment*” OR “gastroesophageal therap*” OR “gastro esophageal agent*” OR “gastro esophageal drug*” OR “gastro esophageal medication*” OR “gastro esophageal medicine*” OR “gastro esophageal treatment*” OR “gastro esophageal therap*” OR “gastroprotect* agent*” OR “gastroprotect* drug*” OR “gastroprotect* medication*” OR “gastroprotect* medicine*” OR “gastroprotect* treatment*” OR “gastroprotect* therap*” OR “gastro protect* agent*” OR “gastro protect* drug*” OR “gastro protect* medication*” OR “gastro protect* medicine*” OR “gastro protect* treatment*” OR “gastro protect* therap*” OR “gastrointestinal prophylaxis” OR “gastro intestinal prophylaxis” OR “peptic ulcer prophylaxis” OR “stress ulcer prophylaxis” OR “stress ulcer prophylactic” OR “acid suppressive” OR “acid suppression” OR “acid suppressant*” OR “acid suppressing” OR “acid suppressor*” OR antacid*) **NEAR/3**

	(appropriate* OR “no indication*” OR utilization* OR utilisation* OR justif* OR error* OR inadequate* OR excess* OR suboptimal* OR inadvertent* OR rational* OR harmful OR “long term” OR longterm OR risk* OR adverse OR optimal OR reasonable OR reasonably OR cost OR costs OR costly OR costing)):ti,ab,kw
25	23 OR 24
26	(deprescri* OR “de prescri*” OR unprescri* OR “un prescri*” OR discontinu* OR reduction* OR “step* down” OR stepwise OR “step wise” OR “step off” OR compliance OR adherence OR audit OR audits OR taper* OR benchmark* OR “bench mark*” OR “academic detailing” OR TQM):ti,ab,kw
27	“quality control”/de OR “total quality management”/exp
28	(Quality near/1 (control OR improvement OR assurance OR assess* OR review* OR prescri* OR guideline* OR protocol* OR standard* OR policy OR policies OR regulation* OR management OR intervention OR strategy)):ti,ab,kw
29	benchmarking/exp OR “risk reduction”/exp OR “drug dose reduction”/exp OR “clinical audit”/exp OR deprescription/exp OR deprescribing/exp OR “patient compliance”/de OR “protocol compliance”/exp OR “medication compliance”/exp OR adherence/exp OR Standards/exp OR “Practice guideline”/exp OR “drug withdrawal”/exp OR “treatment withdrawal”/exp
30	((Protocol* OR Guideline* OR policy OR policies OR regulation*) near/3 (compliance OR adhere* OR implement* OR evaluat* OR comply OR compliant OR complying OR adhering OR concord* OR distribut* OR disseminat* OR “best practice*” OR effective* OR “evidence base*” OR EBM OR introduce* OR issu* OR impact* OR review* OR assess*)):ti,ab,kw
31	Pharmacist/exp OR “pharmacist attitude”/exp OR “pharmacy student”/exp OR pharmacy/exp OR “hospital pharmacy”/exp OR “pharmacy and therapeutics committee”/exp OR “clinical pharmacy”/exp OR “pharmaceutical care”/exp

32	(Pharmacy OR Pharmacies OR pharmacotherapeutic* OR pharmacist* OR pharmd):ti,ab,kw
33	(pharmaceutical Near/2 (care OR service* OR consult* OR advice OR advise* OR advising OR intervention)):ti,ab,kw
34	("Screening test*" OR "screening tool*" OR "systematic tool*" OR Stopp OR "start criteria" OR beers OR "beer s" OR "Pim Check" OR priscus OR "medication appropriateness index" OR FORTA OR mcleod* OR zhan OR ACOVE OR "pim list*" OR "pim criteria" OR mai):ti,ab,kw
35	((record* OR medicine* OR chart* OR drug OR drugs OR dose* OR dosage* OR utilisation OR utilization OR usage OR medication* OR discharge OR prescri* OR appropriateness) NEAR/3 (assess* OR monitor* OR management OR reconcil* OR review* OR evaluat* OR strategies OR strategy OR "evidence base*" OR EBM)):ti,ab,kw
36	"Drug utilization review"/exp OR "Medication therapy management"/exp OR "Medical record review"/exp OR "academic detailing"/de
37	"cost control"/exp OR "cost savings"/exp OR "cost benefit analysis"/exp
38	((cost OR costs) near/2 (control OR savings OR analysis OR effective*)):ti,ab,kw
39	((reduce* OR reducing OR decrease* OR decreasing OR modif* OR alter* OR adapt* OR change* OR changing OR curtail* OR revise* OR revising OR revision OR adjust* OR deescalate* OR "de escalate*" OR lower* OR fewer OR minimize* OR minimise* OR minimizing OR minimising OR eliminating OR eliminate* OR elimination OR cessation OR cease* OR ceasing OR withheld OR withhold* OR withdraw* OR withdrew OR stop* OR review* OR curb* OR evaluat* OR assess* OR monitor*) near/3 (usage OR use OR dosage* OR dose* OR dosing OR medication* OR medicine* OR prescri* OR "on demand" OR intermittent OR drug OR drugs OR regimen* OR cost OR costs OR error* OR rate* OR polypharm* OR "poly pharm*" OR polymedicat* OR "poly medicat*" OR "long term" OR longterm OR duration OR PIM OR PIMs OR "pim s" OR PPI OR PPIS OR "ppi s" OR sup OR

	ast OR asm OR asms OR “asm s” OR inappropriate* OR nonappropriat* OR “non appropriat*” OR overprescri* OR “over prescri*” OR overmedicat* OR “over medicat*” OR overus* OR “over use” OR mistreat* OR overtreat* OR overutili* OR “over utili*” OR improper* OR incorrect* OR unwarranted OR unjustified OR erroneous* OR misus* OR “un necessar*” OR unnecessar* OR utilization OR utilisation OR “practice pattern*”)):ti,ab,kw
40	“Drug monitoring”/exp
41	(('proton pump inhibitor*' OR ppi OR ppis OR “ppi s” OR abeprazan OR benatoprazole OR tenatoprazole OR 'tu 199' OR 'cas 113712-98-4' OR 'stu-na' OR esomeprazole OR esotrex OR alenia OR escz OR esofag OR nexiam OR ilaprazole OR lansoprazole OR lanzoprazole OR agopton OR bamalite OR inhibitol OR levant OR lupizole OR lanzor OR monolitum OR ogast OR ogastro OR opiren OR prevacid OR prezal OR 'pro ulco' OR promeco OR takepron OR ulpax OR zoton OR dexlansoprazole OR kapidex OR dexilant OR levolansoprazole OR leminoprazole OR linaprazan OR nepaprazole OR omeprazole OR losec OR nexium OR prilosec OR rapinex OR zegerid OR ocid OR lomac OR omepral OR omez OR pantoprazole OR protium OR protonix OR pantotab OR pantopan OR pantozol OR pantor OR pantoloc OR astropan OR controloc OR pantecta OR inipomp OR somac OR pantodac OR zurcal OR zentro OR picoprazole OR 'potassium competitive acid blocker' OR pumaprazole OR rabeprazole OR aciphex OR dexrabeprazole OR pariet OR zechin OR rabecid OR 'nzole-d' OR rabeloc OR revaprazan OR saviprazole OR soraprazan OR tegoprazan OR timoprazole OR vonoprazan OR 'antiulcer agent*' OR 'antiulcer drug*' OR 'antiulcer medication*' OR 'antiulcer* medicine*' OR 'antiulcer treatment*' OR 'antiulcer* therap*' OR 'anti ulcer* agent*' OR 'anti ulcer* drug*' OR 'anti ulcer* medication*' OR 'anti ulcer* medicine*' OR 'anti ulcer* treatment*' OR 'anti ulcer* therap*' OR 'gastrointestinal agent*' OR 'gastrointestinal drug*' OR 'gastrointestinal medication*' OR 'gastrointestinal medicine*' OR 'gastro intestinal agent*' OR 'gastro intestinal drug*' OR 'gastro intestinal medication*' OR 'gastro intestinal medicine*' OR 'antireflux agent*' OR 'antireflux drug*' OR 'antireflux medication*' OR 'antireflux medicine*' OR 'antireflux treatment*' OR 'antireflux therap*' OR 'anti reflux agent*' OR 'anti reflux drug*' OR 'anti

	reflux medication*' OR 'anti reflux medicine*' OR 'anti reflux treatment*' OR 'anti reflux therap*' OR 'gastroesophageal agent*' OR 'gastroesophageal drug*' OR 'gastroesophageal medication*' OR 'gastroesophageal medicine*' OR 'gastroesophageal treatment*' OR 'gastroesophageal therap*' OR 'gastro esophageal agent*' OR 'gastro esophageal drug*' OR 'gastro esophageal medication*' OR 'gastro esophageal medicine*' OR 'gastro esophageal treatment*' OR 'gastro esophageal therap*' OR 'gastroprotect* agent*' OR 'gastroprotect* drug*' OR 'gastroprotect* medication*' OR 'gastroprotect* medicine*' OR 'gastroprotect* treatment*' OR 'gastroprotect* therap*' OR 'gastro protect* agent*' OR 'gastro protect* drug*' OR 'gastro protect* medication*' OR 'gastro protect* medicine*' OR 'gastro protect* treatment*' OR 'gastro protect* therap*' OR 'gastrointestinal prophylaxis' OR “gastro intestinal prophylaxis” OR 'peptic ulcer prophylaxis' OR 'stress ulcer prophylaxis' OR 'stress ulcer prophylactic' OR “acid suppressive” OR “acid suppression” OR “acid suppressant*” OR “acid suppressing” OR “acid suppressor*” OR antacid*) NEAR/3 (reduce* OR reducing OR decrease* OR decreasing OR modif* OR alter* OR change* OR changing OR curtail* OR revise* OR revising OR revision OR adjust* OR deescalate* OR “de escalate*” OR lower* OR fewer OR minimize* OR minimise* OR minimizing OR minimising OR eliminate* OR elimination OR eliminating OR cessation OR cease* OR ceasing OR withheld OR withhold* OR withdraw* OR withdrew OR stop* OR monitor* OR review* OR curb* OR evaluat* OR assess* OR manage*)):ti,ab,kw
42	OR/26-41
43	25 AND 42
44	Animal/exp OR “animal experiment”/exp OR “animal model”/exp OR invertebrate/exp OR “animal tissue”/exp OR “animal cell”/exp OR nonhuman/exp NOT (human/exp OR “normal human”/exp)
45	43 NOT 44

	ClinicalTrials.gov
1	(“proton pump inhibitors” OR PPIs OR esomeprazole OR lansoprazole OR dexlansoprazole OR omeprazole OR pantoprazole OR rabeprazole OR “anti ulcer agent” OR “acid suppressive therapy” OR “acid suppression therapy” OR “acid suppressing therapy” OR “stress ulcer prophylaxis”) AND (guidelines OR inappropriate OR pharmacists OR polypharmacy OR "medication errors" OR deprescribe OR deprescription OR deprescribing OR "academic detailing" OR reconciliation OR "practice patterns" OR "prescribing patterns" OR “appropriate prescribing” OR “appropriate indication” OR overuse OR "step down" OR intermittent OR "on demand" OR discontinue OR taper)

	Web of Science Core Collection: Science Citation Index Expanded, Social Sciences Citation Index, Conference Proceedings Citation Index-Science, Conference Proceedings Citation Index-Social Science & Humanities
1	TS=("inappropriate*" OR "nonappropriate*" OR "non appropriate*" OR "nonindicat*" OR "non indicat*" OR ("without NEAR/4 indication*") OR "polypharm*" OR "poly pharm*" OR "polymedicat*" OR "poly medicat*" OR "overprescri*" OR "over prescri*" OR "overmedicat*" OR "over medicat*" OR "overus*" OR "over use" OR "mistreat*" OR "overtreat*" OR "overutili*" OR "over utili*" OR "improper*" OR "incorrect*" OR "unwarranted" OR "unjustified" OR "erroneous*" OR "misus*" OR "un necessar*" OR "unnecessar*" OR "sub optimal" OR "repeat* prescri*" OR "repeat* medicine*" OR "repeat* medication*" OR "irrational*" OR "expensive" OR "expense*" OR "expenditure*" OR "drug related problem*" OR "medication related problem*" OR "indiscriminate*" OR "discrepanc*" OR "nonguideline*" OR "non guideline*" OR "questionable" OR "omission*" OR "unlicense*" OR "unapprov*" OR "un approv*" OR "dispens* error*" OR "wrong*" OR "near miss" OR "utilization* pattern*" OR "utilisation* pattern*" OR "practice pattern*" OR "multiple medication*" OR "non beneficial" OR "nonbeneficial" OR "unsafe*")
2	TS=(("improve*" OR "improving" OR "optimize*" OR "optimizing" OR "optimise*" OR "optimising") NEAR/3 ("safety" OR "usage" OR "use" OR "prescri*"))
3	TS=(("cost" OR "costs" OR "costly" OR "costing") NEAR/3 ("high*" OR "increase*" OR "grow*" OR "financial" OR "economic" OR "drug" OR "drugs" OR "prescri*" OR "medicine*" OR "medication*" OR "dose*" OR "dosing" OR "dosage*" OR "therap*" OR "treat*" OR "use" OR "usage"))
4	TS=("prescri*" NEAR/3 ("pattern*" OR "habit*" OR "practice*" OR "regimen*" OR "trend*" OR "behavior*" OR "behaviour*" OR "cascade*" OR "record*" OR "indication*"))
5	TS=("reconcil*" NEAR/3 "error*")
6	TS=("safe*" NEAR/3 "prescri*")

7	TS=((“usage” OR “use” OR “dosage*” OR “dose*” OR “dosing” OR “medication*” OR “medicine*” OR “prescri*” OR “drug” OR “drugs” OR “therap*” OR “treat*” OR “indication*” OR “cost” OR “costs” OR “costly” OR “costing” OR “dispens*”) NEAR/3 (“appropriate*” OR “utilization*” OR “utilisation*” OR “justif*” OR “error*” OR “inadequate*” OR “excess*” OR “suboptimal*” OR “inadvertent*” OR “rational*” OR “harmful” OR “long term” OR “longterm” OR “optimal” OR “reasonable” OR “reasonably”))
8	TS=(“risk*” NEAR/3 (“usage” OR “use” OR “medication*” OR “medicine*” OR “prescri*”))
9	TS=((“nonadhere*” OR “non adhere*” OR “noncompliance” OR “noncompliant” OR “non compliance” OR “non compliant”) NEAR/3 (“guideline*” OR “indication*” OR “prescri*” OR “protocol*” OR “policy” OR “policies” OR “regulation*”))
10	TS=((“valid*” OR “accura*” OR “approve*”) NEAR/3 (“prescri*” OR “indication*”))
11	OR/1-10
12	TS=(“proton pump inhibitor*” OR “abeprazan” OR “benatoprazole” OR “tenatoprazole” OR “TU 199” OR “CAS 113712-98-4” OR “STU-Na” OR “esomeprazole” OR “esotrex” OR “alenia” OR “escz” OR “esofag” OR “nexiam” OR “ilaprazole” OR “lansoprazole” OR “lansoprazole” OR “agopton” OR “bamalite” OR “inhibitol” OR “levant” OR “lupizole” OR “lanzor” OR “monolitum” OR “ogast” OR “ogastro” OR “opiren” OR “prevacid” OR “prezal” OR “pro ulco” OR “promeco” OR “takepron” OR “ulpax” OR “zoton” OR “dexlansoprazole” OR “kapidex” OR “dexilant” OR “levolansoprazole” OR “leminoprazole” OR “linaprazan” OR “nepaprazole” OR “omeprazole” OR “losec” OR “nexium” OR “Prilosec” OR “rapinex" OR “zegerid” OR “ocid” OR “lomac” OR “omepral” OR “omez” OR “pantoprazole” OR “protium” OR “protonix” OR “pantotab” OR “pantopan” OR “pantozol” OR “pantor” OR “pantoloc” OR “astropan” OR “controloc” OR “pantecta” OR “inipomp” OR “somac” OR “pantodac” OR “zurcal” OR “zentro” OR “picoprazole” OR “potassium competitive acid blocker” OR “pumaprazole” OR “rabeprazole” OR “aciphex” OR

	“dexrabeprazole” OR “pariet” OR “zechin” OR “rabecid” OR “nzole-d” OR “rabeloc” OR “revaprazan” OR “saviprazole” OR “soraprazan” OR “tegoprazan” OR “timoprazole” OR “vonoprazan”)
13	TS=(“proton” NEAR/2 “pump” NEAR/2 “inhibitor*”)
14	TS=((“antiulcer” OR “gastrointestinal” OR “gastro intestinal” OR “antireflux” OR “gastroesophageal” OR “gastro esophageal” OR “gastroprotect*” OR “gastro protect*”) NEAR/2 (“agent*” OR “drug” OR “drugs” OR “medication*” OR “medicine*”))
15	TS=(“anti” NEAR/2 (“ulcer” OR “reflux”) NEAR/2 (“agent*” OR “drug” OR “drugs” OR “medication*” OR “medicine*”))
16	TS=((“peptic” OR “stress”) NEAR/2 “ulcer” NEAR/2 (“prophylaxis” OR “prophylactic”))
17	TS=((“gastrointestinal” OR “gastro intestinal”) NEAR/2 (“prophylaxis” OR “prophylactic”))
18	TS=((“gastric” OR “acid”) NEAR/2 (“suppressive” OR “suppression” OR “suppressant*” OR “suppressing” OR “suppressor*”))
19	TS=“antacid*”
20	OR/12-19
21	11 AND 20
22	TS=((“proton pump inhibitor*” OR “ppi” OR “ppis” OR “ppi s” OR “abeprazan” OR “benatoprazole” OR “tenatoprazole” OR “tu 199” OR “cas 113712-98-4” OR “stu-na” OR “esomeprazole” OR “esotrex” OR “alenia” OR “escz” OR “esofag” OR “nexiam” OR “ilaprazole” OR “lansoprazole” OR “lansoprazole” OR “agopton” OR “bamalite” OR “Inhibitol” OR “levant” OR “lupizole” OR “lanzor” OR “monolitum” OR “ogast” OR “ogastro” OR “opiren” OR “prevacid” OR “prezal” OR “pro ulco” OR “promeco” OR “takepron” OR “ulpax” OR “zoton” OR “dexlansoprazole” OR “kapidex” OR “dexilant” OR “levolansoprazole” OR “leminoprazole” OR “linaprazan” OR “nepaprazole” OR “omeprazole” OR “losec” OR “Nexium” OR “Prilosec”

	OR “rapinex" OR “zegerid” OR “ocid” OR “lomac” OR “omepral” OR “omez” OR “pantoprazole” OR “protium” OR “protonix” OR “pantotab” OR “pantopan” OR “pantozol” OR “pantor” OR “pantoloc” OR “astropan” OR “controloc” OR “pantecta” OR “inipomp” OR “somac” OR “pantodac” OR “zurcal” OR “zentro” OR “picoprazole” OR “potassium competitive acid blocker” OR “pumaprazole” OR “rabeprazole” OR “aciphex” OR “dexrabeprazole” OR “pariet” OR “zechin” OR “rabecid” OR “nzole-d” OR “rabeloc” OR “revaprazan” OR “saviprazole” OR “soraprazan” OR “tegoprazan” OR “timoprazole” OR “vonoprazan” OR “antiulcer agent*” OR “antiulcer drug*” OR “antiulcer medication*” OR “antiulcer* medicine*” OR “antiulcer treatment*” OR “antiulcer* therap*” OR “anti ulcer* agent*” OR “anti ulcer* drug*” OR “anti ulcer* medication*” OR “anti ulcer* medicine*” OR “anti ulcer* treatment*” OR “anti ulcer* therap*” OR “gastrointestinal agent*” OR “gastrointestinal drug*” OR “gastrointestinal medication*” OR “gastrointestinal medicine*” OR “gastro intestinal agent*” OR “gastro intestinal drug*” OR “gastro intestinal medication*” OR “gastro intestinal medicine*” OR “antireflux agent*” OR “antireflux drug*” OR “antireflux medication*” OR “antireflux medicine*” OR “antireflux treatment*” OR “antireflux therap*” OR “anti reflux agent*” OR “anti reflux drug*” OR “anti reflux medication*” OR “anti reflux medicine*” OR “anti reflux treatment*” OR “anti reflux therap*” OR “gastroesophageal agent*” OR “gastroesophageal drug*” OR “gastroesophageal medication*” OR “gastroesophageal medicine*” OR “gastroesophageal treatment*” OR “gastroesophageal therap*” OR “gastro esophageal agent*” OR “gastro esophageal drug*” OR “gastro esophageal medication*” OR “gastro esophageal medicine*” OR “gastro esophageal treatment*” OR “gastro esophageal therap*” OR “gastroprotect* agent*” OR “gastroprotect* drug*” OR “gastroprotect* medication*” OR “gastroprotect* medicine*” OR “gastroprotect* treatment*” OR “gastroprotect* therap*” OR “gastro protect* agent*” OR “gastro protect* drug*” OR “gastro protect* medication*” OR “gastro protect* medicine*” OR “gastro protect* treatment*” OR “gastro protect* therap*” OR “gastrointestinal prophylaxis” OR “gastro intestinal prophylaxis” OR “peptic ulcer prophylaxis” OR “stress ulcer prophylaxis” OR “stress ulcer prophylactic” OR “acid suppressive” OR “acid suppression” OR “acid suppressant*” OR “acid suppressing” OR “acid suppressor*” OR “antacid*”) **NEAR/3** (“appropriate*” OR “no indication*”

	OR "utilization*" OR "utilisation*" OR "justif*" OR "error*" OR "inadequate*" OR "excess*" OR "suboptimal*" OR "inadvertent*" OR "rational*" OR "harmful" OR "long term" OR "longterm" OR "risk*" OR "adverse" OR "optimal" OR "reasonable" OR "reasonably" OR "cost" OR "costs" OR "costly" OR "costing"))
23	21 OR 22
24	TS=("deprescri*" OR "de prescri*" OR "unprescri*" OR "un prescri*" OR "discontinu*" OR "reduction*" OR "step* down" OR "stepwise" OR "step wise" OR "step off" OR "compliance" OR "adherence" OR "audit" OR "audits" OR "taper*" OR "benchmark*" OR "bench mark*" OR "TQM" OR "academic detailing")
25	TS=("Quality" near/1 ("control" OR "improvement*" OR "assurance" OR "assess*" OR "review*" OR "prescri*" OR "guideline*" OR "protocol*" OR "standard*" OR "policy" OR "policies" OR "regulation*" OR "management" OR "intervention" OR "strategy"))
26	TS=(("Protocol*" OR "Guideline*" OR "policy" OR "policies" OR "regulation*") near/3 ("compliance" OR "adhere*" OR "implement*" OR "evaluat*" OR "comply" OR "compliant" OR "concord*" OR "distribut*" OR "disseminat*" OR "best practice*" OR "effective*" OR "evidence base*" OR "EBM" OR "introduce*" OR "issu*" OR "impact*" OR "assess*" OR "review*" OR "adhering" OR "complying"))
27	TS=("Pharmacy" OR "Pharmacies" OR "pharmacotherapeutic*" OR "pharmd" OR pharmacist*)
28	TS=("pharmaceutical" Near/2 ("care" OR "service*" OR "consult*" OR "advice" OR "advise*" OR "advising" OR "intervention"))
29	TS=("Screening test*" OR "screening tool*" OR "systematic tool*" OR "Stopp" OR "start criteria" OR "beers" OR "Pim Check" OR "priscus" OR "medication appropriateness index" OR "FORTA" OR "mcleod*" OR "zhan" OR "ACOVE" OR "pim list*" OR "pim criteria" OR "mai")
30	TS=(("record*" OR "medicine*" OR "chart*" OR "drug" OR "drugs" OR "dose*" OR "dosage*" OR "utilisation" OR "utilization" OR "usage" OR

	"medication*" OR "discharge" OR "prescri*" OR "appropriateness") NEAR/3 ("monitor*" OR "management" OR "reconcile*" OR "review*" OR "evaluat*" OR "strategies" OR "strategy" OR "evidence base*" OR "EBM" OR "assess*"))
31	TS=(("cost" OR "costs") near/2 ("control" OR "savings" OR "analysis" OR "effective*"))
32	TS=(("reduce*" OR "reducing" OR "decrease*" OR "decreasing" OR "modif*" OR "alter*" OR "adapt*" OR "change*" OR "changing" OR "curtail*" OR "revise*" OR "revising" OR "revision" OR "adjust*" OR "deescalate*" OR "de escalate*" OR "lower*" OR "fewer" OR "minimize*" OR "minimise*" OR "minimizing" OR "minimising" OR "eliminating" OR "eliminate*" OR "elimination" OR "cessation" OR "cease*" OR "ceasing" OR "withheld" OR "withhold*" OR "withdraw*" OR "withdrew" OR "stop*" OR "review*" OR "curb*" OR "evaluat*" OR "assess*" OR "monitor*") near/3 ("usage" OR "use" OR "dosage*" OR "dose*" OR "dosing" OR "medication*" OR "medicine*" OR "prescri*" OR "on demand" OR "intermittent" OR "drug" OR "drugs" OR "regimen*" OR "cost" OR "costs" OR "error*" OR "rate*" OR "polypharm*" OR "poly pharm*" OR "polymedicat*" OR "poly medicat*" OR "long term" OR "longterm" OR "duration" OR "PIM" OR "PIMs" OR "pim s" OR "PPI" OR "PPIS" OR "ppi s" OR "sup" OR "ast" OR "asm" OR "asms" OR "asm s" OR "inappropriate*" OR "nonappropriat*" OR "non appropriat*" OR "overprescri*" OR "over prescri*" OR "overmedicat*" OR "over medicat*" OR "overus*" OR "over use" OR "mistreat*" OR "overtreat*" OR "overutili*" OR "over utili*" OR "improper*" OR "incorrect*" OR "unwarranted" OR "unjustified" OR "erroneous*" OR "misus*" OR "un necessar*" OR "unnecessar*" OR "utilization" OR "utilisation" OR "practice pattern*"))
33	TS=(("proton pump inhibitor*" OR "ppi" OR "ppis" OR "ppi s" OR "abeprazan" OR "benatoprazole" OR "tenatoprazole" OR "TU 199" OR "CAS 113712-98-4" OR "stu-na" OR "esomeprazole" OR "esotrex" OR "alenia" OR "escz" OR "esofag" OR "nexiam" OR "ilaprazole" OR "lansoprazole" OR "lansoprazole" OR "agopton" OR "bamalite" OR "inhibitol" OR "levant" OR "lupizole" OR "lanzor" OR "monolitum" OR

	“ogast” OR “ogastro” OR “opiren” OR “prevacid” OR “prezal” OR “pro ulco” OR “promeco” OR “takepron” OR “ulpax” OR “zoton” OR “dexlansoprazole” OR “kapidex” OR “dexilant” OR “levolansoprazole” OR “leminoprazole” OR “linaprazan” OR “nepaprazole” OR “omeprazole” OR “losec” OR “nexium” OR “prilosec” OR “rapinex" OR “zegerid” OR “ocid” OR “lomac” OR “omepral” OR “omez” OR “pantoprazole” OR “protium” OR “protonix” OR “pantotab” OR “pantopan” OR “pantozol” OR “pantor” OR “pantoloc” OR “astropan” OR “controloc” OR “pantecta” OR “inipomp” OR “somac” OR “pantodac” OR “zurcal” OR “zentro” OR “picoprazole” OR “potassium competitive acid blocker” OR “pumaprazole” OR “rabeprazole” OR “aciphex” OR “dexrabeprazole” OR “pariet” OR “zechin” OR “rabecid” OR “nzole-d” OR “rabeloc” OR “revaprazan” OR “saviprazole” OR “soraprazan” OR “tegoprazan” OR “timoprazole” OR “vonoprazan” OR “antiulcer agent*” OR “antiulcer drug*” OR “antiulcer medication*” OR “antiulcer* medicine*” OR “antiulcer treatment*” OR “antiulcer* therap*” OR “anti ulcer* agent*” OR “anti ulcer* drug*” OR “anti ulcer* medication*” OR “anti ulcer* medicine*” OR “anti ulcer* treatment*” OR “anti ulcer* therap*” OR “gastrointestinal agent*” OR “gastrointestinal drug*” OR “gastrointestinal medication*” OR “gastrointestinal medicine*” OR “gastro intestinal agent*” OR “gastro intestinal drug*” OR “gastro intestinal medication*” OR “gastro intestinal medicine*” OR “antireflux agent*” OR “antireflux drug*” OR “antireflux medication*” OR “antireflux medicine*” OR “antireflux treatment*” OR “antireflux therap*” OR “anti reflux agent*” OR “anti reflux drug*” OR “anti reflux medication*” OR “anti reflux medicine*” OR “anti reflux treatment*” OR “anti reflux therap*” OR “gastroesophageal agent*” OR “gastroesophageal drug*” OR “gastroesophageal medication*” OR “gastroesophageal medicine*” OR “gastroesophageal treatment*” OR “gastroesophageal therap*” OR “gastro esophageal agent*” OR “gastro esophageal drug*” OR “gastro esophageal medication*” OR “gastro esophageal medicine*” OR “gastro esophageal treatment*” OR “gastro esophageal therap*” OR “gastroprotect* agent*” OR “gastroprotect* drug*” OR “gastroprotect* medication*” OR “gastroprotect* medicine*” OR “gastroprotect* treatment*” OR “gastroprotect* therap*” OR “gastro protect* agent*” OR “gastro protect* drug*” OR “gastro protect* medication*” OR “gastro protect* medicine*” OR “gastro protect* treatment*” OR “gastro protect* therap*” OR “gastrointestinal

	prophylaxis” OR “gastro intestinal prophylaxis” OR “peptic ulcer prophylaxis” OR “stress ulcer prophylaxis” OR “stress ulcer prophylactic” OR “acid suppressive” OR “acid suppression” OR “acid suppressant*” OR “acid suppressing” OR “acid suppressor*” OR “antacid*”) NEAR/3 (“reduce*” OR “reducing” OR “decrease*” OR “decreasing” OR “modif*” OR “alter*” OR “change*” OR “changing” OR “curtail*” OR “revise*” OR “revising” OR “revision” OR “adjust*” OR “deescalate*” OR “de escalate*” OR “lower*” OR “fewer” OR “minimize*” OR “minimise*” OR “minimizing” OR “minimising” OR “eliminate*” OR “elimination” OR “cessation” OR “cease*” OR “ceasing” OR “withheld” OR “withhold*” OR “withdraw*” OR “withdrew” OR “stop*” OR “monitor*” OR “review*” OR “curb*” OR “evaluat*” OR “assess*” OR “manage*”))
34	OR/24-33
35	23 AND 34
