# Supplementary material for: Impact of pharmacy-supported interventions on proportion of patients receiving non-indicated acid suppressive therapy upon discharge: A systematic review and meta-analysis
Source: PLoS One. 2020 Dec 3;15(12):e0243134. doi: 10.1371/journal.pone.0243134 (PMC7714117; doi:10.1371/journal.pone.0243134)
Supplement: S3 File — (PDF) [file pone.0243134.s003.pdf]

Supplement 3. Funnel plot analysis methodology

Publication bias and study heterogeneity can lead to inaccurate estimate of the effect size. Publication bias detection and remediation has been reviewed by Rothstein et al [1], while study heterogeneity has been reviewed by several authors [2-3]. Methods range from a graphical examination of a funnel plot to selection models and sensitivity analysis. A funnel plot is a simple scatter plot of the intervention effect estimates (on the x-axis) for individual studies plotted against a measure of a study's sample size or calculated variance. The inverted funnel shape graphically illustrates that the effect estimates from small studies will be scattered at the bottom of the plot, and the more robust larger studies appear near the top. Publication bias can be detected by examining the plot for symmetry and, any asymmetrical sections indicate possible bias.

Funnel plots have been used to examine and detect publication bias; correcting for publication bias requires the trim and fill method or more sophisticated selection models [1]. Duval and Tweedie discuss a quantitative modeling method based on a meta-analyzed estimate of effect using the DerSimonian-Laird estimator [4]. From the DerSimonian-Laird estimator and statistical moment theory, three estimators of publication bias were derived: R_0, L_0 and Q_0. The estimator equations provide an estimate of the number of studies missing from the meta-analysis, which are then added to the analysis via simulation. When applied to our data, the R_0 estimate recommended addition of one study, L_0 four studies and Q_0 three studies. We did not add any simulated studies to our analysis. We did assess publication bias graphically using the optimized funnel plot as discussed below.

Funnel plots can also aid in reducing study heterogeneity, although to our knowledge they have not been used in this fashion. Our funnel plot optimization method to reduce heterogeneity is similar to model fitting in Non-linear-mixed-effects modeling. The meta-analysis is conducted with all selected studies and a funnel plot is produced. The overall odds ratio (OR)

estimate and the individual study ORs are then plotted along with respective 95% confidence intervals (CI) of the effect size. The y-axis is the meta-analysis weight of the study. The plot is graphically examined, and studies where the CI does not overlap the overall effect size CI are eligible for removal. If more than one study meets the criteria, the study whose OR point estimate has the largest difference from the overall OR is selected for removal. Upon removal, the meta-analysis is conducted again and the new combined OR and CI are now plotted with the individual OR and CI and the new study weight. The combined overall OR, CI and study weight will change, however, the individual study OR and CI will not change. A second funnel plot is produced, and the process continues until there are no more studies to remove, or the individual study's OR and CI overlap with the combined OR and CI. In the example given below, Carey et al would be removed.

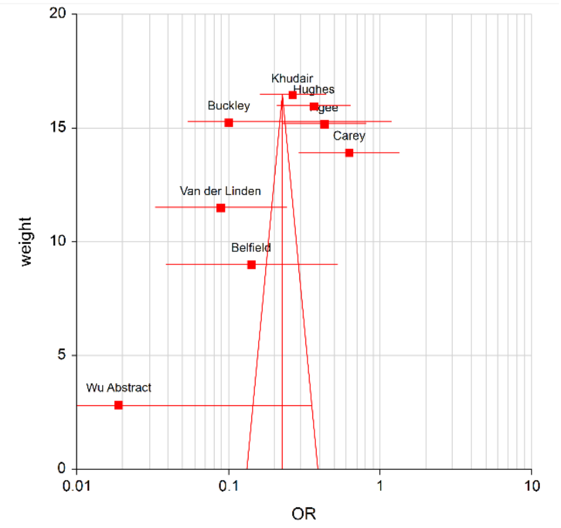

Our method extends meta-analysis funnel plot capability from purely detecting heterogeneity to remediating heterogeneity. We have reduced I^2 from ~90% to ~40% using this method. Our funnel optimization results are compared with the radial plot method in the graphs that follow. Note how the studies outside of the radial plot analysis are removed with our funnel method.

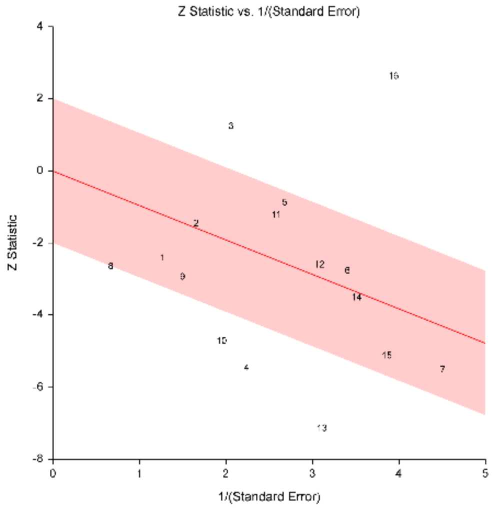

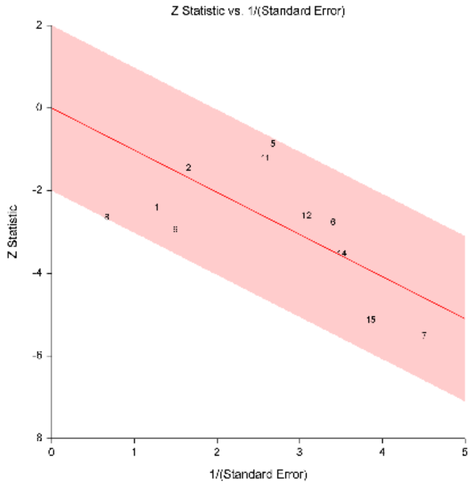

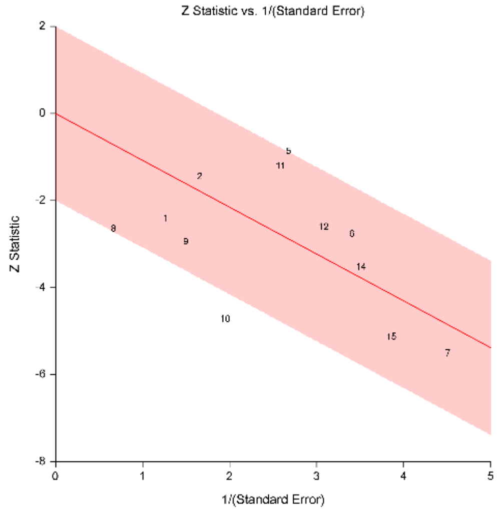

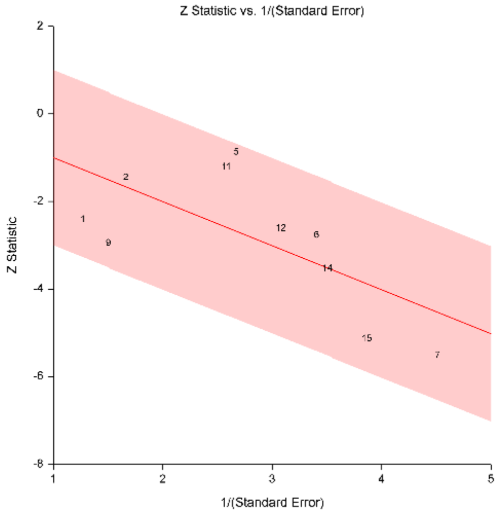

Table. Study number and author

1. Martz ICU	2. Tasaka ICU	3. Zeigler ICU	4. Buckley ICU
5. Hammond ICU	6. Pavlov ICU	7. Wohlt-Hatch ICU	8. Wu non-ICU
9. Belfield non-ICU	10. Van der Linden non-ICU	11. Carey non-ICU	12. Agee non-ICU
13. Buckley non-ICU	14. Hughes non-ICU	15. Khudair non-ICU	16. Ziegler non-ICU

References

[1] Rothstein HR, Sutton AJ, Borenstein M, editors. Publication bias in meta-analysis: prevention, assessment and adjustments. Chichester, England: John Wiley & Sons Ltd; 2005.

[2] Higgins JPT, Thompson SG. Quantifying heterogeneity in a meta-analysis. Statistics in Medicine. 2002;21:1539-1558.

[3] Ioannidis JP, Patsopoulos NA, Evangelou E. Uncertainty in heterogeneity estimates in meta-analyses. BMJ. 2007;335(7626):914–916.

[4] Duval SJ, Tweedie RL. A nonparametric "trim and fill" method of accounting for publication bias in meta-analysis. Journal of the American Statistical Association. 2000;95(449):89-98.
