# Supplementary material for: Impact of pharmacy-supported interventions on proportion of patients receiving non-indicated acid suppressive therapy upon discharge: A systematic review and meta-analysis
Source: PLoS One. 2020 Dec 3;15(12):e0243134. doi: 10.1371/journal.pone.0243134 (PMC7714117; doi:10.1371/journal.pone.0243134)
Supplement: S4 File — (PDF) [file pone.0243134.s004.pdf]

Supplement 4. Abbreviated description of pharmacist-supported interventions	
**ICU studies**	
Buckley et al [65]	Pharmacists had prescriptive authority to initiate, modify, or discontinue SUP within the context of the defined institutional protocol using computerized provider order entry within ICU and non-ICU settings
Hammond et al [67]	1. Development and distribution of pocket cards describing appropriate SUP<br>2. Education of clinical staff (medical residents/fellows) on appropriate SUP<br>3. Daily rounding (5 days/week)
Martz et al [72]	Pharmacist-driven protocol used to discontinue temporary medications in ICU
Pavlov et al [73]	Emergency-room pharmacy technicians conducted comprehensive medication reconciliation on admission
Tasaka et al [74]	1. Guideline development and distribution on SUP prophylaxis<br>2. Education of clinical staff<br>3. Daily rounding
Hatch et al [68]	1. Distributed previously-developed SUP guidelines electronically and as pocket cards<br>2. Daily rounding<br>3. Medication reconciliation at all care transfer
Zeigler et al [77]	Education of all clinical staff, including pharmacists and nurses, on the medication reconciliation process (not about appropriate SUP use), which can be completed throughout the continuum of care
**Non-ICU studies**	
Agee et al [63]	1. Educational seminar describing appropriate SUP indications<br>2. Distribution of pocket cards outlining appropriate SUP indications
Belfield et al [64]	1. Guidelines development (institutional stress-related mucosal disease prophylaxis)<br>2. Education (weekly) to hospitalists on appropriate AST<br>3. Reviewed AST orders from daily census and contacted hospitalists as needed for clarification
Buckley et al [65]	Pharmacists had prescriptive authority to make medication changes (as above)
Carey et al [66]	Pharmacy students:<br>1. Evaluated medication lists daily and contacted medicine team for clarification<br>2. Made recommendations during daily rounds (recommendations later verified by pharmacist preceptor)
Hughes et al [69]	Participated in weekly interdisciplinary rounds to make recommendations on AST prescribing
Khudair et al [70]	1. Development and implementation of an AST-usage guideline<br>2. Distribution of flyer explaining when AST should be addressed<br>3. Daily rounding (5 days/week)
Van der Linden et al [79]	1. Applied RASP list to medication lists upon admission and at discharge<br>2. Comprehensive medication reconciliation if inappropriate meds detected<br>3. Daily rounds along with daily recommendations to physician
Wu et al [76]	Medication reconciliation with recommendations to “deprescribe” PPIs as necessary
Ziegler et al [78]	1. Guidelines development and dissemination<br>2. Continual education to clinical staff on appropriate AST use<br>3. Daily rounding
