# Supplementary material for: Impact of pharmacy-supported interventions on proportion of patients receiving non-indicated acid suppressive therapy upon discharge: A systematic review and meta-analysis
Source: PLoS One. 2020 Dec 3;15(12):e0243134. doi: 10.1371/journal.pone.0243134 (PMC7714117; doi:10.1371/journal.pone.0243134)

Supplement 5. Proportion of patients discharged on inappropriate AST from the hospital: Subgroup analyses

Subgroup 1. Studies conducted in the United States versus other countries

Study or Subgroup	Intervention Events	Intervention Total	Usual Care Events	Usual Care Total	Weight	Odds Ratio M-H, Random, 95% CI
**1.17.1 USA**						
Wu Abstract non-ICU	0	21	10	18	2.1%	0.02 [0.00, 0.36]
Martz Abstract ICU	2	57	12	62	4.5%	0.15 [0.03, 0.71]
Belfield non-ICU	3	81	13	61	5.1%	0.14 [0.04, 0.52]
Tasaka ICU	4	44	14	73	5.5%	0.42 [0.13, 1.37]
Zeigler ICU	15	61	8	53	6.2%	1.83 [0.71, 4.75]
Buckley ICU	6	167	52	174	6.4%	0.09 [0.04, 0.21]
Carey non-ICU	12	197	18	192	6.7%	0.63 [0.29, 1.34]
Hammond ICU	16	118	18	101	6.8%	0.72 [0.35, 1.51]
Agee non-ICU	15	193	36	220	7.1%	0.43 [0.23, 0.81]
Buckley non-ICU	11	204	213	589	7.1%	0.10 [0.05, 0.19]
Pavlov ICU	20	291	36	253	7.2%	0.44 [0.25, 0.79]
Hughes non-ICU	28	117	54	117	7.3%	0.37 [0.21, 0.64]
Ziegler non-ICU	54	126	43	155	7.4%	1.95 [1.19, 3.21]
Wohlt-Hatch ICU	31	356	96	394	7.5%	0.30 [0.19, 0.46]
**Subtotal (95% CI)**		**2033**		**2462**	**86.6%**	**0.36 [0.21, 0.62]**
Total events	217		623			
Heterogeneity: Tau² = 0.83; Chi² = 94.16, df = 13 (P < 0.00001); I² = 86%						
Test for overall effect: Z = 3.69 (P = 0.0002)						
**1.17.2 Other countries**						
Van der Linden non-ICU	5	91	32	81	6.0%	0.09 [0.03, 0.24]
Khudair non-ICU	26	194	70	190	7.4%	0.27 [0.16, 0.44]
**Subtotal (95% CI)**		**285**		**271**	**13.4%**	**0.17 [0.06, 0.48]**
Total events	31		102			
Heterogeneity: Tau² = 0.44; Chi² = 3.64, df = 1 (P = 0.06); I² = 72%						
Test for overall effect: Z = 3.30 (P = 0.0010)						
**Total (95% CI)**		**2318**		**2733**	**100.0%**	**0.33 [0.20, 0.53]**
Total events	248		725			
Heterogeneity: Tau² = 0.78; Chi² = 105.18, df = 15 (P < 0.00001); I² = 86%						
Test for overall effect: Z = 4.49 (P < 0.00001)						
Test for subgroup differences: Chi² = 1.59, df = 1 (P = 0.21), I² = 37.0%						

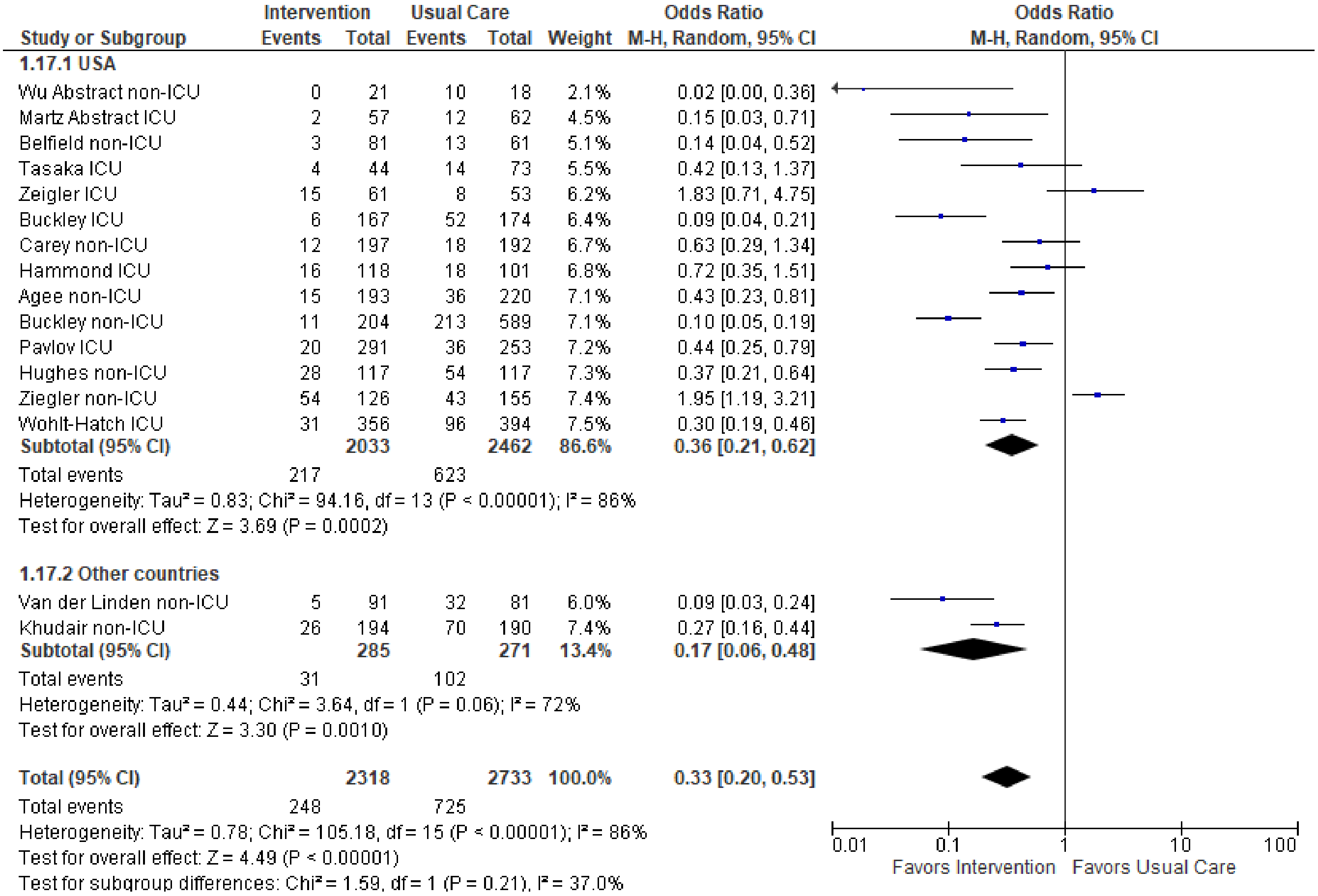

Subgroup 2. Study design (retrospective versus prospective)

Study or Subgroup	Intervention Events	Intervention Total	Usual Care Events	Usual Care Total	Weight	Odds Ratio M-H, Random, 95% CI
**1.10.1 Retrospective**						
Martz Abstract ICU	2	57	12	62	4.5%	0.15 [0.03, 0.71]
Belfield non-ICU	3	81	13	61	5.1%	0.14 [0.04, 0.52]
Tasaka ICU	4	44	14	73	5.5%	0.42 [0.13, 1.37]
Zeigler ICU	15	61	8	53	6.2%	1.83 [0.71, 4.75]
Buckley ICU	6	167	52	174	6.4%	0.09 [0.04, 0.21]
Hammond ICU	16	118	18	101	6.8%	0.72 [0.35, 1.51]
Agee non-ICU	15	193	36	220	7.1%	0.43 [0.23, 0.81]
Buckley non-ICU	11	204	213	589	7.1%	0.10 [0.05, 0.19]
Pavlov ICU	20	291	36	253	7.2%	0.44 [0.25, 0.79]
Hughes non-ICU	28	117	54	117	7.3%	0.37 [0.21, 0.64]
Ziegler non-ICU	54	126	43	155	7.4%	1.95 [1.19, 3.21]
Wohlt-Hatch ICU	31	356	96	394	7.5%	0.30 [0.19, 0.46]
**Subtotal (95% CI)**		**1815**		**2252**	**77.9%**	**0.37 [0.21, 0.67]**
Total events	205		595			
Heterogeneity: Tau² = 0.86; Chi² = 88.50, df = 11 (P < 0.00001); I² = 88%						
Test for overall effect: Z = 3.34 (P = 0.0008)						
**1.10.2 Prospective**						
Wu Abstract non-ICU	0	21	10	18	2.1%	0.02 [0.00, 0.36]
Van der Linden non-ICU	5	91	32	81	6.0%	0.09 [0.03, 0.24]
Carey non-ICU	12	197	18	192	6.7%	0.63 [0.29, 1.34]
Khudair non-ICU	26	194	70	190	7.4%	0.27 [0.16, 0.44]
**Subtotal (95% CI)**		**503**		**481**	**22.1%**	**0.21 [0.08, 0.54]**
Total events	43		130			
Heterogeneity: Tau² = 0.65; Chi² = 12.74, df = 3 (P = 0.005); I² = 76%						
Test for overall effect: Z = 3.19 (P = 0.001)						
**Total (95% CI)**		**2318**		**2733**	**100.0%**	**0.33 [0.20, 0.53]**
Total events	248		725			
Heterogeneity: Tau² = 0.78; Chi² = 105.18, df = 15 (P < 0.00001); I² = 86%						
Test for overall effect: Z = 4.49 (P < 0.00001)						
Test for subgroup differences: Chi² = 1.06, df = 1 (P = 0.30), I² = 5.3%						

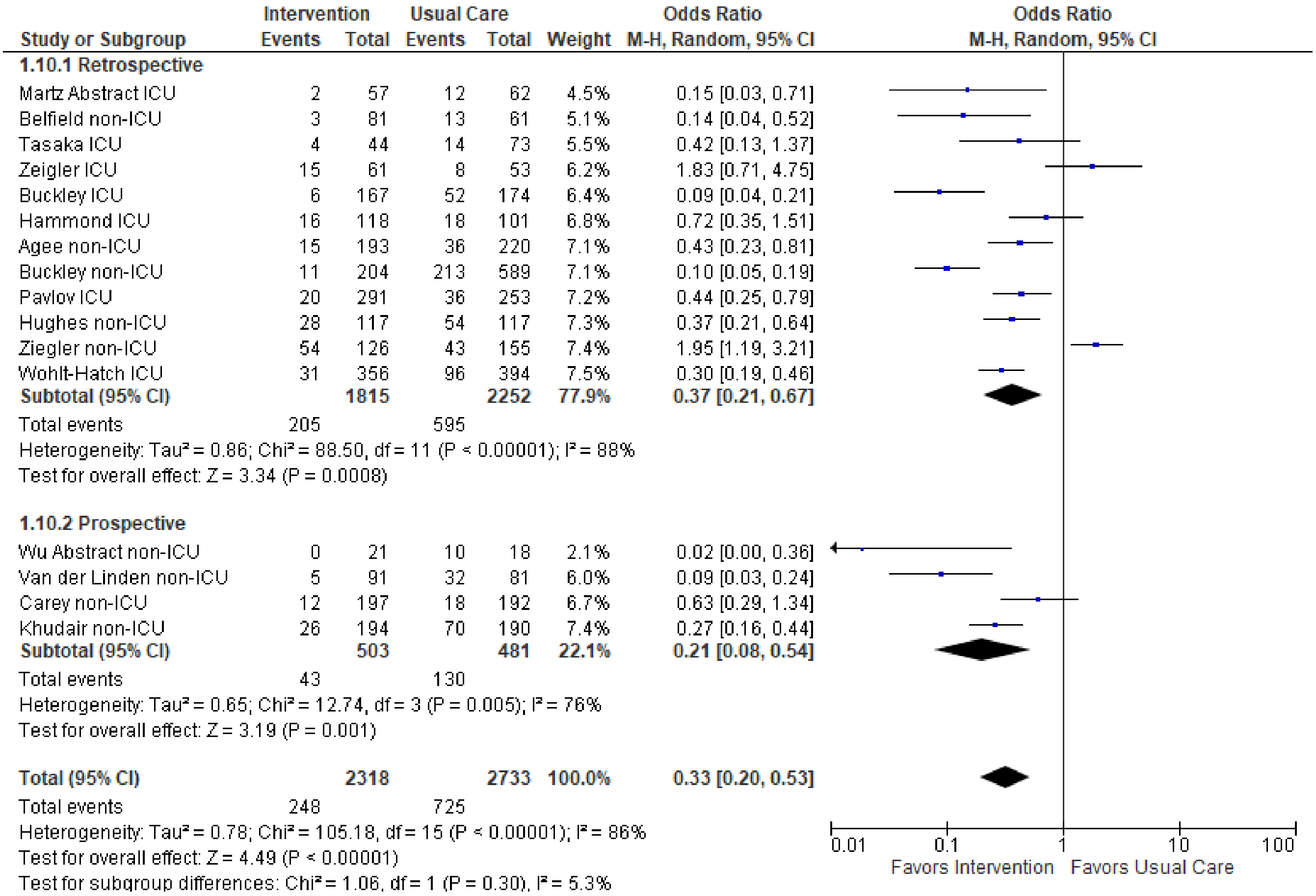

Subgroup 3. Study settings (medical floor/general ward versus other settings)

Study or Subgroup	Intervention Events	Intervention Total	Usual Care Events	Usual Care Total	Weight	Odds Ratio M-H, Random, 95% CI
**1.18.1 Medical Floor / General Ward**						
Wu Abstract non-ICU	0	21	10	18	2.1%	0.02 [0.00, 0.36]
Martz Abstract ICU	2	57	12	62	4.5%	0.15 [0.03, 0.71]
Belfield non-ICU	3	81	13	61	5.1%	0.14 [0.04, 0.52]
Tasaka ICU	4	44	14	73	5.5%	0.42 [0.13, 1.37]
Zeigler ICU	15	61	8	53	6.2%	1.83 [0.71, 4.75]
Buckley ICU	6	167	52	174	6.4%	0.09 [0.04, 0.21]
Carey non-ICU	12	197	18	192	6.7%	0.63 [0.29, 1.34]
Hammond ICU	16	118	18	101	6.8%	0.72 [0.35, 1.51]
Agee non-ICU	15	193	36	220	7.1%	0.43 [0.23, 0.81]
Buckley non-ICU	11	204	213	589	7.1%	0.10 [0.05, 0.19]
Pavlov ICU	20	291	36	253	7.2%	0.44 [0.25, 0.79]
Khudair non-ICU	26	194	70	190	7.4%	0.27 [0.16, 0.44]
Wohlt-Hatch ICU	31	356	96	394	7.5%	0.30 [0.19, 0.46]
**Subtotal (95% CI)**		**1984**		**2380**	**79.4%**	**0.31 [0.20, 0.48]**
Total events	161		596			
Heterogeneity: Tau² = 0.47; Chi² = 52.08, df = 12 (P < 0.00001); I² = 77%						
Test for overall effect: Z = 5.10 (P < 0.00001)						
**1.18.2 Other settings**						
Van der Linden non-ICU	5	91	32	81	6.0%	0.09 [0.03, 0.24]
Hughes non-ICU	28	117	54	117	7.3%	0.37 [0.21, 0.64]
Ziegler non-ICU	54	126	43	155	7.4%	1.95 [1.19, 3.21]
**Subtotal (95% CI)**		**334**		**353**	**20.6%**	**0.42 [0.08, 2.11]**
Total events	87		129			
Heterogeneity: Tau² = 1.91; Chi² = 37.85, df = 2 (P < 0.00001); I² = 95%						
Test for overall effect: Z = 1.05 (P = 0.29)						
**Total (95% CI)**		**2318**		**2733**	**100.0%**	**0.33 [0.20, 0.53]**
Total events	248		725			
Heterogeneity: Tau² = 0.78; Chi² = 105.18, df = 15 (P < 0.00001); I² = 86%						
Test for overall effect: Z = 4.49 (P < 0.00001)						
Test for subgroup differences: Chi² = 0.13, df = 1 (P = 0.72), I² = 0%						

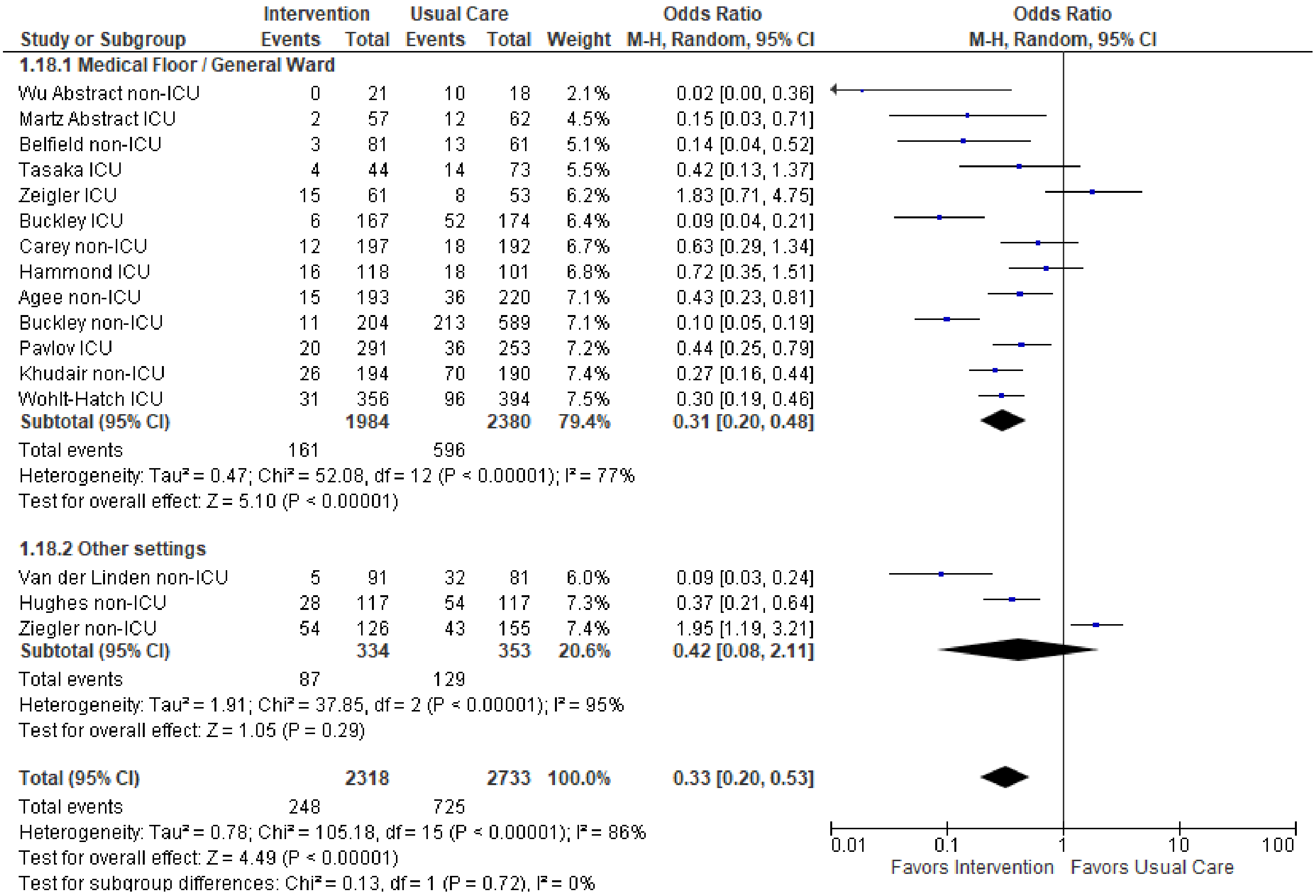

Subgroup 4. Studies that included patients on AST prior to arrival versus those that excluded patients on AST prior to arrival

Study or Subgroup	Intervention Events	Intervention Total	Usual Care Events	Usual Care Total	Weight	Odds Ratio M-H, Random, 95% CI
**1.13.1 AST PTA Included**						
Wu Abstract non-ICU	0	21	10	18	2.1%	0.02 [0.00, 0.36]
Tasaka ICU	4	44	14	73	5.5%	0.42 [0.13, 1.37]
Van der Linden non-ICU	5	91	32	81	6.0%	0.09 [0.03, 0.24]
Carey non-ICU	12	197	18	192	6.7%	0.63 [0.29, 1.34]
Agee non-ICU	15	193	36	220	7.1%	0.43 [0.23, 0.81]
Pavlov ICU	20	291	36	253	7.2%	0.44 [0.25, 0.79]
Hughes non-ICU	28	117	54	117	7.3%	0.37 [0.21, 0.64]
Khudair non-ICU	26	194	70	190	7.4%	0.27 [0.16, 0.44]
Ziegler non-ICU	54	126	43	155	7.4%	1.95 [1.19, 3.21]
Wohlt-Hatch ICU	31	356	96	394	7.5%	0.30 [0.19, 0.46]
**Subtotal (95% CI)**		**1630**		**1693**	**64.1%**	**0.38 [0.22, 0.65]**
Total events	195		409			
Heterogeneity: $Tau^2 = 0.57$; $Chi^2 = 56.87$, df = 9 ($P < 0.00001$); $I^2 = 84\%$						
Test for overall effect: $Z = 3.54$ ($P = 0.0004$)						
**1.13.2 AST PTA Excluded**						
Martz Abstract ICU	2	57	12	62	4.5%	0.15 [0.03, 0.71]
Belfield non-ICU	3	81	13	61	5.1%	0.14 [0.04, 0.52]
Zeigler ICU	15	61	8	53	6.2%	1.83 [0.71, 4.75]
Buckley ICU	6	167	52	174	6.4%	0.09 [0.04, 0.21]
Hammond ICU	16	118	18	101	6.8%	0.72 [0.35, 1.51]
Buckley non-ICU	11	204	213	589	7.1%	0.10 [0.05, 0.19]
**Subtotal (95% CI)**		**688**		**1040**	**35.9%**	**0.26 [0.09, 0.75]**
Total events	53		316			
Heterogeneity: $Tau^2 = 1.51$; $Chi^2 = 40.42$, df = 5 ($P < 0.00001$); $I^2 = 88\%$						
Test for overall effect: $Z = 2.49$ ($P = 0.01$)						
**Total (95% CI)**		**2318**		**2733**	**100.0%**	**0.33 [0.20, 0.53]**
Total events	248		725			
Heterogeneity: $Tau^2 = 0.78$; $Chi^2 = 105.18$, df = 15 ($P < 0.00001$); $I^2 = 86\%$						
Test for overall effect: $Z = 4.49$ ($P < 0.00001$)						
Test for subgroup differences: $Chi^2 = 0.42$, df = 1 ($P = 0.52$), $I^2 = 0\%$						

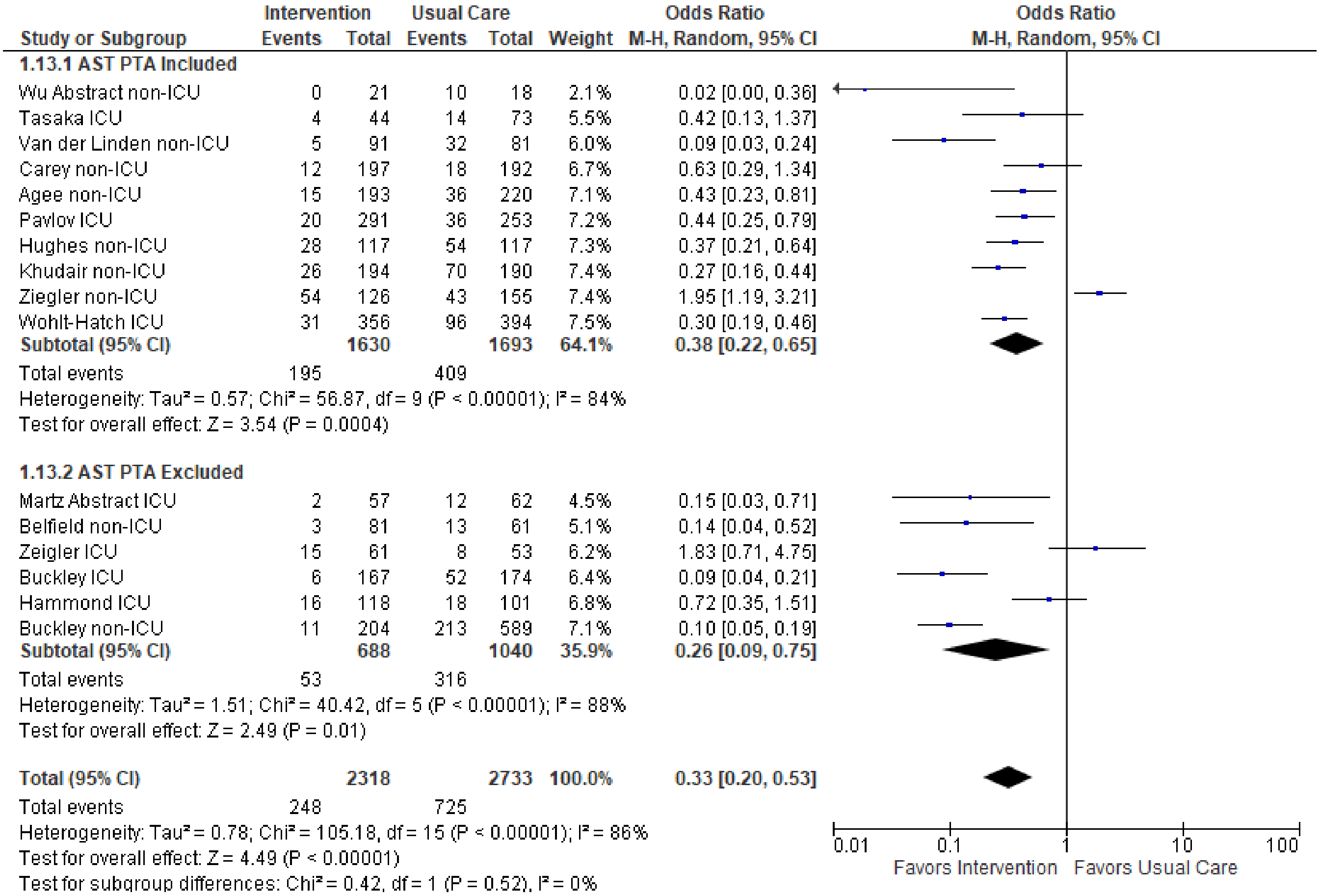

Subgroup 5. Data collection observation periods (≤ 15 weeks versus > 15 weeks)

Study or Subgroup	Intervention Events	Intervention Total	Usual Care Events	Usual Care Total	Weight	Odds Ratio M-H, Random, 95% CI
**1.15.1 Observation Periods ≤ 15 Weeks**						
Wu Abstract non-ICU	0	21	10	18	2.1%	0.02 [0.00, 0.36]
Martz Abstract ICU	2	57	12	62	4.5%	0.15 [0.03, 0.71]
Belfield non-ICU	3	81	13	61	5.1%	0.14 [0.04, 0.52]
Tasaka ICU	4	44	14	73	5.5%	0.42 [0.13, 1.37]
Zeigler ICU	15	61	8	53	6.2%	1.83 [0.71, 4.75]
Buckley ICU	6	167	52	174	6.4%	0.09 [0.04, 0.21]
Agee non-ICU	15	193	36	220	7.1%	0.43 [0.23, 0.81]
Buckley non-ICU	11	204	213	589	7.1%	0.10 [0.05, 0.19]
Pavlov ICU	20	291	36	253	7.2%	0.44 [0.25, 0.79]
Khudair non-ICU	26	194	70	190	7.4%	0.27 [0.16, 0.44]
Ziegler non-ICU	54	126	43	155	7.4%	1.95 [1.19, 3.21]
Wohlt-Hatch ICU	31	356	96	394	7.5%	0.30 [0.19, 0.46]
**Subtotal (95% CI)**		**1795**		**2242**	**73.2%**	**0.31 [0.17, 0.57]**
Total events	187		603			
Heterogeneity: Tau² = 0.96; Chi² = 92.40, df = 11 (P < 0.00001); I² = 88%						
Test for overall effect: Z = 3.70 (P = 0.0002)						
**1.15.2 Observation Periods > 15 Weeks**						
Van der Linden non-ICU	5	91	32	81	6.0%	0.09 [0.03, 0.24]
Carey non-ICU	12	197	18	192	6.7%	0.63 [0.29, 1.34]
Hammond ICU	16	118	18	101	6.8%	0.72 [0.35, 1.51]
Hughes non-ICU	28	117	54	117	7.3%	0.37 [0.21, 0.64]
**Subtotal (95% CI)**		**523**		**491**	**26.8%**	**0.37 [0.17, 0.79]**
Total events	61		122			
Heterogeneity: Tau² = 0.45; Chi² = 12.63, df = 3 (P = 0.006); I² = 76%						
Test for overall effect: Z = 2.55 (P = 0.01)						
**Total (95% CI)**		**2318**		**2733**	**100.0%**	**0.33 [0.20, 0.53]**
Total events	248		725			
Heterogeneity: Tau² = 0.78; Chi² = 105.18, df = 15 (P < 0.00001); I² = 86%						
Test for overall effect: Z = 4.49 (P < 0.00001)						
Test for subgroup differences: Chi² = 0.13, df = 1 (P = 0.72), I² = 0%						

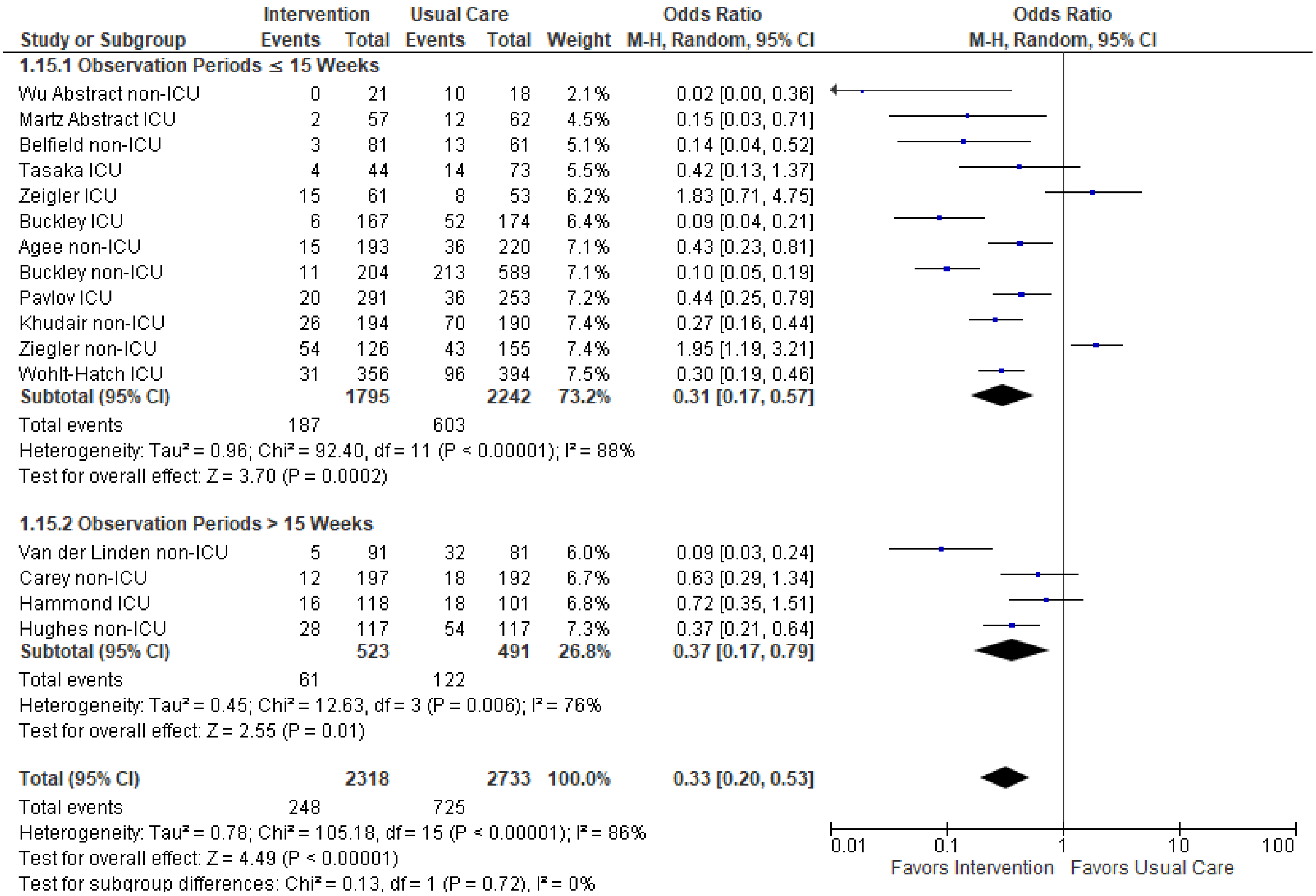

Subgroup 6. Pharmacist interventions (prescribing versus other pharmacist-supported interventions)

	Intervention		Usual Care			Odds Ratio
Study or Subgroup	Events	Total	Events	Total	Weight	M-H, Random, 95% CI
**1.16.1 Prescribing**						
Martz Abstract ICU	2	57	12	62	4.5%	0.15 [0.03, 0.71]
Buckley ICU	6	167	52	174	6.4%	0.09 [0.04, 0.21]
Buckley non-ICU	11	204	213	589	7.1%	0.10 [0.05, 0.19]
**Subtotal (95% CI)**		**428**		**825**	**17.9%**	**0.10 [0.06, 0.16]**
Total events	19		277			
Heterogeneity: Tau² = 0.00; Chi² = 0.37, df = 2 (P = 0.83); I² = 0%						
Test for overall effect: Z = 9.27 (P < 0.00001)						
**1.16.2 Other Interventions**						
Wu Abstract non-ICU	0	21	10	18	2.1%	0.02 [0.00, 0.36]
Belfield non-ICU	3	81	13	61	5.1%	0.14 [0.04, 0.52]
Tasaka ICU	4	44	14	73	5.5%	0.42 [0.13, 1.37]
Van der Linden non-ICU	5	91	32	81	6.0%	0.09 [0.03, 0.24]
Zeigler ICU	15	61	8	53	6.2%	1.83 [0.71, 4.75]
Carey non-ICU	12	197	18	192	6.7%	0.63 [0.29, 1.34]
Hammond ICU	16	118	18	101	6.8%	0.72 [0.35, 1.51]
Agee non-ICU	15	193	36	220	7.1%	0.43 [0.23, 0.81]
Pavlov ICU	20	291	36	253	7.2%	0.44 [0.25, 0.79]
Hughes non-ICU	28	117	54	117	7.3%	0.37 [0.21, 0.64]
Khudair non-ICU	26	194	70	190	7.4%	0.27 [0.16, 0.44]
Ziegler non-ICU	54	126	43	155	7.4%	1.95 [1.19, 3.21]
Wohlt-Hatch ICU	31	356	96	394	7.5%	0.30 [0.19, 0.46]
**Subtotal (95% CI)**		**1890**		**1908**	**82.1%**	**0.42 [0.26, 0.69]**
Total events	229		448			
Heterogeneity: Tau² = 0.58; Chi² = 69.77, df = 12 (P < 0.00001); I² = 83%						
Test for overall effect: Z = 3.50 (P = 0.0005)						
**Total (95% CI)**		**2318**		**2733**	**100.0%**	**0.33 [0.20, 0.53]**
Total events	248		725			
Heterogeneity: Tau² = 0.78; Chi² = 105.18, df = 15 (P < 0.00001); I² = 86%						
Test for overall effect: Z = 4.49 (P < 0.00001)						
Test for subgroup differences: Chi² = 17.07, df = 1 (P < 0.0001), I² = 94.1%						

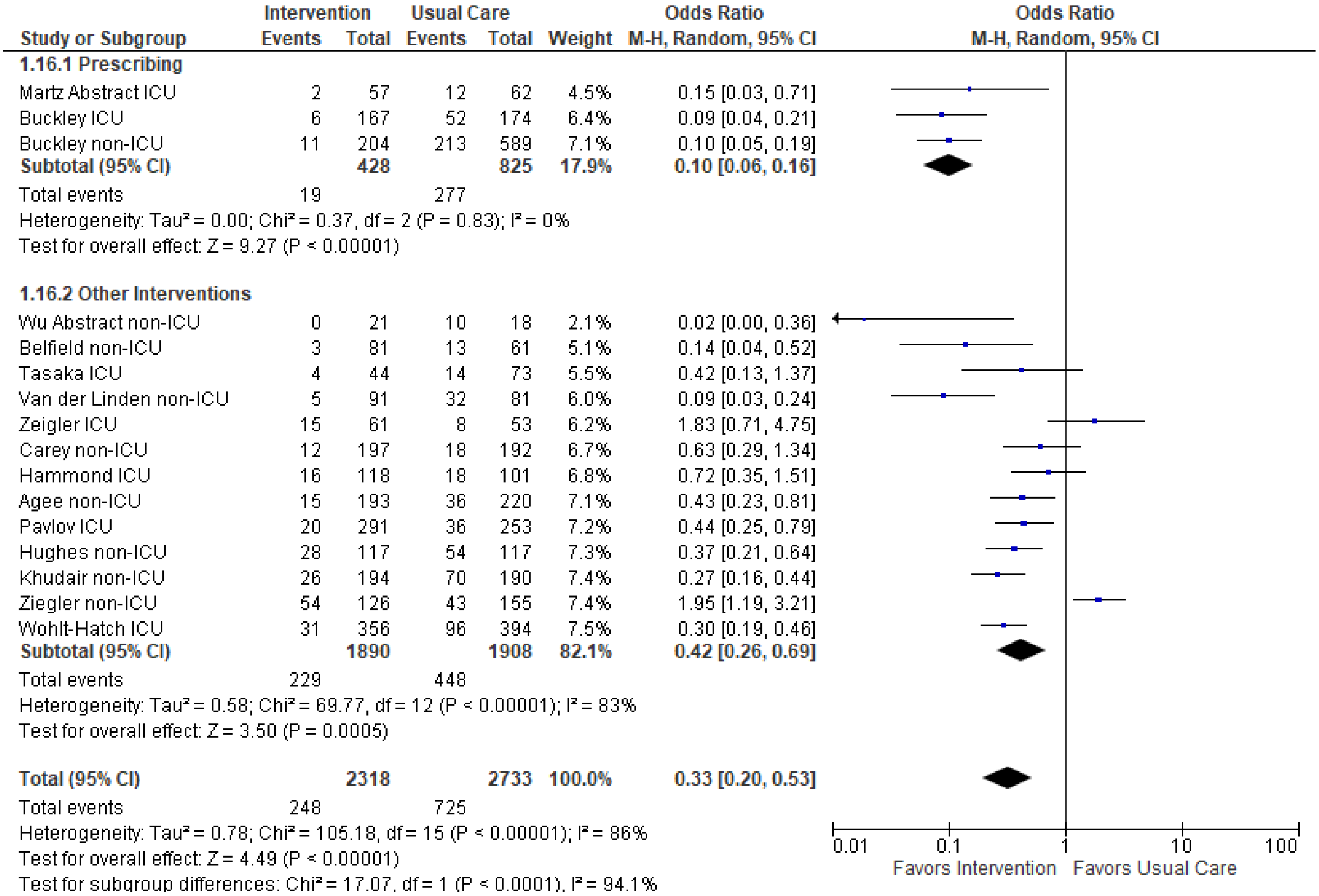

Subgroup 7. Newcastle-Ottawa Scale (total score <7 versus ≥7)

Study or Subgroup	Intervention Events	Intervention Total	Usual Care Events	Usual Care Total	Weight	Odds Ratio M-H, Random, 95% CI
**1.11.1 NOS Score < 7**						
Martz Abstract ICU	2	57	12	62	4.5%	0.15 [0.03, 0.71]
Carey non-ICU	12	197	18	192	6.7%	0.63 [0.29, 1.34]
Wohlt-Hatch ICU	31	356	96	394	7.5%	0.30 [0.19, 0.46]
**Subtotal (95% CI)**		**610**		**648**	**18.7%**	**0.35 [0.19, 0.66]**
Total events	45		126			
Heterogeneity: Tau² = 0.16; Chi² = 3.95, df = 2 (P = 0.14); I² = 49%						
Test for overall effect: Z = 3.22 (P = 0.001)						
**1.11.2 NOS Score ≥ 7**						
Wu Abstract non-ICU	0	21	10	18	2.1%	0.02 [0.00, 0.36]
Belfield non-ICU	3	81	13	61	5.1%	0.14 [0.04, 0.52]
Tasaka ICU	4	44	14	73	5.5%	0.42 [0.13, 1.37]
Van der Linden non-ICU	5	91	32	81	6.0%	0.09 [0.03, 0.24]
Zeigler ICU	15	61	8	53	6.2%	1.83 [0.71, 4.75]
Buckley ICU	6	167	52	174	6.4%	0.09 [0.04, 0.21]
Hammond ICU	16	118	18	101	6.8%	0.72 [0.35, 1.51]
Agee non-ICU	15	193	36	220	7.1%	0.43 [0.23, 0.81]
Buckley non-ICU	11	204	213	589	7.1%	0.10 [0.05, 0.19]
Pavlov ICU	20	291	36	253	7.2%	0.44 [0.25, 0.79]
Hughes non-ICU	28	117	54	117	7.3%	0.37 [0.21, 0.64]
Khudair non-ICU	26	194	70	190	7.4%	0.27 [0.16, 0.44]
Ziegler non-ICU	54	126	43	155	7.4%	1.95 [1.19, 3.21]
**Subtotal (95% CI)**		**1708**		**2085**	**81.3%**	**0.32 [0.17, 0.58]**
Total events	203		599			
Heterogeneity: Tau² = 1.01; Chi² = 100.96, df = 12 (P < 0.00001); I² = 88%						
Test for overall effect: Z = 3.70 (P = 0.0002)						
**Total (95% CI)**		**2318**		**2733**	**100.0%**	**0.33 [0.20, 0.53]**
Total events	248		725			
Heterogeneity: Tau² = 0.78; Chi² = 105.18, df = 15 (P < 0.00001); I² = 86%						
Test for overall effect: Z = 4.49 (P < 0.00001)						
Test for subgroup differences: Chi² = 0.04, df = 1 (P = 0.84), I² = 0%						

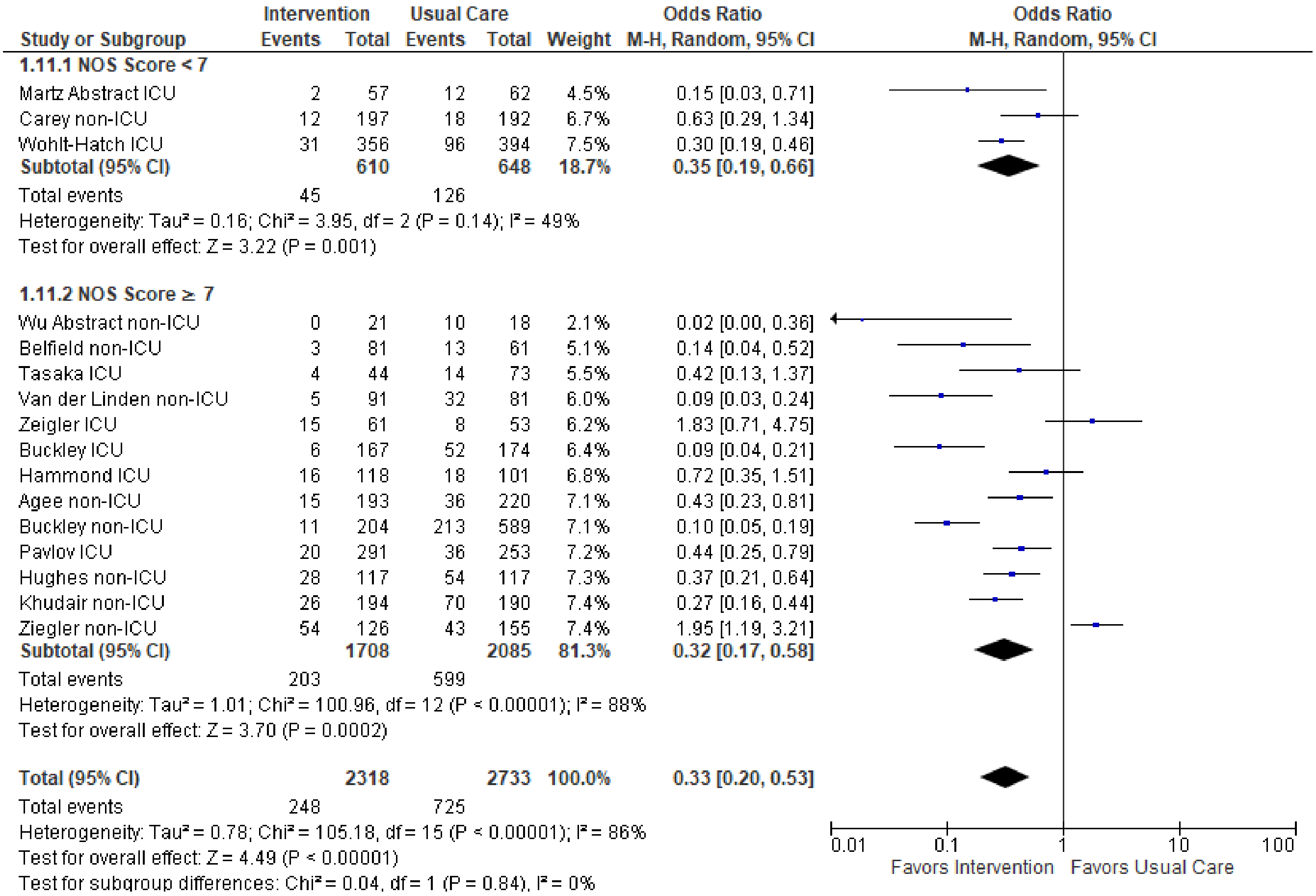

Supplement: S5 File — (PDF) [file pone.0243134.s005.pdf]
